# *Acanthamoeba castellanii* STAT Protein

**DOI:** 10.1371/journal.pone.0111345

**Published:** 2014-10-22

**Authors:** Anna Kicinska, Jacek Leluk, Wieslawa Jarmuszkiewicz

**Affiliations:** 1 Department of Bioenergetics, Adam Mickiewicz University, Poznan, Poland; 2 Department of Molecular Biology, University of Zielona Gora, Zielona Gora, Poland; University of Nebraska-Lincoln, United States of America

## Abstract

STAT (signal transducers and activators of transcription) proteins are one of the important mediators of phosphotyrosine-regulated signaling in metazoan cells. We described the presence of STAT protein in a unicellular, free-living amoebae with a simple life cycle, *Acanthamoeba castellanii*. *A. castellanii* is the only, studied to date, Amoebozoan that does not belong to Mycetozoa but possesses STATs. A sequence of the *A. castellanii* STAT protein includes domains similar to those of the *Dictyostelium* STAT proteins: a coiled coil (characteristic for *Dictyostelium* STAT coiled coil), a STAT DNA-binding domain and a Src-homology domain. The search for protein sequences homologous to *A. castellanii* STAT revealed 17 additional sequences from lower eukaryotes. Interestingly, all of these sequences come from Amoebozoa organisms that belong to either Mycetozoa (slime molds) or Centramoebida. We showed that there are four separated clades within the slime mold STAT proteins. The *A. castellanii* STAT protein branches next to a group of STATc proteins from Mycetozoa. We also demonstrate that Amoebozoa form a distinct monophyletic lineage within the STAT protein world that is well separated from the other groups.

## Introduction

In mammals, seven signal transducer and activator of transcription proteins (STATs), i.e., STAT1–4, STAT5a, STAT5b and STAT6, have been identified [1]. This family of proteins is activated by cytokines or growth factors [2]. A STAT-induced signaling cascade is initiated in response to the extracellular signals [3]. Cytosolic STAT monomers first bind to specific receptors, which then induce receptor-associated Janus kinases (JAKs) to phosphorylate tyrosine residues near the STAT C-termini. Following phosphorylation, STAT molecules form dimers, which then translocate to the nucleus to bind target DNA and to regulate the transcription of target genes.

STAT proteins are also present in other vertebrate species. Orthologs of all the mammalian STAT genes in teleostean fish have been identified, which indicates that these protein families were already largely complete before the teleost-tetrapod split 450 million years ago [4]. STAT proteins are also found in several invertebrates. In the sea squirt (*Ciona intestinalis*), which is a key species in chordate evolution because it represents one of the closest invertebrate relatives to the vertebrate subphylum, only two *STAT* genes (*STAT*-a and *STAT*-b) have been identified [5]. There are also two STAT proteins in *Caenorhabditis elegans* [6] and one in *Drosophila melanogaster*. Moreover, four STAT isoforms are present in belonging to the Amoebozoa slime mold *Dictyostellium discoideum*. The fact that this simple eukaryote possesses STAT proteins indicates that this family of proteins must have arisen early in evolution [7], [8].

In this report, we described the presence of the STAT protein in a unicellular, free-living amoebae, *Acanthamoeba castellanii*. *A. castellanii* has been more thoroughly studied experimentally than most other free living amoebae. It has been used as a model organism for studies on the cytoskeleton, cell movement, gene regulation and mitochondrial bioenergetics [9], [10], [11]. The role of STATs previously found in slime molds has been assigned mostly to the development of multicellular structures that are facultatively formed by *D. discoideum* cells [12]. Therefore, *A. castellanii* seems to be a unique unicellular organism with a simple life cycle that possesses STAT proteins. We also described the results of a careful search among the STAT protein coding sequences of other lower eukaryotes. Using the constructed consensus sequences, we were able to find homologous sequences among various slime mold species but not in other Amoebozoa. Therefore, we concluded that *A. castellanii* is the only known Amoebozoa that does not belong to Mycetozoa but possesses STATs. To date, the presence of STAT proteins in other amoebae has not been investigated. We also showed the evolutionary relationships between the STAT proteins found in these simple organisms and their homologues from 19 Opistokonts.

## Materials and Methods

### Cell culture

The soil amoeba *Acanthamoeba castellanii*, avirulent strain Neff (ATCC30010), was cultured as described previously [13]. Briefly, the amoebae were cultured in a medium containing: 1.5% proteoso-peptone (Difco), 0.3% yeast extract (Difco), 0.5 mM MgSO_4_, 0.03 mM FeSO_4_, 0.03 mM CaCl_2_, 2 mM KH_2_PO_4_, 1.5% glucose, 83 µg/L vitamin B_12_, 33 mg/L vitamin B_1_ and 6.6 mg/L vitamin H, at 28°C with a constant agitation. After approximately 60 h of exponential growth with a generation time of 7–8 h, amoeba cultures reached the stationary phase with a density of 10×10^6^ cells/ml. Trophozoites of the amoeba were collected 24 h following inoculation at the early exponential phase (at a density of approximately 2-3×10^6^ cells/ml).

### Cloning and Sequencing of A. castellanii STAT cDNA

The *A. castellanii* total RNA was isolated using TRI Reagent (Sigma-Aldrich) with the modified single step method described by Chomczynsky [14]. The generation of RACE ready cDNA, followed by 5′ and 3′RACE and nested RACE (where needed) reactions was performed using the SMARTer RACE cDNA Amplification kit (Clontech Technologies, Inc.) according to the manufacturer's instructions. One µg of RNA was used to generate cDNA using SMARTScribe Reverse Transcriptase (Clontech Technologies, Inc.) oligo (dT) primer. During the reverse transcription reaction, the 5′ end of cDNA sequence was extended with SMARTerIIA Oligonucleotide (5′-AAGCAGTGGTATCAACGCAGAGTACXXXXX-3′), to facilitate 5′RACE reaction. The Advantage 2 Polymerase (Clontech Technologies, Inc.) and the following custom generated primers were used for RACE reactions:

5′-AGTACCTCAACCAGACCTTCTTCG-3′ and 5′-CCTCATCTACGGATTCTTGACG-3′ (3′ RACE and nested 3′ RACE), 5′-GGAAGTCCGGCAGAGTCTTCTTG-3′ and

5′-CCCTCGTCAAGAATCCGTAGATG-3′ (5′ RACE). The primers were designed using 540-bp long *A. castellanii* EST ACE00007771 available in the Database of Expressed Sequence Tags (dbEST). The predicted protein sequence coded by this EST showed a significant homology to known STAT proteins from *D. discoideum*. The RACE reactions were performed for 25 cycles at 94°C (30 s), 55°C (30 s), 72°C (3 min) (3′RACE and nested 3′RACE) and at 95°C (1 min), 56°C (30 s) and 68°C (3 min) (5′RACE).

The amplified fragments were subsequently cloned using standard methods and the pTZ57RT vector (Thermo Scientific) or the TOPO-XL Clonning Kit (Life Technologies), and then were sequenced.

### Databases, Multiple Sequence Alignment and Consensus Sequence

The search for nucleotide similarity was performed using BLAST (BLASTn) with the default search parameters [15], [16] and Nucleotide collection (nt) database (GenBank+EMBL+DDBJ+PDB+RefSeq sequences). The obtained *A. castelanii* STAT cDNA sequence was analyzed using EMBOSS Transeq software to obtain the corresponding peptide sequence [17]. The domain architecture was predicted and confirmed using SUPERFAMILY [18], SMART [19], Pfam [20] and CCD [21]. Other proteins that showed significant identity/similarity scores to the *A. castellanii* STAT protein sequence were subsequently found from a non-redundant protein sequence database at NCBI (all non-redundant GenBank CDS translations+PDB+SwissProt+PIR+PRF excluding environmental samples from Whole Genome Shotgun projects) using BLAST (BLASTp) with the default search values, unless otherwise stated [15], [16]. The preliminary multiple sequence alignments were achieved with the aid of ClustalX [22], [23]. Then, the alignments were verified and corrected by manual analysis using the algorithm of genetic semihomology [24], [25]. The consensus sequence of the aligned lower eukaryote STAT proteins was constructed with the aid of Consensus Constructor [26].

### Phylogenetic Trees

The phylogenetic trees were constructed on the basis of various algorithms of distance calculation with the aid of several independent applications. The programs used for the cladogram and/or phylogram construction were: ClustalX [22], [23], SSSSg [27], Phylip [28] and MEGA [29]. The phylogenetic trees were constructed on the basis of the aligned complete sequences.

### Availability of Original Software Generated by Authors and Co-workers

The original applications, such as Consensus Constructor and SSSSg, are freely available at the following addresses:

http://atama.wnb.uz.zgora.pl/~jleluk/software/wlasne/ssssg/ConsConstr.zip and

http://atama.wnb.uz.zgora.pl/~jleluk/software/wlasne/ssssg/ssssg.zip. The applications are also available directly upon requests sent to the authors. Additionally, the authors are willing to assist in the appropriate effective running of all of these applications in case of any problems.

## Results and Discussion

### A. castellanii *STAT Sequencing*

The *A. castellanii STAT* DNA sequence was assembled after amplification of 5′ and 3′ fragments using RACE and cDNA from cells from the early exponential phase of growth. The sequence of 2120 nucleotides included start ATG and stop TGA codons and the 3′UTR. An identical sequence has recently been published by Clarke M et al. [30] and is available in databases with the accession XM_004336169. The similarity search using this nucleotide sequence showed a homology with fragments of two other *A. castellanii* sequences (67% and 66% identity), which are also predicted to be STAT proteins (XM_004352905 and XM_004339187) [30]. However, the query coverage is only 25% and 40%. This homology is located in the C-terminal section of the coding sequence and includes residues 1356–1887. Interestingly, there are no sequences in Nucleotide collection (nt) database, from any other organism, significantly similar to *A. castellanii* STAT nucleotide sequence (the only similarity is found within short polyCAG tracts, the query coverage value does not exceed 10%). The N-terminal section of the coding sequence (first 522 nucleotides) is rich in CAG trinucleotide repeats. Recently, it has been reported that a large number of variable length triplet nucleotide repeats are characteristic for coding sequences from the slime mold *D. discoideum* [31]. It must be emphasized that trinucleotide repeats have not been previously described in the coding sequences of other *A. castellanii* genes.

**Figure 1:**
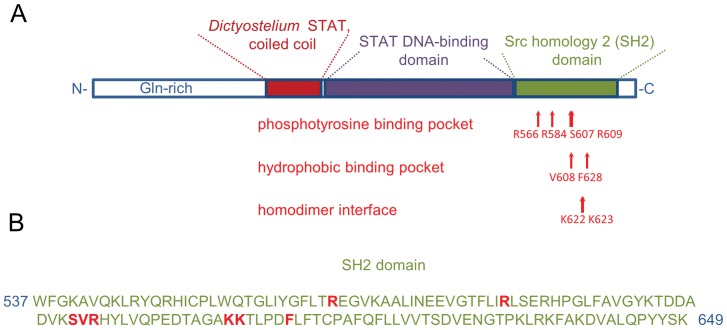
*A. castellanii* STAT protein domains. (*A*) The overview of the protein domains. The residues that form phosphotyrosine binding pocket, hydrophobic binding pocket and homodimer binding interface are indicated with arrows. (*B*) The SH2 domain sequence. The residues that form the phosphotyrosine binding pocket, hydrophobic binding pocket and homodimer binding interface are in red color.

**Figure 2:**
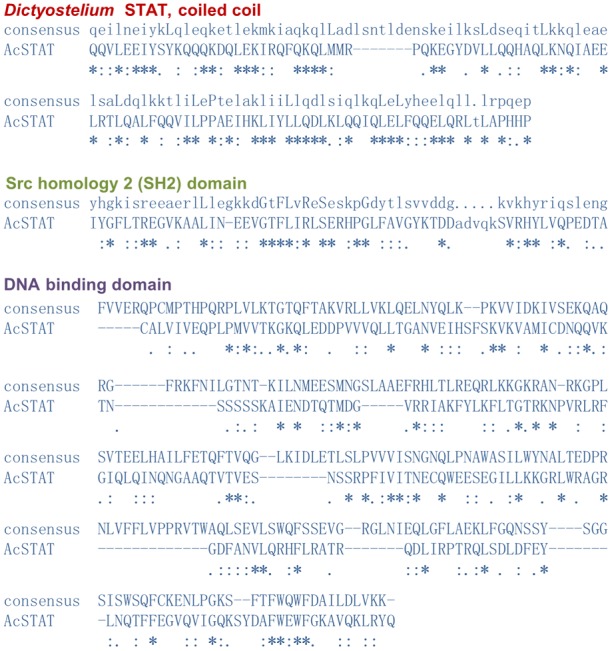
Alignment of STAT consensus sequences of the structural elements with the *A. castellanii* STAT functional domains. “*”, positions with a single, fully conserved residue; “:”, conservation between groups of strongly similar properties; “.”, conservation between groups of weakly similar properties.

The predicted protein sequence of the *A. castellanii* STAT protein is 689 residue long, with a predicted molecular weight of 77 kDa. The similarity search revealed that this is a STAT protein that resembles the STAT proteins from slime molds. The sequence includes domains characteristic of the *Dictyostelium* STAT proteins (Figure 1A). The predicted protein structure (using the SUPERFAMILY database) in the region between amino acids 220 and 325 forms a coiled coil similar to that of the *Dictyostelium* STAT coiled coil (E-value 2.3e-18). The region between residues 332–548 is predicted to be a STAT DNA-binding domain (E-value 2.53e-06). The C-terminal fragment of the protein (amino acids 551–668) includes the Src-homology domain (SH2) found in the STAT family (E-value 6.82e-21). This SH2 domain includes polypeptide binding fragments: phosphotyrosine binding pocket (residues 566, 584, 607 and 609), hydrophobic binding pocket (residues 608 and 628) and homodimer interface (residues 622 and 623) (Figure 1B). At the C-terminus (residue 677), *A. castellanii* STAT possesses the conservative Y residue that is phosphorylated during STAT activation. The alignment of the *A. castellanii* STAT fragments with the consensus sequence for corresponding structural elements are shown in Figure 2. The N-terminal domain of the protein contains a 220-residue fragment that is unrelated in sequence to any other proteins. This domain has little predicted secondary structure and contains long runs of poly(Gln) residues that are encoded by poly(CAG) tracts. Similar to the *Dictyostelium* STAT proteins [32], the protein from *A. castellanii* does not contain a STAT protein transactivation domain or an N-terminal α-helical domain. Both of these fragments are important for mammalian STAT protein function and play a role in binding coactivators, STAT activation, deactivation, and the stabilization of interactions between STAT dimers on adjacent DNA binding sites [33].

### STAT Protein Phylogenetic Relationship among Amoebozoa

The search for the sequences homologous to the *A. castellanii* STAT protein revealed 17 additional protein sequences from lower eukaryotes (BLASTp search). Interestingly, all of found (with expect value threshold set to 1) sequences come from Amoebozoa organisms that belong to either Mycetozoa (slime molds *D. discoideum*, *D. fasciculatum*, *D. purpureum* and *Polysphondylium pallidum*) or Centramoebida (*A. castellanii*) (Table 1). All of these sequences show a high level of identity (from 56 to 68%) with a query coverage range of from 31–70%. Moreover, no similar sequences were found in other unicellular organisms (BLASTp search); therefore, *A. castellanii* remains the only studied unicellular organism with a simple life cycle and STAT. Based on the data from the Origins of Multicellularity project [34], the only exception could be the parasitic filasterean *Capsaspora owczarzaki*, which is recognized to be a close relative of metazoans. The sequence from *C. owczarzaki* was included in the set of sequences used in rest of the study. The multiple sequence alignment of 18 sequences and the consensus sequence construction revealed conserved protein regions among these organisms (Figure S1). The consensus sequence was constructed at the threshold parameters of identity (66.67%), moderate conservativity (27.78%) and gaps (50.00%). It showed significant features of the lower eukaryote STAT proteins and served as a query sequence for an effective search of the evolutionary and functionally related proteins.

**Table 1:** Protein sequences similar to A. castellanii STAT (accession number XM_004336169).

Accession number	Organism	Identity [%]	Positives [%]	Query coverage [%]	E value
XP_004355663	*Dictyostelium fasciculatum*	52.45	68.44	70	5,00E-161
EFA83377	*Polysphondylium pallidum*	53.70	68.08	68	3,00E-160
XP_003295153	*Dictyostelium purpureum*	52.35	68.38	67	9,00E-160
CAC33514	*Dictyostelium discoideum*	52.56	68.38	67	3,00E-157
EFA82265	*Polysphondylium pallidum*	43.51	58.58	67	2,00E-109
XP_003291486	*Dictyostelium purpureum*	43.62	58.72	67	3,00E-106
XP_640661	*Dictyostelium discoideum*	42.53	57.47	67	4,00E-104
XP_004366323	*Dictyostelium fasciculatum*	42.02	58.61	66	5,00E-104
XP_646834	*Dictyostelium discoideum*	39.43	56.98	66	5,00E-103
XP_004360534	*Dictyostelium fasciculatum*	36.27	50.18	65	2,00E-93
XP_004339235	*Acanthamoeba castellanii*	43.41	58.86	65	1,00E-91
XP_004338884	*Acanthamoeba castellanii*	35.32	50.64	62	7,00E-70
XP_003283502	*Dictyostelium purpureum*	32.40	49.14	61	1,00E-52
EFA77913	*Polysphondylium pallidum*	31.65	51.38	61	6,00E-51
XP_643781	*Dictyostelium discoideum*	32.78	49.28	58	3,00E-48
XP_004362589	*Dictyostelium fasciculatum*	32.37	48.55	58	2,00E-44
EFA75761	*Polysphondylium pallidum*	38.49	56.49	31	5,00E-31

**Figure 3:**
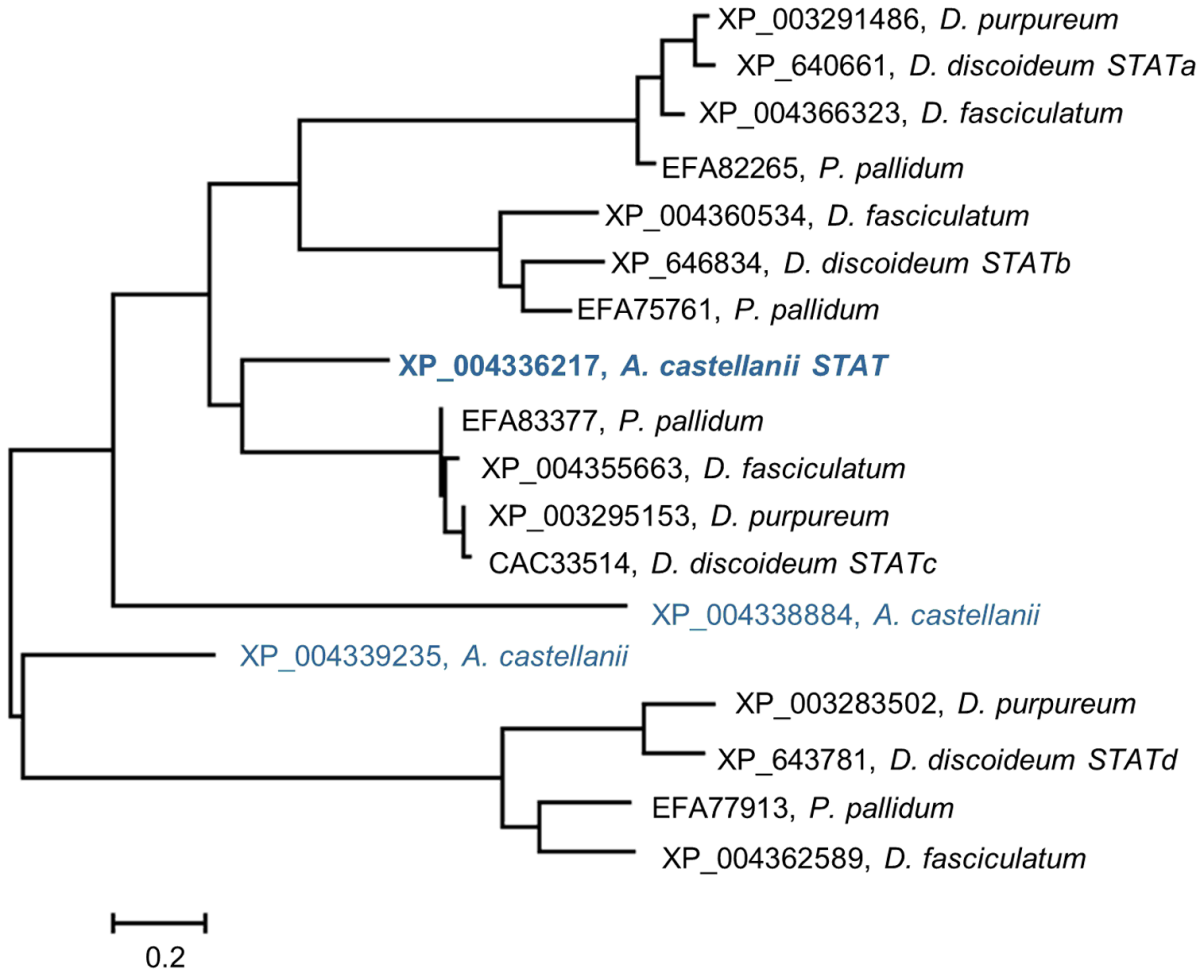
Phylogenetic relationship of the Amoebozoa STAT proteins. The results were obtained with the aid of MEGA 5.

As expected, the N-terminal fragment shows a very high variability and no significant identity/similarity among the compared organisms. However, the rest of the sequences show a significant degree of conservativity/similarity (especially the C-terminal part), which is sufficient proof that they are homologous and that phylograms may be constructed for them. It is obvious that similarities are predominant in structurally important regions, i.e., in domains of a putative *Dictyostelium* coiled coil, the STAT DNA-binding domain, the linker domain that contains an EF-hand motif and the SH2 domain. All of these proteins have a conservative Y residue (number 1321 in the alignment, Figure S1). Previous studies show that *A. castellanii* SH2-containing proteins generally have unique domain combinations that are similar to those found in *Dictyostelium* [30]. The construction of phylogenetic trees using lower eukaryotic sequences and various tools led to the generation of various trees with respect to tree topology and branch distance length, depending on the applied method of analysis (Figures 3 and S2). It has been shown previously that it is favorable to use several methods of phylogenetic analysis for better reliability [35]. However, all of these methods indicate that there are four separated clades within the slime mold STAT proteins. All trees grouped the studied sequences from *D. discoideum*, *D. fasciculatu*m, *D. purpureum* and *P. pallidum* into four groups. Moreover, all of these organisms seem to have exactly four STAT proteins that show a very close evolutionary relationship to one of the *D. discoideum* STATs (STATa, STATb, STATc or STATd). Only a STATb analog has not yet been found in *D. purpureum*. These observations suggest that the Dictyostelids' STAT repertoire was completed before the speciation of these slime mold species. The *A. castellanii* STAT protein branches next to a group of STATc proteins from slime molds. The other two sequences from *A. castellanii* seem to be more distantly related to the slime mold clades. It is especially interesting that no STATs have been found in any of the other Mycetozoa groups or in any other species that belongs to Amoebozoa. The open question remains what is the role of STAT proteins in *A. castellanii*. It has been previously shown that in STAT proteins in *D. discoideum* play a role in several aspects of differentiation. Extracellular cAMP signaling activates STATa, which can function as either a repressor or an activator of specific gene expressions [36]. At the *D. discoideum* slug stage, STATc in one of the prestalk cell subtypes, pstO cells, that prevents the expression of a marker of pstA cell differentiation [37]. However, it has also been reported that STATc accumulates in the nucleus rapidly when slime mold cells are subjected to hyperosmotic stress [38]. Therefore, STATc seems to be a key regulator of the transcriptional response to hyperosmotic shock [39]. Approximately 20% of the differentially regulated genes in *D. discoideum* cells treated with 200 mM sorbitol were dependent on the presence of STATc. The stress response could possibly be the answer to the question of why the simple unicellular amoeba *A. castellanii* needs STAT signaling.

### Phylogenetic Position of Amoebozoa STATs

**Figure 4:**
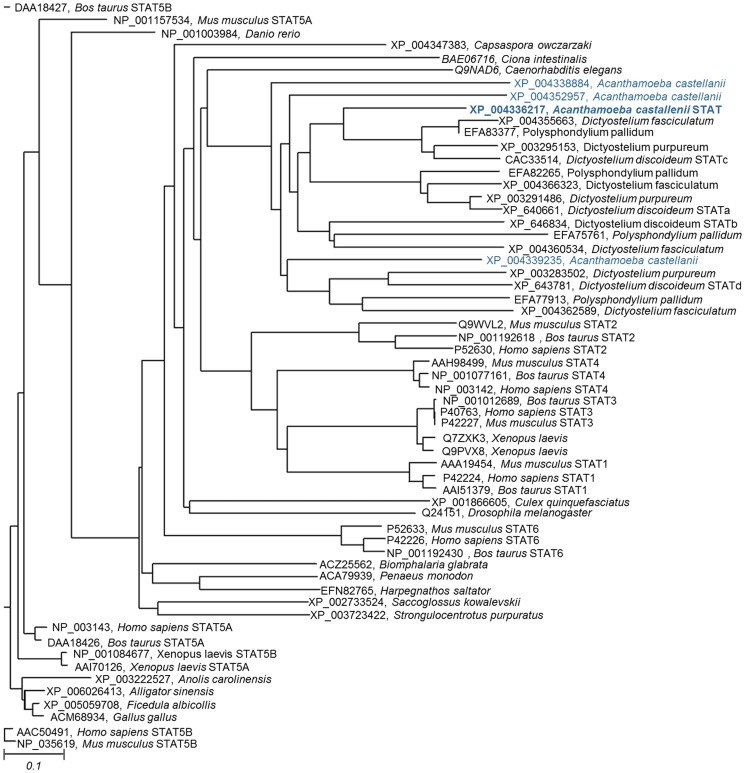
Molecular phylogenetic analysis by Maximum Likelihood method of 59 STAT proteins from selected Ophistokonta and Amoebozoa. The results were obtained using Phylip.

To further investigate the position of the *A. castellanii* STAT among the other STAT proteins, analyses were performed on a second dataset. This dataset included the STAT protein sequences of eukaryotes, including the *A. castellanii* STAT, Amoebozoa STATs and 19 other eukaryotes that represent the most sequenced lineages from Opisthokonta. These sequences are from roundworm (*Caenorhabditis elegans*), fruit fly (*Drosophila melanogaster*), southern house mosquito (*Culex quinquefasciatus*), acorn worm (*Saccoglossus kowalevskii*), sea urchin (*Strongylocentrotus purpuratus*), filasterean (*Capsaspora owczarzaki*), sea squirt (*Ciona intestinalis*), zebrafish (*Danio rerio*), freshwater snail (*Biomphalaria glabrata*), Jerdon's jumping ant (*Harpegnathos saltator*), giant tiger prawn (*Penaeus monodon*), African clawed frog (*Xenopus laevis*), Chinese alligator (*Alligator sinensis*), chicken (*Gallus gallus*), Carolina anole (*Anolis carolinensis*), Collared Flycatcher (*Ficedula albicollis*), cattle (*Bos taurus*), house mouse (*Mus musculus*) and human (*Homo sapiens*). We included some closely related organisms, e.g., *G. gallus* and *F. albicollis*, to access the variation within closely related species. The assembly of the STAT protein sequences generated a multiple sequence alignment and phylograms. The multiple sequence alignment is shown in Figure S3. The maximum likelihood analyses results of these sequences using various tools are shown in Figures 4 and S4. Regardless of the algorithm used, all of these studies showed that Amoebozoa form a distinct monophyletic lineage within the STAT protein world that is well separated from the other groups. *A. castellanii* sequences XP_004338884 and XP_004339235 occupy basal positions in the clade. It has been previously found that the only SH2 domain-containing proteins from Amoebozoa that have identifiable orthologs in Opisthokonta are the transcriptional regulators Spt6 and STATs [40]. Interestingly, there is no evidence of STAT signaling in Fungi. This observation suggests that the STAT proteins in Amoebozoa and Opisthokonta are a product of convergent evolution, represent examples of horizontal gene transfer from the choanoflagellate line or they were lost in Fungi. Given the conserved STAT protein sequence and architecture, the last hypothesis seems to be the most likely [7].

In conclusion, the study of the *A. castellanii* STAT, which is the only STAT protein known in a free living unicellular organism, provides a view into the evolution of SH2 domain-mediated signaling and the development of multicellularity. Indeed, we are far from understanding the significance of the STAT signaling pathways within early eukaryotes. Further studies on STAT protein are necessary to better understand of its role in the simple life cycle of *A. castellanii*.

## Supporting Information

Figure S1 **Multiple sequence alignment and consensus sequence construction of the Amoebozoa STAT protein sequences.** (*A*) The multiple sequence alignment for 18 lower eukaryote STAT sequences is shown. Sequences are identified by accession numbers (for details see Table 1). Black background - high conservativity (the same amino acid residue in 66.67% of the sequences), gray background - moderate conservativity (the same amino acid residue in 27.78% of the sequences), gap in consensus sequence (49.15% of the sequences do not have a position). The last row is the consensus sequence for a given set. (*B*) Consensus sequence.
(PDF)

Figure S2 **Phylogenetic relationship of the Amoebozoa STAT proteins.** The results were obtained with the aid of the following software: (A) Clustal(X), (B) Phylip (maximum likelihood), and (C) SSSSg.
(TIF)

Figure S3 **Multiple sequence alignment and consensus sequence construction of 59 STAT protein sequences.** The species names and STAT protein isoforms that correspond to a given accession number are shown in Figure 4. (*A*) The multiple sequence alignment for 59 STAT proteins is shown. Black background - high conservativity (the same amino acid residue in 69.49% of the sequences), gray background - moderate conservativity (the same amino acid residue in 28.81% of the sequences), gap in consensus sequence (50% of the sequences do not have a position). The last row is the consensus sequence for a given set. (*B*) Consensus sequence.
(PDF)

Figure S4 **Molecular phylogenetic analysis by Maximum Likelihood method of 59 STAT proteins from selected Ophistokonta and Amoebozoa.** The results were obtained with the aid of the following software: (A) Clustal(X) and (B) MEGA5.
(TIF)
