# Supplementary material for: Acanthamoeba castellanii STAT Protein
Source: PLoS One. 2014 Oct 22;9(10):e111345. doi: 10.1371/journal.pone.0111345 (PMC4206453; doi:10.1371/journal.pone.0111345)
Supplement: Figure S1 — Multiple sequence alignment and consensus sequence construction of the Amoebozoa STAT protein sequences. (A) The multiple sequence alignment for 18 lower eukaryote STAT sequences is shown. Sequences are identified by accession numbers (for details see Table 1). Black background - high conservativity (the same amino acid residue in 66.67% of the sequences), gray background - moderate conservativity (the same amino acid residue in 27.78% of the sequences), gap in consensus sequence (49.15% of the sequences do not have a position). The last row is the consensus sequence for a given set. (B) Consensus sequence. (PDF) [file pone.0111345.s001.pdf]

Figure S1.

A

```
                               10        20        30        40        50        60        70        80        90       100
XP_003291486      ----------------------------------------------------------------------------------------------------
XP_640661         ----------------------------------------------------------------------------------------------------
XP_004366323      ----------------------------------------------------------------------------------------------------
EFA82265          ----------------------------------------------------------------------------------------------------
XP_003295153      -------------------------------------------------------------------------------------------------MSN
CAC33514          ---------------------------------------------------------------------------------------------MS--NNN
EFA83377          ---------------------------------------------------------------------------------------------------M
XP_004355663      --------------------------------------------------------------------------------------------MNY--CNS
XP_004336217      ----------------------------------------------------------------------------------------------------
XP_003283502      -------------------------------------------------------------------------------------------MS-----NN
XP_643781         -----------------------------------------------------------------------------MSQREWTTASTSDLMSNL--LNN
EFA77913          -------------------------------------------------------------------------------------------MDGS--QQQ
XP_004362589      -------------------------------------------------------------------------------------------MDQL--NKQ
XP_004360534      MSNINGG------FVSSYSSSSSSSSSSSPSLPTIPNFHQHNNNKSIAELSPPKSSTNINIEEYHHIQQTGQLQQKTMIPSVTKLKSQSDLMDISYSASL
XP_646834         MEVTNNGSNNSSTIASTNPPTSPSTTSTSKSLPPLSFLNSQWENK---QSNNNNNSNNNNINNNNNNNNNNNNNNNNNNNNNNNSVKMEKLIELP-SSSN
XP_004339235      ----------------------------------------------------------------------------------------------------
EFA75761          ----------------------------------------------------------------------------------------------------
XP_004338884      -------------------------------------------------------------------------------------------------MSS

                  --------------------------------------------------------------------------------------------------XX


                              110       120       130       140       150       160       170       180       190       200
XP_003291486      ----------------------------------------------------------------------------------------------------
XP_640661         ----------------------------------------------------------------------------------------------------
XP_004366323      ----------------------------------------------------------------------------------------------------
EFA82265          ----------------------------------------------------------------------------------------------------
XP_003295153      PKKRPLDAIN---GGFDVKQEQD--------------SSDFPSDGFN--------TTNDDLMSLMTFLDNGSGQNQNQNQNQNQNQ--------------
CAC33514          PKKRPLDAIS---NTFEVKQEE----------------PEFSSDGFN--------TTNDDLMSLMTFLDNGTGQQNQQNQQNQQPQPQPQPQPQLPQPQS
EFA83377          SKKRTLESLQDESGGFPEVKAEA--------------VDDLSADGF---------TTNDDLMSLMTFLDNGSSQSNFMVKPEQSLNQTAQLYTNIQQPQQ
XP_004355663      PHTPTTNTLIH--HTNIVGSKKP--------------LPEVININNN--------NNNNMLASSYITKEAAANKSPMLDPSPSSSSSSSSTTPPLLLTPN
XP_004336217      ----------------------------------------------------------------------------------------------------
XP_003283502      WESFN-NNNNNNNNQNQNQNQNQNQN--------NGNIPRGGSIFFN--------QDSTVDNS---YKASLTIDFPRVDSFGFLRSPTSVLSNTPIPNTT
XP_643781         HNSNNSNNNNNNNNNNNNNNNNNNDN--------NNNNNNNNNNNNN--------NNNNSNNQPLLRNASTNIFFG--DDW--TKQNTVGLLLDPLNRGT
EFA77913          SSANNLNGGNGSAGNGGDTQQHGTTQ--------DKEWMSTSSDVM-----------MNSFFGTGLHQPQVSNLGLSGNTFPSTLTKQDTVLNTFFNQDV
XP_004362589      LQEQ-LQQQQQQLQQGGQQQQQ--QN--------NGNWMSTSSDVLNYFDSN---NNNNTMITPLTKQDTISNMLNAGLTGAGTTSLLPNGGD-APNNSL
XP_004360534      PTPTLINPTTTNNNNNVNNTSKIHPNSLPSLNSIDWNYNNNNNINNGQQQQQQLLNSNNSSITNSPTNHHGSMKQMTSSMVLVTEGSGAGAGAPSSPENE
XP_646834         NSPEFLNGVNNNNNGNNNNNNNNNNN----------NNNNNNNNNNN--------NNNNNNNNNNNNNNSGSGGNNSNNNSNISSPPSSSSTSTSVLSSS
XP_004339235      ----------------------------------------------------------------------------------------------------
EFA75761          MSNNILNTSNSNTHQHLQHLLHS--------------SNSSGSSSSG--------SNSNSNNTRFTTNNQQQQQLLFEQIQQQSKMIPNSSSSSTLSKKS
XP_004338884      TRKHKAADGDEGEGSSPSSSPRSSPHR--------AKSPSSSSGNKK--------RRLDTNGSDESTATTSSSSSSTSTSSSVFPHPALHQLPPHLLNHS

                  XXXXXLNXXNX--XXXXXXXXXX--------------XXXXXSXXXN--------XXNNXXXSXXXXXXXXXSXXXXXXXXXXXXXXXXXXXXXXXXXXXX
```

```
                          210       220       230       240       250       260       270       280       290       300
XP_003291486     ----------------------------------------------------------------------------------------------------
XP_640661        --------------------------------MSSAEFSMDDFEDTFDSN--------------ATISTKDLFEGSDRLPLNQSINTTIQNLYLP-----
XP_004366323     --------------------------------MAS-EFPMEDYDD-FDS---------------SSLTTKNLFEGGSADKYHDSINTVMQMGYN------
EFA82265         --------------------------------MSS-DFQMDTDFEDFDS---------------SNLTTKNLFEG--AEVLNESINTTLNNLQYNNNN--
XP_003295153     ---------------------------------------NQNQNQNQNQN--------------QNQNQTQQLYTQQPQQTTIAIKTEGLSVNSATQN--
CAC33514         QQPIYNSNTTTVTVKTEGIATSPLSNASSPISTNNNIYNNTNNNNNNNNN--------------NNNNNNNNNNNNNNNNNTATPPAIGVQQNSNIP---
EFA83377         QQ---------------------------------Q---QQQQQQQQQQQ--------------QQQPQLQNIYTNNGQQVYQQQPQNGQQQQMISPP--
XP_004355663     ILLIPN------------------------------LMNNCNNSNNNNTN--------------LYNHHHNHINSHGQNITTIKTEPVSTLQYPQQPQ--
XP_004336217     ---------------------------------------MQGGGPAYPGG--------------GIKMETGQQQPAPLLHAPPNSAPSFLYHHPPSPF--
XP_003283502     GLSDIG---------------------------LNAQYYQLIDSQLPHDE--------------QVQK-LQKFHMEQQENLQRQQNQQLQSLQLSNGSL-
XP_643781        SFSNL----------------------------LKSP-TILGDTQQSQQP--------------QSQHTMQQLFANDPTYQQHQQEQEQQHQMMGN----
EFA77913         EWSNN----------------------------VSNQSNFFMQNSLSNAG--------------HQQSFGSLLQSIHPNVNSSTASTTTTTTTTTTSG--
XP_004362589     DWASG----------------------------RTNSFSSFSFLRTSNIM--------------SNDTTTSIINTHNQPLQQQIFNPYINNTSTNNNN--
XP_004360534     HSAMEFKSF--------DRKVASAIDSFGKFESLQKQYESQMGQIMSQME----REQKQRAQ---IEDTCTTLQNENLKLRQDHKSINKKLKELEKSNE-
XP_646834        QSAYMN-----------DKMVAATLDSIGKMESIQRKYEMQIESLMDQIQGYIEKEQKLRSQCQAVEDINAKLENENLQLKKELFEMSRKFKEIDIINLN
XP_004339235     ----------------------------------------------------------------------------------------------------
EFA75761         SLSHMTN-----------------------IDDAASAFAVHSGGSGGGGG----------GSSGGIGSPSSILKIHSNSNNNNSNNNNVSLPSLSDIN--
XP_004338884     HPGGDLG-----------------------ESLRFSQVARLLDASAEDGG-------------LGLGHSSSGLGTSTDSSFWKMLETEDPSEVTKQMN--

                 XXXXX----------------------------XXXXXXXXXXXXXXXXX--------------XXXXTXXXLXXXXXXXXXXXXNTXXXXXXXXXXN--

                          310       320       330       340       350       360       370       380       390       400
XP_003291486     ----------------------------------------------------------------------------------------------------
XP_640661        ---------NGGFAIGDQ----SQQQYYQAMPP----LNQSDQFNLGRSNNL-----TPRTNQLQQLQQQQQQQQQPQQQ--------------------
XP_004366323     ---------TGGFGITQS----VNGTFYSNQP-----VDYRPVMNGGQPTYV-----APNS---------------------------------------
EFA82265         ---------GNGFGMGAIDHASQQQQYYQQMHS----PQQIDYRGTPRGGSI-----PSQS----QYLQHS-----------------------------
XP_003295153     ---------LYQYPLYTQEVN-------QNIGQT---VPIEPFFQ-IDGAQI-----QQQ-LLQQQLQPIQTS---------------------------
CAC33514         ----------YSYPIYTDVTGQQTQHQ-QNIGQNS--VNIDPYFQTIDGAQI-----QQQQLLQQQLQPIQQVNNPQIDQAQIQQQQAQQIQQQQAQIQA
EFA83377         ----------QQPTYYTNGTNIDDAHK-LQQQQMM--LQAQAQAQAQQQAQV-----QAQQQAQAQAQAQAQQQVMTS----------------------
XP_004355663     ---------QQQPQQQQQQVQQVQQQQQQPQSQYI--HPPPAYIYNGNGGTL-----EDQQQQQQQLQQQHQIQQQQ-----------------------
XP_004336217     ---------GLHPATTPGGVAFGQQQQQQQQTHVS--MGVAPGGGVASPLQH-----QQQQLHQQQQQQQQGVPMHAMLG--------------------
XP_003283502     ---QYQTTTTTTTTAAQPLLN-EQQFAFQHINNLPSVFNSNPTITT---NNP-----MTANLHNPYLNAPQSYTSKMN----------------------
XP_643781        ---------TTSPKSFNPYVGLSQPMIVTNTGN----FNTTTTTTTTSNNNN-----NNNNNNNNSNNNNNNNSMNIN----------------------
EFA77913         -----GNSTLGQSNPYTNVNYQFQQHQHQQQNQQ---QQQYNTSSSSPMNT--------TNDDDEIVATPPTPTIIDH----------------------
XP_004362589     -----NNIHMGDLSTHNPLHHLTSTTSTTSTNNG---LNTSVTMSSSQQSSP-----MKEDDYEDQPATPPTPPIHTP----------------------
XP_004360534     YLMMMLQQKINTPILQQTTSPPPPPSSLVQQQQPPPQLTFTPPSNSFSPIPN----NNNNNIIQKSPSPSN-ISGPHPLNDKSILLLKKKKK-----GD-
XP_646834        NTNNNINNNNNNNNNNNNNNNNNSINNNNNNGFSPPPLVKYPSNGSLQQDAKRFKIMEQQSQQQQQMQQQQQIQQQKQYQQQQQQTTSKRKNNISIDGDK
XP_004339235     ----------------------------------------------------------------------------------------------------
EFA75761         ----WNNNSNSNPNINNSSANNNHHHHNHNHHHN---HSHTHHSQSSQNSNN----INNSSGGVSPLTNSHQSDYSSNSN---------------------
XP_004338884     -----QTAELQQQLLKTAHLLRSSQDGTEAAGGNHHHQDASLYASQGGGGHQ-----PQPSLSQSQLMSALMGSASAN----------------------

                 ---------XXXPXXXXXXXXXXQQXXXQNQXQX---XXXXPXXXXXXXXXX-----XXQXXXQQQLQXXQQXXXXXX----------------------

                          410       420       430       440       450       460       470       480       490       500
XP_003291486     ----------------------------------------------------------------------------------------------------
XP_640661        -Q-----QQQTYGT---QSPIHMSQTPSSPLSSPLP--------------------------------------------------------------SP
XP_004366323     ------------GP---HSPVNMQ--PSSPISSPLP--------------------------------------------------------------SP
EFA82265         ------------NG---GSPLHIPQSPSSPLMSPS---------------------------------------------------------------SP
```

```
XP_003295153     -------------------PTQIEINQQQQQQQP-----------------------------------------------------------------I
CAC33514         QQAQIQQQQLEQQH---LQQQQFQFQQQQQQQQQQQQQQQQQQQQQQQQQQQQQQQQHHQQQQQQQQHQQQQQHQQ--------QQQHQQHQQQQHQQHQ
EFA83377         -------------------PQQILMNPQDLQQQQQQ---------------------------------------------------------------N
XP_004355663     -----------------LLQQQILQQQQFQQQQQQQQQ-------------------------------------------------------------Q
XP_004336217     -----------VPS---PGGQQALSPPSTAAAFSSP---------------------------------------------------------------H
XP_003283502     ----------ANTY---DDDE-ELNTPPTPP--AVEWDSP-----------------------------------------------------------P
XP_643781        ----------ANTY---EDGEDEPKTPPTPSNEPINWDMPN--------------------------------------------------------YR
EFA77913         ----------DSSM---QQQQHQLNNPPPLVYQPSFG-RSA--------------------------------------------------------SA
XP_004362589     ----------PIHH---STPPISYGNSFLQAGDMNLMSSSV--------------------------------------------------------VG
XP_004360534     EELVAETLGSFVELTASRKPMLKRSNSEDSFMHSIPYHLN--GGVPGHPATSLNSNNNNVIPTTADGSSSLPMSG------------DYPFAQFGSLGHI
XP_646834        EALVAEALGSFVDY---AKPSLKRSNSEEVFNSSVYKNKNNINNINNNSNSNNNNGNNSLLNDIQNWQQQQQQQQQLLHQRKKRKDYDYDYNSTQNGKGI
XP_004339235     --------------------------------------------------------------------------------------------------ME
EFA75761         -----------SSS---SSSNNKLGNIMDVPSSPEFSHL-----------------------------------------------------------HQ
XP_004338884     ----------------ELLGEGVVDYPPLSASTHALLTS----------------------------------------------------------FIK

                 -----------XXX---XXPXXXLXNPXXXXXXPXXX-------------------------------------------------------------XX

                          510       520       530       540       550       560       570       580       590       600
XP_003291486     ----------------------------------------------------------------------------------------------------
XP_640661        TPFSRQQSY-------N-NNNSNNTSSSQNY------NNNNININNNNNNNNTNNNNNNNNGNNS-----NGNNGNNNNNNNNNNNNTNNNNNNNNQQQQ
XP_004366323     QSFRQSQG------------QLGQMPPQQQ--------QQPIMYQQPQQQMQPQQQHMMQQ--------------QPQYSMQPQQQQQQPQQQFQQQQQP
EFA82265         FNGRQSSG----------------GIPQQIP------PQSPQQIPQQHYPQQPQQQQYQQQ---------------------QYQQPQQ-QMHHQQPQQM
XP_003295153     QIHQPQT-----------------IQNIPQL-------QPIQTMQQIEQLPIIHQTISQPSL-----------VDQLQPIQTNN--NNNISLPSNEELQQ
CAC33514         QIHQNQLNHQNQLNNQNQLNNQNQLNNQNQLNHQNQLNQPTQPQMQQIELPILHQLPVEPQH-----------LHPIPTHINNN--GNNNNNNNNNGSNS
EFA83377         QIYQQQMG------------------------------QMLQSPPQQLQHIQIQSPPQQQTI-----------MHTSPPQILQS--PPQSSLQLPTPVPV
XP_004355663     QIQQHVQS------------------TMIQV-------QQQQQQVQQQQQQQVQQQLSSPNN-----------NNQESIIITTSGINGQQTLSIQQPQQQ
XP_004336217     QHHHQQLQ------------------------------QQQQQQQQQLLLQQQQQLQQQQQH-----------FASQTAHMQQLR-QAGGLLLVQSPATV
XP_003283502     Q---VFR-----------HPTTSFGSPIE---------YS-KNDNEGEQPTKYQRLTTENSV-------------ELSALNHKLSNDPHYSISFLREQTT
XP_643781        QNTASFHADININNTTNSHPTT-LSSMMKNEIP----TYSGQVSNEYEQPTKFQRISTYN---------------EES-LNDRLRGDNTGSINFLQAETS
EFA77913         LN--DPLSNLIQNDLGDAHHPTPNGEPNEYE------PPTKLARTATLQLQGMPATSTANYS----------DPYEEQISKV---ANLNVHLSFLRSQTE
XP_004362589     LMGQQTMSNIFQFG-GNNNNNNNNNNNNNNN------GQQNTGRSGEFQINEYESLTKMQRTNT--------QTYEAQINKLPVETNLDLQISLLRSHTE
XP_004360534     QEEQSKLKKRRSEFGNKDDPKKKSLSSLPLLTINNTNNNLHNSYHQQHQLQQQKSFNLLNNIKIE--QDQQQQGNSLKSSATILRKQQQQKLHIPDLETD
XP_646834        PSNSSNNNSSNNNSNNNNNNNSNNNNNIIGSISPPHSSQLQQVSSPQQQQQQQKPNGLKLSISSGSIKDLINSPNKEQSSKSQYPSSLSQSSSIPDMDTD
XP_004339235     ETDLDEMNPEFGNEFWKELTDDVLDTLYSQL-------EQPAFDTSEYYLDDDDDGMLGSTNPGG--FVLPEAAHGAMAPAVQVKDEDAVARTVDPAHFQ
EFA75761         QQQQQQHHRSTTLSSSSSSSSMVVDSANVRLQQYEASIESLINQVQSAHDREQKQRSYTKSIEEG--------YANLESENLKLKKELFEMNKKLNEILQ
XP_004338884     QEGASSSG------------GATIASVAGSG------VAALPLPPPPHGTIAPSPASAFSSP-----------TAAASSGKKSGGGHKATRSGSTSPAPQ

                 QXXQQQXX----------XXXXXXXSXXXXX------XQXXQXXXQQXQLQQXQQXXXXXXX-----------XXXXXXXXXXXXXXXXXXLXXXXXQTQ

                          610       620       630       640       650       660       670       680       690       700
XP_003291486     ---------------------------------------MPQ----QSQPQQ----------QAPQTQISSP---QPILDTI----YKLLSEQEQTLVQM
XP_640661        QQQQQQQ-------------------------------QQQQ----QQQQQQ----------QQGNPNLSSP---QPILDTI----YKLLSEQEQTLVQM
XP_004366323     QIVHQPI-------------------------------HQPP----PQPQQQ----------QPPSTAISSP---QPILDTI----YKLLSEQEQTLVQM
EFA82265         QPHQQQM-------------------------------HQQP----QQQQQQ----------QAPSTAISSP---QPILDTI----YKLLSEQETNLVKM
XP_003295153     MN--------------------------------------NQ----VQNQLHCN----NNSLPLPDHLLIN----TPYGN-V----LQPHQQIINECLKL
CAC33514         SNSSNGI-----------------G-------SPDDIIEPNI----LSSIQHCN----NNNLPLPDHLLIN----TPYGN-V----LQPHQQIINECLKL
EFA83377         IP--------------------------------------QQ----LQNQQP---------IPLPPHLLV-----VPFGNQP----LQPHQQIINECLKL
XP_004355663     MAPQP---------------------------------HQQQ----TLSIQPPQ----QN-IPLPQHLLV-----APFGNQA----PQPHQQIINECLKL
XP_004336217     VAAAP----------------------------------VAA----AAAAAP----------GGPAQLQVP----STLEG---------CQQVLEEIYSY
```

```
XP_003283502    KLKS------------------------------------------QASTDD---------LSRNKSFTIN-----HYETGV----DQISMEYYNNLLLR
XP_643781       KLRSR-----------------------------------------QSSQDD---------LERNRSFQMT-----HYDTGC----VPISTDYYNSLLTR
EFA77913        KLEDDV--------------------------------EKMR----QSSSQN---------LSQSSFLGGLN---KYAGTNT----EKISQEFYNNLINR
XP_004362589    RIEGDV--------------------------------ERLK----HSSNSN---------LSSHSFLQNQ----EYTGTNT----EKISQEFYNSLLNR
XP_004360534    VDTSDEIGDFS---QN----------NTPMKSSGGIIRNSGS---IPSENDDDP----RNGSSSPIEAPMS--DANLFKSIA-GTDDTPHEATLNEIHRL
XP_646834       VDSTDEF-DFGSNSNNNNNNNNNNNNNNNSNNSNNKKRNNSNNNNLGGDDDDSPDSNDRNGSSSPIDMEPSYDGANLFKTVTPGTITTPQEELLNEIHSL
XP_004339235    QQGQQ---------------------------------HQHH---QQAAAEPRQ-------QARPAAAHVP----LPAISQD----QAKARMVIDQVQQL
EFA75761        MKFSPQP-------------------------------TISA----PTSSSS---------SNLPITSKFTS---SLAVSGS---LPPPPSHADKKKLEL
XP_004338884    SSSSTSS-------------------------------GGGG----QAAAQH----------SAAAAAAAA----AVAGMND----GSAAAALLSQLASM

                XXXSXX--------------------------------XXXX----QSSQQX---------LSXPXXLXXS----XPXGXXX----XKPXQEXXNELLXL

                       710       720       730       740       750       760       770       780       790       800
XP_003291486    IHEQSILLNRLP----PNLDENSLVLL-----------------RQISQKQITLSSQMNTEMSALDATKKGMILEPTDLAKLFALKQDLQIQFKQLSLLH
XP_640661       IHEQSLLLNRLP----PTLDENSLAPL-----------------KSLSQKQITLSGQMNTEMSALDATKKGMILEPTDLAKLFALKQDLQIQFKQLSLLH
XP_004366323    IHEQSILLNRLP----QTLDETTVQHL-----------------SNLSQKQVTLSNQMNTEMSALDATKKGMILDPTDLAKLFALKQDLQIQFKQLHLLH
EFA82265        IHDQSILLNRIT----QPLDEQTIHSL-----------------QALSQQQVTLSSQMNTEMSALDATKKGMILDPTDLAKLFALRQDLQIQFKQLTLLH
XP_003295153    HLAQKEQLEKMKIVQKQVLAHPQKETF-----------------QMLDNEQNTLKKQIDAEITSLQQIDQTFVLSPPEIRNVIFLLHELTIQSIQLELYH
CAC33514        HLAQKEQLDKMKIVQKQVLAHPQKETF-----------------QMLDNEQNTLKKQIDAEITSLQQIDQTFVLSPPEIRNVIFLLHELTIQSIQLELYH
EFA83377        HLAQKDQLEKMKIVQKQVLAHPQKETF-----------------QMLDNEQNTLKKQIDAELTSLQQIDQTFVLSPTEIRNVVFLLHELTIQSIQLELYH
XP_004355663    HLAQKDQLEKMKVVQKQVLAHPQTETF-----------------QMLDNEQNTLKKQIDAELTSLQQIDQTFVLSPTEIRNVVFLIHELTIQSIQLELYH
XP_004336217    KQQQKDQLEKIRQFQKQLMMRPQKEGY-----------------DVLLQQHAQLKNQIAEELRTLQALFQQVILPPAEIHKLIYLLQDLKLQQIQLELFQ
XP_003283502    YNTRPENFERAKEKQIQVINGNNKQYS-----------------DELQTLYTTMKKDIDEENNELKNLLSKKILEPLDLRKIKETIEGLKSHLRVVEVLH
XP_643781       YNNRPEFFERAKENQIQVINNNNVQLA-----------------IDLQNIYTSMKKDIDEENNELKNLLTIRILEPYDLRKIRECLEGLKSHLRIVDVLY
EFA77913        YHERNERFLKAKEQQVTVITTHDIVLA-----------------NELHGVYTTMKKEIDEEKAELQNLLSKTILQPPDLHQIRKTIEGLQSHYRIVDVLY
XP_004362589    YHERNERFLKAKENQIKVIHNNDINLA-----------------RELQVVYTDMKKHIEEEKMELKNLLSKTILQPPDLHQIRVTIEGLKSHHRIVDVLY
XP_004360534    QLQQHEVLEKMALAQKQLLIEHHQILTGHIAPTPLNTARIEETYATLQVEQKKLHGQIDSEVTALNQMYSQTILEPNQLCKLDILNQELEIQMKQLQIYQ
XP_646834       QMQQRETIEKMYIAQKQFLSDRSNGFN--------NNN--EEILRSLQSDQTKLGSTLESELQALNQLYSQTILEPNQLCKLDILLQDVSIQLKQLHLYQ
XP_004339235    FATQDQHIEQIRQIQQQLVLQPQKEGV-----------------RQLQEQQWKLNQQIELELKELQGLHRSVILDPPALHQLRMLLQRLKIQQQKIELFR
EFA75761        QQKKKPRGGNGAIEGDEELVAEALGSFVELT-------------STSSSRKPMLKRSNSEDSFRHNPNQGMSMMSPHAMIMASRGGDPTSSSMDWAMGNS
XP_004338884    EESVDNQLKAMKFQQCDVIDTQLHNRA---------------EMDKLLKAQGDLRKQIEQSVQRLEQLNDTHILTATDAYRLVCLSDSFALHARRLRLYQ

                XHXQKEQLEKMKXXQKQVLXXPQKEXX-----------------XXLQXXQXTLKKQIDEEXXALQQLXXXXILEPTDLRKLXXLLQDLXIQXKQLELLH

                       810       820       830       840       850       860       870       880       890       900
XP_003291486    NEIQAILN--PQHSS-PK------PNVALVLKSQPFPIVISKGKQLGENQLVVLILTGARSNFHINGPVKATMICDS---------------------HP
XP_640661       NEIQSILN--PQHSA-PK------PNVALVLKSQPFPVVISKGKQLGENQLVVLVLTGARSNFHINGPVKATMICDS---------------------HP
XP_004366323    NEIQAVLN--PGSAS-SK------PNVALVLKTQPFPVVITKGKQLGENQLVVQILTGARSNFHINGPVKATLLCDS---------------------HP
EFA82265        HEIQSVLN--PTNQP-TK------PNIALVMKTQPFPVVITKGKQLSENQLVVQILTGARSNFHINGPVKATLMCDN---------------------HP
XP_003295153    EELQLLVR--PQNP--PP------TIAALVVIEQPFPMVITKCKPLEDDPVVVQLLSGTRTELQMIGKVRATMIVEN---QQ----------------G-
CAC33514        EELQLLVR--PQNP--PP------TIAALVVIEQPFPMVITKCKPLEDDPVVVQLLCGTRTELQMIGKVRATMIVEN---QQ----------------G-
EFA83377        EELQLLVR--PQSP--TP------TIAALVVIEQPFPMVITKCKPLEDDPVVVQLLSGTRTELQLIGKVKATMIVEN---QQ----------------S-
XP_004355663    EELQLLVR--PQNP--PP------TIAALVVIEQPFPMVITKCKPLEDDPVVVQLLSGTRTELQLIGKVRATMIVEN---QQ----------------N-
XP_004336217    QELQRLTLA-PHHPHLAR------PVCALVIVEQPLPMVVTKGKQLEDDPVVVQLLTGANVEIHSFSKVKVAMICDN---QQ----------------VK
XP_003283502    NELKYIIN--RKKP--E-------CCAALIITQQPCSQVIFRGGKGMADVFKVQMIFGVLQPDNISQISAVINKSET---TT----------------SK
XP_643781       NELKYVLN--RKKP--E-------CCAALIITQQPYSQVIFRGGKGMGEIFKVQMIFGVLQPDNISPVYATINKSESNPNST----------------TK
EFA77913        NELQYVLN--KKKP--E-------SCCAIILLQQPPSQVIFRGGKGIADPYNIELVTGVMMPDSISTVTAQIDKSES----A----------------TK
XP_004362589    TELQYILN--KKKP--E-------SCCAIILLSQPPSQVLFIK-KVIPDAYKVELVTGVLSPEHVSPVTAKLDKAES----N----------------SK
XP_004360534    LELSRINSP-GGGPEQTSASMSSSHTTALVITKQPFPMVISKFKQLQEDHLTVQLLTGSNSDIVWFSPIRAELTFHAKALTGPGGSGGSGSGSGASGSGT
```

```
XP_646834       MELN----Y-GYGSNEP-------FPATLVIIKQPFPMVISKFKQLQEDHLCVQLLTGANVEIVSYSPIRAELVFHSKNLTK-----------GSSNLGT
XP_004339235    QELQ-------LPT-----------------P-----QVLFKG-KTLEDNYVLALLSGSNVNIQNISKVKAILVAEE-----------------------
EFA75761        SDLDDLKFK-KRRSDQFMNSPPTEQPPAGTKTKKP-PQFNPSSPPNLQRHHSFSINSPVKLESSLHSSGGIMELSSSPSSSS----------NQLTSSGG
XP_004338884    EELFQAQSGQPVKP-----------VASLVITKQPFPCTVKQS-KSVDDPVDVVLITGAKSEIQAMGQVKAELINED--------------------YNP

                NELQXLLN--PQXP--PX------XXAALVIXXQPFPMVITKGKQLXEDPLVVQLLTGARSEXHIXGPVKATMIXES---XX---------------XX

                          910       920       930       940       950       960       970       980       990       1000
XP_003291486    -----------TNKNNPTTPLEMDSQPIYPATL-TAHFPLKFLAG--------TRKCSVNLKFG---VNIRDLDN---VTTTVESDASNPFVVITNECQW
XP_640661       -----------TNKNNPTTPLEMDSQPIYPATL-TAHFPLKFLAG--------TRKCSVNLKFG---VNIRDLDN---VTTTVESDASNPFVVITNECQW
XP_004366323    -----------TNKNNPQQVLEMDTQPIYPASL-TAHFPLKFLAG--------TRKSSVNLKFG---VNIRDMDS---VTTAVESDFSNPFVVITNECQW
EFA82265        -----------TNKNNPQQQLEMDSQPIYPATL-TAHFPLKFLAG--------TRKSSVNLKFG---VNVRDMDS---VTTAVESDSSNPFVVITNECQW
XP_003295153    -----------SKTSSSPKTIETEVVSMDETNR-LAKYHLKFLNG--------TRKNPVTLKFG---MQVQVVGG---TPVNIESPPSSPFIVITNECQY
CAC33514        -----------SKTSSSPKTIETEVVSMDETNR-LAKYHLKFLNG--------TRKNPVTLKFG---MQVQVVGG---TAVNIESPPTSPFIVITNECQY
EFA83377        -----------SKSSSSPKTIETEVVSMDEVQR-LAKYHLKFLNG--------TRKNPVTLKFG---MQVQVVGG---TPVNIESPPSSPFIVITNECQY
XP_004355663    -----------SKTSGSPKTIETEVVSMDEVQR-LAKYHLKFLNG--------TRKNPVTLKFG---MQVQVVGG---TPVNIESPPTSPFIVITNECQY
XP_004336217    -----------TNSSSSSKAIENDTQTMDGVRR-IAKFYLKFLTG--------TRKNPVRLRFG---IQLQINQNGAAQTVTVESNSSRPFIVITNECQW
XP_003283502    -----------KEKTTTVPTLENAESHLNTQTW-EAEFKNIKINVS-------TRMTPSSLRFI---ANYKEKTSGKTVEKQVESVPSNPVIVITNESQW
XP_643781       -----------KEKPTTTPTLENAEASLNTQTW-EAEFKNIKVNVS-------TRMTPSSLKFV---ATYKEKTSGKTVEKDVESVPGSPIIVITNESQW
EFA77913        -----------KEKSAATPSLENNECSLDKL---KATFTNLKVNIS-------TRMNPSHLKFT---ATVKDKGN--KASKSIESVQTNPIVVITNESQW
XP_004362589    -----------KEKSALP--IENYEIPFEKN---KATFTNLKINAS-------TRMTPTPLKFS---ATIRDKSN--RQGKPIESVPTNPILVITNESQW
XP_004360534    GKNSGANSQNVVQNNHLKKHIEKDTQVLDMSKG-TAKFPIKFLTG--------TRKGCVKLHFQ---LQARTTDG---HTFNIPSGSSQPFIVITNDCQW
XP_646834       -------------QNSLKKNIEKDTQVLDPIKG-VAKFPIKFLTG--------TRKSCVKLHFV---LQIKTSDG---HIINVPSSTSQPFIVITNDCQW
XP_004339235    ------------KNWKNKKPIENDVQAMDSMKR-VLTFHNIKLNVS-------TRMSMVYLKFA---VQVTLQNG---GTHTIESAGSSPIIVITNESQW
EFA75761        ----ILKKPKKQNNNNNNNNIQTTTTTSTSTPQQQSHFPSQMQNIGQLPEMETDVDSTDEFDFKGSTGMPRSGDGDSPDSNENSRNSSSPIEAPMSDANL
XP_004338884    ----------ISKKKNSAPAIQNAAETMDESLM-VTFKRLIFPHG--------SRVKSVNMRFA----QEVTMNG---SSMRLLSEPTKPFIVMTNHGQR

                -----------XNKNNSXKTIENDXQSMDXXXR-XAKFPLKFLNG--------TRKNPVXLKFG---XQVRDXDG---VTXNVESXPSNPFIVITNECQW

                          1010      1020      1030      1040      1050      1060      1070      1080      1090      1100
XP_003291486    EGS------AGVLLKKDAFDGQLEITWAQFINTLQRHFLIATKQDPVRPKRPLSSFDLKYIQTHFFGNRS-------------------------IIHQQ
XP_640661       EGS------AGVLLKKDAFDGQLEITWAQFINTLQRHFLIATKQDPVRPKRPLSSYDLKYIQTHFFGNRS-------------------------IIHQQ
XP_004366323    EGS------AGVLLKKDAFDGQLEISWPQFINTLQRHFLIATKQDPVRPKRPLSQFDFKYIQTHFFGNRS-------------------------IIHQQ
EFA82265        EGS------AGVLLKKDAFDGQLEISWAQFINTLQRHFLIATKQDQVRPKRPLSQFDFKYIQQHFFANRS-------------------------IIHQQ
XP_003295153    EES------DGTLLKKDSFGNNNEIPWASYANKLQRHFLRATRQDFMKPTRYLSRHELMYIHHQFFGSKP-------------------------MIPQS
CAC33514        EES------DGTLLKKDSFGNNNEIPWASYANKLQRHFLRATRQDFMKPTRYLSRHELMYIHHQFFGSKP-------------------------MIPQS
EFA83377        EES------DGTLLKKDSFGNNAEIAWASYANKLQRHFLRATRQDSIKPTRYLSRHELIYLHQQFFGGRA-------------------------TITQA
XP_004355663    EES------DGTLLKKDSFGNNAEIPWASYANKLQRHFLRATRQDSIKPTRYLSRHELMYLHQQFFGGRS-------------------------MVSQS
XP_004336217    EES------EGILLKK---GRLWRAGRGDFANVLQRHFLRATRQDLIRPTRQLSDLDFEYLNQTFFEGVQ-------------------------VIGQK
XP_003283502    AEA------AGKLLIADAFNSKDEIPWELFANILHSHILTATHQSS-EIKRKLHSWEFEYIQKFYFDGKT-------------------------SISKS
XP_643781       AEA------AGKLLIADAFAHRDEITWEMFANVLHSHILTATHQTS-EIKRKLHSWEFEYIQKNYFDGKV-------------------------TVSKS
EFA77913        SEA------AGKLLVGEIFEDRSEVPWELFANILHSHVFTTTGQIPNEPKRKLHTWEWDYIQQNHFGSKA-------------------------SLTKT
XP_004362589    AEA------AGKLLIGEAFEDKDEISWELFANMLLGTLFVATSQTPYDPKRKLHTWEFDYIQQTHFAMKP-------------------------RVTRQ
XP_004360534    EGS------EGTLLKKESFNEKFEITWPNFVNILQKHFLKATKQSPIQPTRPLSNYDFLYLNATFFGSRL-------------------------IVHHK
XP_646834       EGS------EGTLLKKETFNEKFEISWPHFVNILQKHFLKATKQSPIQPTRPLSMYDFTYLSNTFFGGKP-------------------------FVSHK
XP_004339235    CDA------AGKLLLFEAFGGQTEIPWQHLANVTHTHFLKATRQDPGRPQRRLNHGEFQYIHYKFFGGQL-------------------------NVTQQ
EFA75761        FKSIANDTPHEALLNEIHHLHQLQRESLEKMHLTQKQFLTTDGANNHDDIYAALQTEQKKLAGQIESELQ-------------------------TLNQM
XP_004338884    STT------EGKLLKKHTFANRSEIPWPAFANAMQIHYIRATKQDPKKPVRPLSNKDIEYLHLTKFNGKMRYVPILSLHRHLHLHLVLYSTRKWTSINQE
```

```
                 EES------AGTLLKKDAFGXQXEIPWAXFANXLQRHFLRATKQDPXRPKRPLSXXEFXYIQQXFFGGKX------------------------XIXQQ

                          1110      1120      1130      1140      1150      1160      1170      1180      1190      1200
XP_003291486     DFDKFWV-----WFGKSMQTLR-YQRHISTLWQEGIIYGYMGRQE--VNDALQGQDPGTFIIRFSERNPGQFGIAYIGIEL-------------------
XP_640661        DFDKFWV-----WFGKSMQTLR-YQRHISTLWQEGIIYGYMGRQE--VNDALQNQDPGTFIIRFSERNPGQFGIAYIGVEM-------------------
XP_004366323     DFDRFWV-----WFGKSMQTLR-YQRHISTLWQEGIIYGYMGRQE--VQDALTNQDPGTFIIRFSERNPGQFGIAYIGSEV-------------------
EFA82265         DFDRFWN-----WFGKSMQTLR-YQRHISTLWQEGIIYGYMGRQE--VNDALTNQDPGTFIIRFSERNPGQFGIAYIGMEV-------------------
XP_003295153     SFDSFWV-----WFGKGLQKLR-YQRHVCSMWQSGLIYGFISRQS--VEEALRNEDQGTFLIRFSERHAGHFAVGYKVDDPDP-----------------
CAC33514         SFDSFWI-----WFGKGLQKLR-YQRHVCSMWQSGLIYGFISRQS--VEEALRNEEQGTFLIRFSERHAGHFAVGYKVDDPDP-----------------
EFA83377         MFDTFWN-----WFGKGLQKLR-YQRHVCSMWQSGLIYGFISRQS--VEEALKNEEMGTFLIRFSERHAGHFAIGYKVDDPDP-----------------
XP_004355663     MFDAFWN-----WFGKGLQKLR-YQRHVCSMWQSGLIYGFISRQS--VEEALRNEEMGTFLIRFSERHAGHFAIGYKVDDPDP-----------------
XP_004336217     SYDAFWE-----WFGKAVQKLR-YQRHICPLWQTGLIYGFLTREG--VKAALINEEVGTFLIRLSERHPGLFAVGYKTDDADV-----------------
XP_003283502     ECKSFWD-----KFGPILQAIH-FKRHIEPLWSSGLIYGLITKSE--CNSFLTNLPEGSFLIRFSDSVPGSFAVAYVTNDD-------------------
XP_643781        ECKTFWD-----RFGPILQTIH-FKRHIEPLWYSGLIYGLITKSE--CNSYLTTLPEGSFLIRFSDSVPGAFAVAYVTNDE-------------------
EFA77913         ECKEFWA-----KFGPILQTIH-FKRHISSLWFEGLICGFISKNE--CNSSLYPAEEGSFLIRFSDSLPGSFAVAYVTNDE-------------------
XP_004362589     EVKEFWV-----KFGPILQTIH-FKRHISSLWFEGLIYGLISKNE--CNSILYNSPEGSFLIRYSDSLPGSFAVAYVTNDE-------------------
XP_004360534     EFDHFWS-----WFGKSLQTLR-YKRHISTLWQNGLIFMFLKRET--VNNILKGQEPGTFVILFSESFAGQLEISYVSLDKQQESPLSLSSSLSNSSSAI
XP_646834        DFDSFWS-----WFGKSIQTLR-YKRHISTLWQNGFIFMFLKRDV--VTQILKNQDVGTFVILFSEAFPGQLEISYVGTDQKD--------SLSKSSNDL
XP_004339235     QAGRFWS-----WFGQVVQTLR-FKRHIANMWFVGLIYGFITKNA--CTEILKNEEIGTFVIRFSENHPGLFAVAYVDDDP-------------------
EFA75761         YSQTILEPNQLCKLDILLQDLS-IQYKQLQLYQNELNYGPGGPELP-VALVITKQPFPMVISKFKQLQEDHLTVQLLVGSNVDIISYSPIRAELIFHSKA
XP_004338884     DYDQFWA-----WFGTILYKIRNHQKHILPMWIKGLIYGFLSREDSDRLLLTQGALPGSFLIRFSDRCAGQFVVVYVTSSPNKKADG----G--------

                 DFDXFWX-----WFGKSLQTLR-YQRHISTLWQEGLIYGFISRQE--VNXALXNQEPGTFLIRFSERHPGQFAVAYVTDDP-------------------

                          1210      1220      1230      1240      1250      1260      1270      1280      1290      1300
XP_003291486     --------------------------P---PRIKHYLVQPNDTAAKKTFPDFLS----EHSQFVNLLQWT-KDANGN-----------PRFLKLHKDTA
XP_640661        --------------------------P---ARIKHYLVQPNDTAAKKTFPDFLS----EHSQFVNLLQWT-KDTNGA-----------PRFLKLHKDTA
XP_004366323     --------------------------P---HRIKHYLVQPNDTAAKKTFPDFLA----EHPQFINILQWS-KGSDGL-----------PRFLKSHKDTA
EFA82265         --------------------------P---HRIKHYLVQPNDTAAKKTFPDFLA----EHPQFINILQWT-KGPDGL-----------PRFLKSHKDTA
XP_003295153     --------------------------E---KRIRHYLVKADDTAGAKKTLPDFLS----ECPQFTKILQLTIDVQTGE-----------PRLRNFPKDVV
CAC33514         --------------------------E---KRIRHYLVKADDTAGAKKTLPDFLS----ECPQFTKILQLTIDVSTGE-----------PRLRNFPKDVV
EFA83377         --------------------------E---KRIRHYLVKADDTAGAKKTLPDFLA----ECPQFTKILQLTIDPTTGE-----------PRLRNFPKDVV
XP_004355663     --------------------------E---KRIRHYLVKADDTAGAKKTLPDFLS----ECPQFTKILQLTIDPITGE-----------PRLRNFPKDVV
XP_004336217     --------------------------Q---KSVRHYLVQPEDTAGAKKTLPDFLF----TCPAEQFLLVVTSDVENGT-----------PKLRKFAKDVA
XP_003283502     --------------------------S---EPVKHYLVKPED-IGANKTLPDFLR----ERHQFKTLYQVDPSKRSLH-----------P--K--NKDTE
XP_643781        --------------------------S---DRVKHYLVKPDD-IGANKTLPDFLR----ERHQFKTLYQVDPSKRSLH-----------P--K--SKDAE
EFA77913         --------------------------N---ERVKHYLVRPEE-IGPNKTLPDFLR----ERYQFKTLYKLDPPNKLMK-----------P--V--PKDEA
XP_004362589     --------------------------Q---ERVKHFLVKPEE-IGANKTLPDYLR----ERYQFQVLYTLDPPLKTLK-----------P--V--PKDTA
XP_004360534     PPPPSLASATTSTTDINGGSGIDQSSQSSATKIKHYLVQPNDTSGSKRTLPDFLN----ECPQFTHILQLNVPSLTGTS------GTAVPQFKKEPKNQI
XP_646834        QSP----TTTTTTT--------------TSTRVKHYLVQANDTSGSKRTLPDFLS----ECNQFTHILQLNIAMIPQT--------ETIPVFKREPKNVV
XP_004339235     --------------------------Y---ERVKHYLVKPED-ISSNKSLPDFLR----EKPQFLYVNQLDPATGELH---------------KLPKDKV
EFA75761         LTKG-SATLIGGIGSVNSGTGGSGGNSALKKHIEKDTQSIDPVKGNAKFPIKFLTGTRKGCVKLHFVLQIKTTDGHLNVSTLSHTESTIPPFRRELKNTV
XP_004338884     ------------------------AGGGDDREVKHYLINPED-IEKKSTLPDFLR----DCENLWFLLQVVRDHDTGV-----------VSLRPKNKDEL

                 --------------------------X---KRIKHYLVKPNDTAGAKKTLPDFLS----ECPQFTXLLQLTXDXXTGX-----------PRFRKXPKDTA

                          1310      1320      1330      1340      1350      1360
XP_003291486     LGSFAPKKSQ----PPPIGGYEP---LSS---------------------------
```

```
XP_640661        LGSFAPKRTA----PVPVGGYEP---LNS---------------------------
XP_004366323     LNSFAPRRAQ----PTPVGGYEP---LSS---------------------------
EFA82265         LGSFAPKKAQ----PAPIGGYEP---LGN---------------------------
XP_003295153     LEPYYSKRET----LPATNGYDS---LPTIIN------------------------
CAC33514         LEPYYSKRET----LPATNGYDS---LPTIL-------------------------
EFA83377         LEPYYSKREA----LPATNGYDS---LPMLN-------------------------
XP_004355663     LEPYYSKREA----LPATNGYDS---LPVLN-------------------------
XP_004336217     LQPYYSKKNP----VAKAKGYDDEIPIPNMLSP-----------------------
XP_003283502     LEPFYSKRIKINANA--NPGYVSG--L-----------------------------
XP_643781        LEPYYSKRLK-QQQT--NPGYVS---L-----------------------------
EFA77913         FKDFYSKRITKVG----NPGYVSE--DSSNKHYSHFKLLYSTSTTCLIIRILKIRN
XP_004362589     FASYYSKRIQEREKAKTNPGYVSG--L-----------------------------
XP_004360534     LEPYYSKRQN--PQSYLGNGYDP---LN----------------------------
XP_646834        LEPYYSKRQN--SQNILGSGYDP---LF----------------------------
XP_004339235     LEGYYSKRQLH---GKPSNGYVLL--------------------------------
EFA75761         LEPYYSKRQN--SQNFLGSGYDP---LT----------------------------
XP_004338884     LASFYSREKS----QTLLLGYDNR--QP----------------------------

                 LEPYYSKRXX----XXXXNGYDS---LPX---------------------------
```

B

Consensus sequence:

```
XXXXXXXLNXXNXXXXXXXXXXXXXXXXXSXXXNXXNNXXXSXXXXXXXXXSXXXXXXXXXXXXXXXXXXXXXXXXXXXXXXXXXXXXX
XXXXXXXXXXXXXXTXXXLXXXXXXXXXXXXXNTXXXXXXXXXXNXXXPXXXXXXXXXXQQXXXQNQXQXXXXXPXXXXXXXXXXXXQXXX
QQQLQXXQQXXXXXXXXXXXPXXXLXNPXXXXXXPXXXXXQXXQQQXXXXXXXXXSXXXXXXQXXQXXXQQXQLQQXQQXXXXXXXXXXX
XXXXXXXXXXXXXXLXXXXXQTQXXXSXXXXXXQSSQQXLSXPXXLXXSXPXGXXXXKPXQEXXNELLXLXHXQKEQLEKMKXXQKQVLX
XPQKEXXXXLQXXQXTLKKQIDEEXXALQQLXXXXILEPTDLRKLXXLLQDLXIQXKQLELLHNELQXLLNPQXPPXXXAALVIXXQPFP
MVITKGKQLXEDPLVVQLLTGARSEXHIXGPVKATMIXESXXXXXNKNNSXKTIENDXQSMDXXXRXAKFPLKFLNGTRKNPVXLKFGXQ
VRDXDGVTXNVESXPSNPFIVITNECQWEESAGTLLKKDAFGXQXEIPWAXFANXLQRHFLRATKQDPXRPKRPLSXXEFXYIQQXFFGG
KXXIXQQDFDXFWXWFGKSLQTLRYQRHISTLWQEGLIYGFISRQEVNXALXNQEPGTFLIRFSERHPGQFAVAYVTDDPXKRIKHYLVK
PNDTAGAKKTLPDFLSECPQFTXLLQLTXDXXTGXPRFRKXPKDTALEPYYSKRXXXXXXNGYDSLPX
```
