# Supplementary figures and images for: Acanthamoeba castellanii STAT Protein
Source: PLoS One. 2014 Oct 22;9(10):e111345. doi: 10.1371/journal.pone.0111345 (PMC4206453; doi:10.1371/journal.pone.0111345)

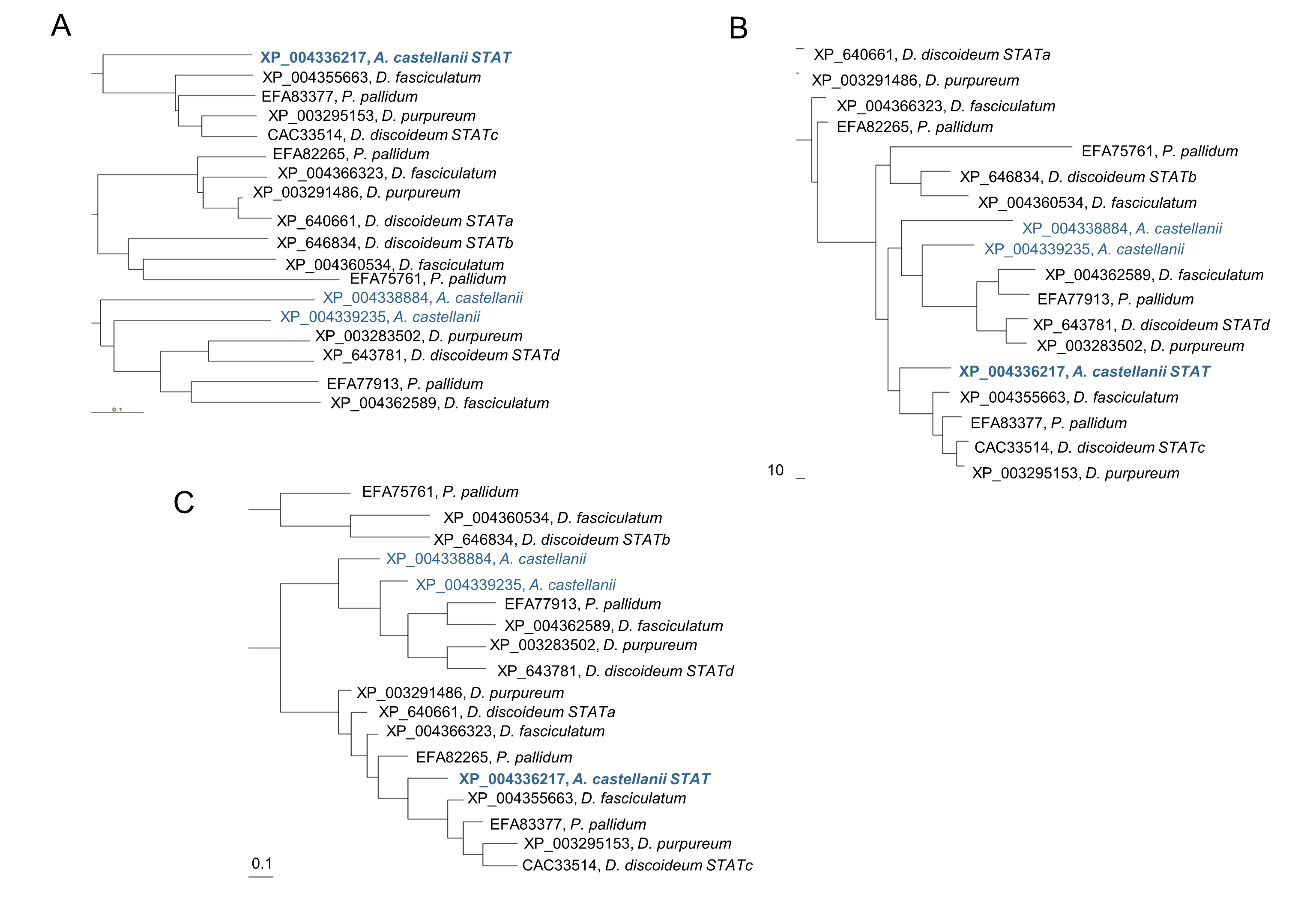

Supplement: Figure S2 — Phylogenetic relationship of the Amoebozoa STAT proteins. The results were obtained with the aid of the following software: (A) Clustal(X), (B) Phylip (maximum likelihood), and (C) SSSSg. (TIF) [file pone.0111345.s002.tif]

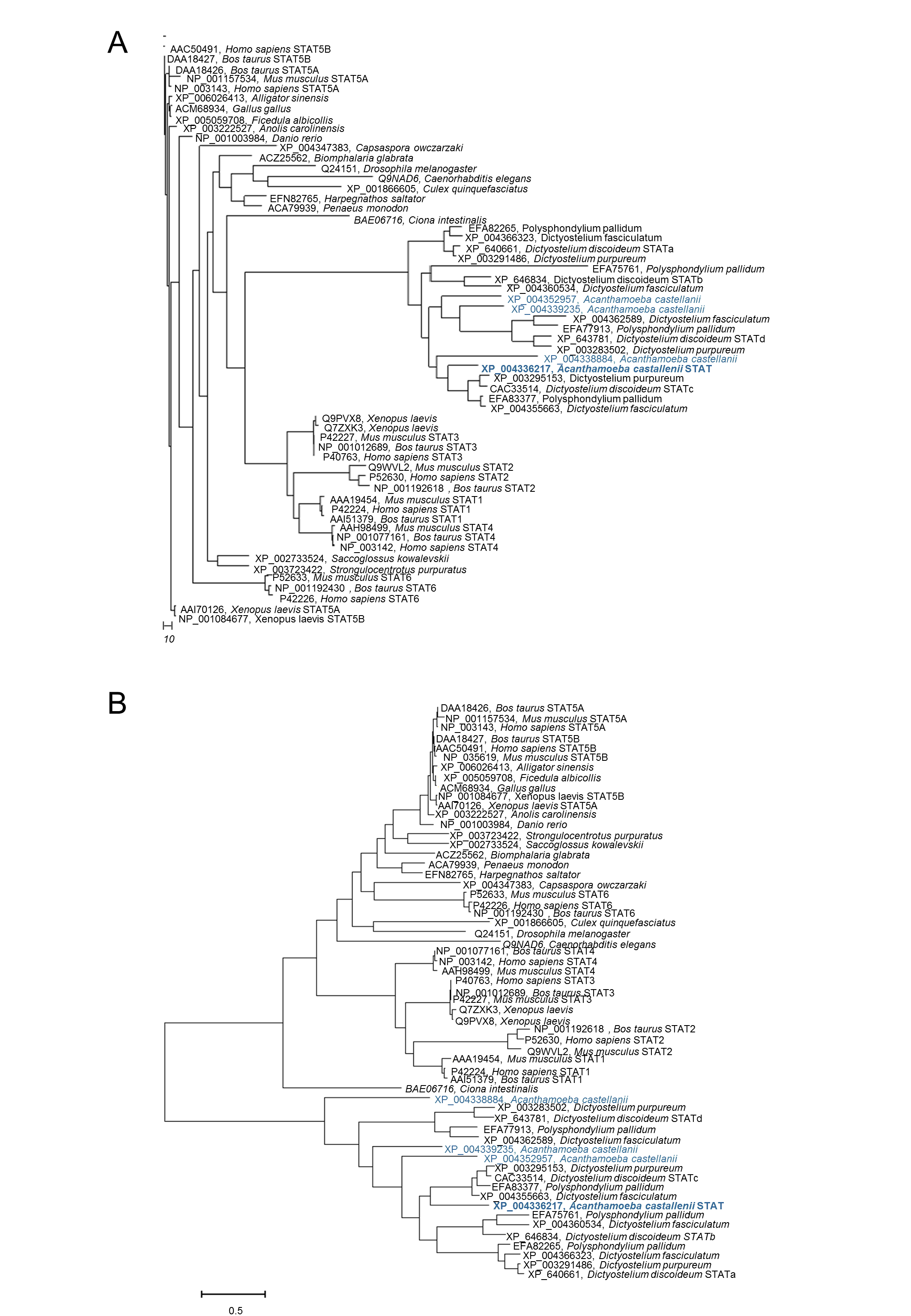

Supplement: Figure S4 — Molecular phylogenetic analysis by Maximum Likelihood method of 59 STAT proteins from selected Ophistokonta and Amoebozoa. The results were obtained with the aid of the following software: (A) Clustal(X) and (B) MEGA5. (TIF) [file pone.0111345.s004.tif]
