# Supplementary material for: Acanthamoeba castellanii STAT Protein
Source: PLoS One. 2014 Oct 22;9(10):e111345. doi: 10.1371/journal.pone.0111345 (PMC4206453; doi:10.1371/journal.pone.0111345)
Supplement: Figure S3 — Multiple sequence alignment and consensus sequence construction of 59 STAT protein sequences. The species names and STAT protein isoforms that correspond to a given accession number are shown in Figure 4. (A) The multiple sequence alignment for 59 STAT proteins is shown. Black background - high conservativity (the same amino acid residue in 69.49% of the sequences), gray background - moderate conservativity (the same amino acid residue in 28.81% of the sequences), gap in consensus sequence (50% of the sequences do not have a position). The last row is the consensus sequence for a given set. (B) Consensus sequence. (PDF) [file pone.0111345.s003.pdf]

Figure S3

A

```
                          10        20        30        40        50        60        70        80        90       100
XP_005059708    ----------------------------------------------------------------------------------------------------
ACM68934        ----------------------------------------------------------------------------------------------------
XP_006026413    ----------------------------------------------------------------------------------------------------
XP_003222527    ----------------------------------------------------------------------------------------------------
NP_001084677    ----------------------------------------------------------------------------------------------------
AAI70126        ----------------------------------------------------------------------------------------------------
NP_003143       ----------------------------------------------------------------------------------------------------
DAA18426        ----------------------------------------------------------------------------------------------------
AAC50491        ----------------------------------------------------------------------------------------------------
NP_035619       ----------------------------------------------------------------------------------------------------
DAA18427        ----------------------------------------------------------------------------------------------------
NP_001157534    ----------------------------------------------------------------------------------------------------
NP_001003984    ----------------------------------------------------------------------------------------------------
XP_003723422    ----------------------------------------------------------------------------------------------------
XP_002733524    ----------------------------------------------------------------------------------------------------
ACA79939        ----------------------------------------------------------------------------------------------------
EFN82765        ----------------------------------------------------------------------------------------------------
ACZ25562        ----------------------------------------------------------------------------------------------------
P42226          ----------------------------------------------------------------------------------------------------
NP_001192430    ----------------------------------------------------------------------------------------------------
P52633          ----------------------------------------------------------------------------------------------------
NP_001192618    ----------------------------------------------------------------------------------------------------
P52630          ----------------------------------------------------------------------------------------------------
Q9WVL2          ----------------------------------------------------------------------------------------------------
NP_001077161    --------------------------------------------------MSNINGG------FVSSYSSSSSSSSSSSPSLPTIPNFHQHNNNKSIAEL
NP_003142       --------------------------------------------------MEVTNNGSNNSSTIASTNPPTSPSTTSTSKSLPPLSFLNSQWENK---QS
AAH98499        ----------------------------------------------------------------------------------------------------
P40763          ----------------------------------------------------------------------------------------------------
P42227          ----------------------------------------------------------------------------------------------------
NP_001012689    ----------------------------------------------------------------------------------------------------
Q7ZXK3          ----------------------------------------------------------------------------------------------------
Q9PVX8          ----------------------------------------------------------------------------------------------------
P42224          ----------------------------------------------------------------------------------------------------
AAI51379        ----------------------------------------------------------------------------------------------------
AAA19454        --------------------------------------------------SPPKSSTNINIEEYHHIQQTGQLQQKTMIPSVTKLKSQSDLMDISYSASL
XP_004355663    ----------------------------------------------------------------------------------------------------
EFA83377        --------------------------------------------------NNNNNSNNNNINNNNNNNNNNNNNNNNNNNNNNNSVKMEKLIELP-SSSN
XP_003295153    ----------------------------------------------------------------------------------------------------
CAC33514        ----------------------------------------------------------------------------------------------------
XP_004336217    ----------------------------------------------------------------------------------------------------
XP_003291486    ----------------------------------------------------------------------------------------------------
XP_640661       ----------------------------------------------------------------------------------------------------
XP_004366323    ----------------------------------------------------------------------------------------------------
EFA82265        ----------------------------------------------------------------------------------------------------
XP_004360534    ----------------------------------------------------------------------------------------------------
```

```
XP_646834         ----------------------------------------------------------------------------------------------------
XP_003283502      ----------------------------------------------------------------------------------------------------
XP_643781         ----------------------------------------------------------------------------------------------------
EFA77913          ----------------------------------------------------------------------------------------------------
XP_004362589      ----------------------------------------------------------------------------------------------------
EFA75761          ----------------------------------------------------------------------------------------------------
XP_004347383      ----------------------------------------------------------------------------------------------------
XP_004339235      ----------------------------------------------------------------------------------------------------
XP_004352957      ----------------------------------------------------------------------------------------------------
Q24151            ----------------------------------------------------------------------------------------------------
XP_001866605      ----------------------------------------------------------------------------------------------------
BAE06716          ----------------------------------------------------------------------------------------------------
XP_004338884      ----------------------------------------------------------------------------------------------------
Q9NAD6            ----------------------------------------------------------------------------------------------------

                  ----------------------------------------------------------------------------------------------------


                           110       120       130       140       150       160       170       180       190       200
XP_005059708      ----------------------------------------------------------------------------------------------------
ACM68934          ----------------------------------------------------------------------------------------------------
XP_006026413      ----------------------------------------------------------------------------------------------------
XP_003222527      ----------------------------------------------------------------------------------------------------
NP_001084677      ----------------------------------------------------------------------------------------------------
AAI70126          ----------------------------------------------------------------------------------------------------
NP_003143         ----------------------------------------------------------------------------------------------------
DAA18426          ----------------------------------------------------------------------------------------------------
AAC50491          ----------------------------------------------------------------------------------------------------
NP_035619         ----------------------------------------------------------------------------------------------------
DAA18427          ----------------------------------------------------------------------------------------------------
NP_001157534      ----------------------------------------------------------------------------------------------------
NP_001003984      ----------------------------------------------------------------------------------------------------
XP_003723422      ----------------------------------------------------------------------------------------------------
XP_002733524      ----------------------------------------------------------------------------------------------------
ACA79939          ----------------------------------------------------------------------------------------------------
EFN82765          ----------------------------------------------------------------------------------------------------
ACZ25562          ----------------------------------------------------------------------------------------------------
P42226            ----------------------------------------------------------------------------------------------------
NP_001192430      ----------------------------------------------------------------------------------------------------
P52633            ----------------------------------------------------------------------------------------------------
NP_001192618      ----------------------------------------------------------------------------------------------------
P52630            ----------------------------------------------------------------------------------------------------
Q9WVL2            ----------------------------------------------------------------------------------------------------
NP_001077161      ----------------------------------------------------------------------------------------------------
NP_003142         ----------------------------------------------------------------------------------------------------
AAH98499          ----------------------------------------------------------------------------------------------------
P40763            ----------------------------------------------------------------------------------------------------
P42227            ----------------------------------------------------------------------------------------------------
NP_001012689      ----------------------------------------------------------------------------------------------------
Q7ZXK3            ----------------------------------------------------------------------------------------------------
Q9PVX8            ----------------------------------------------------------------------------------------------------
```

```
P42224        ----------------------------------------------------------------------------------------------------
AAI51379      ----------------------------------------------------------------------------------------------------
AAA19454      ----------------------------------------------------------------------------------------------------
XP_004355663  ------------------------------------------------------MNYCNSPHTPTTNTLIHHTNIVGSKKPLPEVININNNNNNNNMLAS
EFA83377      ---------------------------------------------------------------MSKKRTLESLQDESGG---FPEVK---AEAVDDLSAD
XP_003295153  -----------------------------------------------------------------MSNPKKRPLDAINGGFDVKQEQD-----SSDFPSD
CAC33514      ---------------------------------------------------------------MSNNNPKKRPLDAISNTFEVKQEE-------PEFSSD
XP_004336217  ----------------------------------------------------------------------------------------------------
XP_003291486  ----------------------------------------------------------------------------------------------------
XP_640661     ----------------------------------------------------------------------------------------------------
XP_004366323  ----------------------------------------------------------------------------------------------------
EFA82265      ----------------------------------------------------------------------------------------------------
XP_004360534  PTPTLINPTTTNNNNNVNNTSKIHPNSLPSLNSIDWNYNNNNNINNGQQQQQLLNSNNSSITNSPTNHHGSMKQMTSSMVLVTEGSGAGAGAPSSPENE
XP_646834     NSPEFLNGVNNNNNGNNNNNNNNNNN----------NNNNNNNNNNN--------NNNNNNNNNNNNNNNSGSGGNNSNNNSNISSPPSSSSTSTSVLSSS
XP_003283502  -----------------------------------------------------------MS---NNWESFN-NNNNNNNNQNQNQNQNQNQNNGNIPRGG
XP_643781     ---------------------------------------------MSQREWTTASTSDLMSNLLNNHNSNNSNNNNNNNNNNNNNNNNNNDNNNNNNNNN
EFA77913      -----------------------------------------------------------MDGSQQQSSANNLNGGNGSAGNGGDTQQHGTTQDKEWMSTS
XP_004362589  -----------------------------------------------------------MDQLNKQLQEQ-LQQQQQLQQGGQQQQQ--QNNGNWMSTS
EFA75761      -----------------------------------------------------------MSNNILNTSNSNTHQHLQHLLHSSNSSGSSSSGSNSNSNNT
XP_004347383  ----------------------------------------------------------------------------------------------------
XP_004339235  ----------------------------------------------------------------------------------------------------
XP_004352957  ----------------------------------------------------------------------------------------------------
Q24151        ----------------------------------------------------------------------------------------------------
XP_001866605  ----------------------------------------------------------------------------------------------------
BAE06716      ----------------------------------------------------------------------------------------------------
XP_004338884  ---------------------------------------------------------------MSSTRKHKAADGDEGEGSSPSSSPRSSPHRAKSPSSS
Q9NAD6        ----------------------------------------------------------------------------------------------------

              ----------------------------------------------------------------------------------------------------

                       210       220       230       240       250       260       270       280       290       300
XP_005059708  ----------------------------------------------------------------------------------------------------
ACM68934      ----------------------------------------------------------------------------------------------------
XP_006026413  ----------------------------------------------------------------------------------------------------
XP_003222527  ----------------------------------------------------------------------------------------------------
NP_001084677  ----------------------------------------------------------------------------------------------------
AAI70126      ----------------------------------------------------------------------------------------------------
NP_003143     ----------------------------------------------------------------------------------------------------
DAA18426      ----------------------------------------------------------------------------------------------------
AAC50491      ----------------------------------------------------------------------------------------------------
NP_035619     ----------------------------------------------------------------------------------------------------
DAA18427      ----------------------------------------------------------------------------------------------------
NP_001157534  ----------------------------------------------------------------------------------------------------
NP_001003984  ----------------------------------------------------------------------------------------------------
XP_003723422  ----------------------------------------------------------------------------------------------------
XP_002733524  ----------------------------------------------------------------------------------------------------
ACA79939      ----------------------------------------------------------------------------------------------------
EFN82765      ----------------------------------------------------------------------------------------------------
ACZ25562      ----------------------------------------------------------------------------------------------------
P42226        ----------------------------------------------------------------------------------------------------
```

```
NP_001192430    ----------------------------------------------------------------------------------------------------
P52633          ----------------------------------------------------------------------------------------------------
NP_001192618    ----------------------------------------------------------------------------------------------------
P52630          ----------------------------------------------------------------------------------------------------
Q9WVL2          ----------------------------------------------------------------------------------------------------
NP_001077161    ----------------------------------------------------------------------------------------------------
NP_003142       ----------------------------------------------------------------------------------------------------
AAH98499        ----------------------------------------------------------------------------------------------------
P40763          ----------------------------------------------------------------------------------------------------
P42227          ----------------------------------------------------------------------------------------------------
NP_001012689    ----------------------------------------------------------------------------------------------------
Q7ZXK3          ----------------------------------------------------------------------------------------------------
Q9PVX8          ----------------------------------------------------------------------------------------------------
P42224          ----------------------------------------------------------------------------------------------------
AAI51379        ----------------------------------------------------------------------------------------------------
AAA19454        ----------------------------------------------------------------------------------------------------
XP_004355663    SY-ITK-EAAANKSPMLDPSPSSSSSSSSTTPPLLLTPNILL---IPNL----------------------------------------------MNNCN
EFA83377        GF-TTN-DDLMSLMTFLD----NGSSQS----------NFMV---KP----------------------------------------------------E
XP_003295153    GFNTTN-DDLMSLMTFLD----NGSGQNQNQNQNQNQNQNQ------------------------------------------------------NQNQN
CAC33514        GFNTTN-DDLMSLMTFLD----NGTGQQNQQNQQNQQPQPQP---QPQPQLPQPQSQQPIYNSNTTTVTVKTEGIATSPLSNASSPISTNNNIYNNTNNN
XP_004336217    ----------------------------------------------------------------------------------------------------
XP_003291486    ----------------------------------------------------------------------------------------------------
XP_640661       -----------------------------------------------------------------------------------------------MSSAE
XP_004366323    ------------------------------------------------------------------------------------------------MASE
EFA82265        ------------------------------------------------------------------------------------------------MSSD
XP_004360534    HSAMEFKSFDRKVASAIDSFGKFESLQKQYESQMGQIMSQME----REQKQRAQ---IEDTCTTLQNENLKLRQDHKSINKKLKELEKSNE-YLMMMLQQ
XP_646834       QSAYMN---DKMVAATLDSIGKMESIQRKYEMQIESLMDQIQGYIEKEQKLRSQCQAVEDINAKLENENLQLKKELFEMSRKFKEIDIINLNNTNNNINN
XP_003283502    SIFFN-----QDSTVDNS---YKASLTIDFPRVDSFGFLRSPTSVLSNT--------------------------------------------PIPNTTG
XP_643781       NNNNN-----NNNNSNNQPLLRNASTNIFFG--DDW--TKQNTVGLLLD--------------------------------------------PLNRGTS
EFA77913        SDVM--------MNSFFGTGLHQPQVSNLGLSGNTFPSTLTKQDTVLNT--------------------------------------------FFNQDVE
XP_004362589    SDVLNYFDSNNNNNTMITPLTKQDTISNMLNAGLTGAGTTSLLPNGGD---------------------------------------------APNNSLD
EFA75761        RFTTNNQQQQQLLFEQIQQQSKMIPNSSSSSTLSKKSSLSHMTNIDDAASAFAVHSGGSGGGGGGSSGGIGSPSSILKIHSNSNNNNSNNNNVSLPSLSD
XP_004347383    --------------------------------------------------------------------------------------------------MA
XP_004339235    ----------------------------------------------------------------------------------------------------
XP_004352957    ------------------------------------MEPQWPGAGDEIGGLSSWLLELGVEGGGLNHSGNGLGSSGNSSGGLLNSGGGSLSGSGGSGSGA
Q24151          ----------------------------------------------------------------------------------------------------
XP_001866605    ----------------------------------------------------------------------------------------------------
BAE06716        -------------------------------------------------------------------------------------------------GDM
XP_004338884    SGNKKRRLDTNGSDESTATTSSSSSSTSTSSSVFPHPALHQLPPHLLNHS------------------------------------------HPGGDLGE
Q9NAD6          ----------------------------------------------------------------------------------------------------

                ----------------------------------------------------------------------------------------------------


                         310       320       330       340       350       360       370       380       390       400
XP_005059708    MAVWIQAQQLQGEALRQMQALYG-QHFPIEVRHYLSQWIESQAWD--SIDLDNPQENVK--------ATQLLEGLIQ--------------ELQKKADHQ
ACM68934        MAVWIQAQQLQGEALRQMQALYG-HHFPIEVRHYLSQWIESQAWD--SIDLDNPQENVK--------ATQLLEGLIQ--------------ELQKKADHQ
XP_006026413    MAVWIQAQQLQGEALRQMQALYG-QHFPIEVRHYLSQWIESQAWD--SIDIDNPQDSIK--------ATQLLDGLVQ--------------ELQKKAEHQ
XP_003222527    MAVWIQAQQLQGDALRQMQALYG-QHFPIEVRHYLSQWIESQPWD--SIDLDNPQEGAK--------ATQLLEGLIQ--------------ELQKKADHQ
NP_001084677    MAVWIQAQQLQGDALRQMQALYG-QHFPIEVRHYLSHWIEAQAWD--SVDPENPQDNLK--------ATQLLEGLVQ--------------ELQKKAEHQ
AAI70126        MAVWIQAQQLQGDALRQMQALYG-QHFPIEVRHYLSQWIEAQTWD--SVDPENPQDNLK--------ATQLLEGLVQ--------------ELQKKAEHQ
```

```
NP_003143       MAGWIQAQQLQGDALRQMQVLYG-QHFPIEVRHYLAQWIESQPWD--AIDLDNPQDRAQ--------ATQLLEGLVQ-------------ELQKKAEHQ
DAA18426        MAGWIQAQQLQGDALRQMQVLYG-QDFPIEVRHYLAQWIESQPWD--AIDLDNPQDRAQ--------ATQLLEGLVQ-------------ELQKKAEHQ
AAC50491        MAVWIQAQQLQGEALHQMQALYG-QHFPIEVRHYLSQWIESQAWD--SVDLDNPQENIK--------ATQLLEGLVQ-------------ELQKKAEHQ
NP_035619       MAMWIQAQQLQGDALHQMQALYG-QHFPIEVRHYLSQWIESQAWD--SIDLDNPQENIK--------ATQLLEGLVQ-------------ELQKKAEHQ
DAA18427        MAVWIQAQQLQGDALHQMQALYG-QHFPIEVRHYLSQWIEGQAWD--SIDLDNPQENIK--------ATQLLEGLVQ-------------ELQKKAEHQ
NP_001157534    MAGWIQAQQLQGDALRQMQVLYG-QHFPIEVRHYLAQWIESQPWD--AIDLDNPQDRGQ--------ATQLLEGLVQ-------------ELQKKAEHQ
NP_001003984    MALWIQAQQLQGDALHQMQSLYG-QHFPIEVRQYLAQWLEAQPWD--AIDLENQQDEFK--------AKRLLDGLIQ-------------ELQKKAEHQ
XP_003723422    MSLIMKLQQLQGGDLEKLQGLYQNLEFPMEIRYYCAHWIEAQNWS--SIDIDNPVFEPD--------AIDLLHGMIK-------------QVQAKIEEL
XP_002733524    MALWARSQQLQGEQLKQMQALYG-PHFPIEIRHHCADWLESQQWH--DYDMTNPAHEQH--------AKQCLEQLLA-------------CVRQKSTDS
ACA79939        MSLWNRAQQLPADDLRRVQGIYG-EQFPIEVRHYLAGWIEDKMQQWNEIDPDNPSHSQY--------AQSLVSQLIQ-------------EIENKALSY
EFN82765        MSLWAKSQQLPNEALKQVHAMYG-DHFPIEVRHFLSSWIEEKMP---DIDPDNPQHEQY--------VTNFVESMIH-------------ELESKAATF
ACZ25562        MSQWAKVQQSP-NIFRQMQQYYP-PHFPMEVRYFLCQWIEEQQW-----DQIDESNHQY--------AELILNDMLT-------------LLRQKAEEL
P42226          MSLWGLVSKMP---PEKVQRLYV--DFPQHLRHLLGDWLESQPWE--FLVGSDAFCCNL--------ASALLSDTVQ-------------HLQASVGEQ
NP_001192430    MSLWGLVSKMP---PEKLQRLYV--DFPQHLRHLLGDWLENQPWE--FLVGSDTFCCNM--------ASALLSATVQ-------------RLQASAGEQ
P52633          MSLWGLISKMS---PEKLQRLYV--DFPQRLRHLLADWLESQPWE--FLVGSDAFCYNM--------ASALLSATVQ-------------RLQATAGEQ
NP_001192618    MAQWEMLQNLDSPFQDQLHELYSNSLLPMDVRQHLAVWIEDQNWQKAALGNDAAMANML--------FLHFLDQLNS-------------ECGRCSQDS
P52630          MAQWEMLQNLDSPFQDQLHQLYSHSLLPVDIRQYLAVWIEDQNWQEAALGSDDSKATML--------FFHFLDQLNY-------------ECGRCSQDP
Q9WVL2          MAQWEMLQNLDSPFLDQLHQVYSQSFLPMDFRQHLASWIEDQNWREAALESDDAKANML--------YFSILDQLN--------------QWDHYSSDP
NP_001077161    MSQWNQVQQLEIKFLEQVDQFYDDN-FPMEIRHLLAQWIENQDWEAAS--NNETMATIL--------LQNLLIQLDE-------------QLGRVSKE-
NP_003142       MSQWNQVQQLEIKFLEQVDQFYDDN-FPMEIRHLLAQWIENQDWEAAS--NNETMATIL--------LQNLLIQLDE-------------QLGRVSKE-
AAH98499        MAQWNQVQQLEIKFLEQVDQFYDDN-FPMEIRHLLAQWIETQDWEVAS--NNETMATIL--------LQNLLIQLDE-------------QSGRVSKE-
P40763          MAQWNQLQQLDTRYLEQLHQLYSDS-FPMELRQFLAPWIESQDWAYAA--SKESHATLV--------FHNLLGEIDQ-------------QYSRFLQE-
P42227          MAQWNQLQQLDTRYLEQLHQLYSDS-FPMELRQFLAPWIESQDWAYAA--SKESHATLV--------FHNLLGEIDQ-------------QYSRFLQE-
NP_001012689    MAQWNQLQQLDTRYLEQLHQLYSDS-FPMELRQFLAPWIESQDWAYAA--SKESHATLV--------FHNLLGEIDQ-------------QYSRFLQE-
Q7ZXK3          MAQWNQLQQLDTRYLEQLHQLYSDS-FPMELRQFLAPWIESQDWAFAA--SKESHATLV--------FHNLLGEIDQ-------------QYSRFLQE-
Q9PVX8          MAQWNQLQQLDTRYLEQLHQLYSDS-FPMELRQFLAPWIESQDWAFAA--SKESHATLV--------FHNLLGEIDQ-------------QYSRFLQE-
P42224          MSQWYELQQLDSKFLEQVHQLYDDS-FPMEIRQYLAQWLEKQDWEHAA--NDVSFATIR--------FHDLLSQLDD-------------QYSRFSLE-
AAI51379        MSQWYELQQLESKYLEQVHQLYDDS-FPMEIRQYLAQWLEKQDWEHAA--NDVSFATIR--------FHDLLSQLDD-------------QYSRFSLE-
AAA19454        MSQWFELQQLDSKFLEQVHQLYDDS-FPMEIRQYLAQWLEKQDWEHAA--YDVSFATIR--------FHDLLSQLDD-------------QYSRFSLE-
XP_004355663    NSNNNNTNLYNHHHNHINSHGQN-IT-TIKTEPVSTLQYPQQPQQQ--QPQQQ---------QQ--QVQQVQQQQQQ-------------PQSQYIHPP
EFA83377        QSLNQTAQLY----TNIQQPQQ-------QQQQQQQQQQQQQQQQQ--QPQLQNIY------TN--NGQQVYQQQPQNG-----------QQQQMISPP
XP_003295153    QNQNQNQNQNQTQQLYTQQPQQT-TI-AIKTEGLSVNSATQNLYQY--PLYTQEVN------QN--IGQT-VPIEPFFQ-IDGAQ----IQQQ-LLQQQ
CAC33514        NNNNNNNNNNNNNNNNNNNNNNN-TA-TPPAIGVQQNSNIP--YSY--PIYTDVTGQQTQHQQN--IGQNSVNIDPYFQTIDGAQ----IQQQQLLQQQ
XP_004336217    --MQGGGPAYPGGGIKMETGQQQ----PAPLLHAPPNSAPSFLYHH--PPSPFGLHP----ATT--PGGVAFGQQQQ-Q-----------QQQTHVSMG
XP_003291486    ---------------------------------------------------------------------------------------------------
XP_640661       FSMD-DFEDTFDSNATISTKDLF-EGSDRLPLNQSINTTIQNLYLP-----NGGFAIGDQ------SQQQYYQAMPP------------LNQSDQFNLG
XP_004366323    FPME-DYDDFDSS--SLTTKNLF-EGGSADKYHDSINTVMQMGYN------TGGFGITQS------VNGTFYSNQP-------------VDYRPVMNGG
EFA82265        FQMDTDFEDFDSS--NLTTKNLF-EG--AEVLNESINTTLNNLQYN--NNNGNGFGMGAIDHAS--QQQQYYQQMHS------------PQQIDYRGTP
XP_004360534    KINTPILQQTTSPPPPPSSLVQQ-QQPPPQLTFTPPSNSFSPIPN----NNNNNIIQKSPSPSN-ISGPHPLNDKSILLLKKKKK----GD-EELVAET
XP_646834       NNNNNNNNNNNNNNNSINNNNNN-GFSPPPLVKYPSNGSLQQDAKRFKIMEQQSQQQQQMQQQQQIQQQKQYQQQQQTTSKRKNNISIDGDKEALVAEA
XP_003283502    LSDIGLNAQYYQLIDSQLPHDEQ-VQK-LQKFHMEQQENLQRQQNQ--QLQSLQLSNGSLQYQTTTTTTTTAAQPLLN-----------EQQFAFQHIN
XP_643781       FSNL-LKSP-TILGDTQQSQQPQ-SQHTMQQLFANDPTYQQHQQEQ--EQQHQMMGN---------TTSPKSFNPYVGL----------SQPMIVTNTG
EFA77913        WSNN-VSNQSNFFMQNSLSNAGH-QQSFGSLLQSIHPNVNSSTAST--TTTTTTTTTSG---GNSTLGQSNPYTNVNYQ----------FQQHQHQQQN
XP_004362589    WASG-RTNSFSSFSFLRTSNIMS-NDTTTSIINTHNQPLQQQIFNP--YINNTSTNNNN---NNIHMGDLSTHNPLHHL----------TSTTSTTSTN
EFA75761        INWNNNSNSNPNINNSSANNNHHHHNHNHHHNHSHTHHSQSSQNSNNINNSSGGVSPLTN------SHQSDYSSNSN-------------SSSSSSNNK
XP_004347383    EAAATPTRWSSISTHPEATAIYG-RDFPLELRHYFAQWIEDQPWAT--MDPDNPEHYES-----IKDRVQTLGEMLH------------AKISQLAASG
XP_004339235    ---------------------------------------------------------------------------------------------------
XP_004352957    WGLDRSLVADFMEN-IFADTALGSSGNTP-----------LSLSGNTNNSSSSSSSFSTGAMPSFAPQTTS----------------------------
Q24151          MSLWKRISS-HVDCEQRMAAYYE-EKGMLELRLCLAPWIEDRIMS-EQITPNTTDQLER-------VALKFNEDLQQK----------LLSTRTASDQA
XP_001866605    --------MTLQPVLAAIGNLYG-VTFPASVQPTVAQYILAQLR-------TAPFN------------QQLPEPILE---------------QTAISLF
BAE06716        SIDRTNTDSLSTNLEKEVTNGNQWNDLEATIPPLQPNELQDIFRDVVLDWQAQAELTEM----M---DTADIEDTTYVG--------QWNRVERLSSIR
```

```
XP_004338884  SLRFSQVARLLDASAEDGGLGLGHSSSGLGTSTDSSFWKMLETEDPSEVTKQMNQTAEL--------QQQLLKTAHLLR----------SSQDGTEAAGG
Q9NAD6        ----------------------------------------------------------------------------------------------------

              MAXWXQXQQLXXXXLXQXQXLYG-XXFPIEVRHYLAQWIESQXWXX-XXDXDNPXXXXX--------AXQLLXQLXQ--------------QLQXKAXEQ

                       410       420       430       440       450       460       470       480       490       500
XP_005059708  VG-EDGFL--LKIKLGHYATQ---------------------------------------LQNTYDR------------CPMELVRCIRHILYHEQRLVR
ACM68934      VG-EDGFL--LKIKLGHYVTQ---------------------------------------LQNTYDR------------CPMELVRCIRHILYHEQRLVR
XP_006026413  VG-EDGFL--LKIKLGNYATQ---------------------------------------LQNTYDR------------CPMDLVRCIRHILYHEQRLVR
XP_003222527  VG-EDGFL--LKIKLGHYATQ---------------------------------------LKNTYEH------------CPLELVRCIRHILYHEQRLVR
NP_001084677  VG-EDGFL--LKIKLGHYATQ---------------------------------------FKNTYER------------CPMELVRCIRHILYHEQRLVR
AAI70126      VG-EDGFL--LKIKLGHYATQ---------------------------------------FKNTYER------------CPMELVRCIRHILYHEQRLVR
NP_003143     VG-EDGFL--LKIKLGHYATQ---------------------------------------LQKTYDR------------CPLELVRCIRHILYNEQRLVR
DAA18426      VG-EDGFL--LKIKLGHYATQ---------------------------------------LQNTYDR------------CPMELVRCIRHILYNEQRLVR
AAC50491      VG-EDGFL--LKIKLGHYATQ---------------------------------------LQNTYDR------------CPMELVRCIRHILYNEQRLVR
NP_035619     VG-EDGFL--LKIKLGHYATQ---------------------------------------LQSTYDR------------CPMELVRCIRHILYNEQRLVR
DAA18427      VG-EDGFL--LKIKLGHYATQ---------------------------------------LQNTYDR------------CPMELVRCIRHILYNEQRLVR
NP_001157534  VG-EDGFL--LKIKLGHYATQ---------------------------------------LQNTYDR------------CPMELVRCIRHILYNEQRLVR
NP_001003984  MG-GDGFL--LKIKLGHYATQ---------------------------------------LKNSYDA------------CPLELVRCVKHILYTEQRLVQ
XP_003723422  SD-DEMFP--IKLKLNNFTQE---------------------------------------LNKRFSN------------QPFAFVRLINHCLESEERLVQ
XP_002733524  VD----FV--TNMRLNEIYLQ---------------------------------------VKQQYDA------------NPLNLVRIIQHCLDTERELVQ
ACA79939      ANNEDLFL--VRMRLDEAATS---------------------------------------FRTRYLNS-----------NPLGLVGIIRQCLNTEHNLVQ
EFN82765      TS-DDFFI--TRIKLTEAAKS---------------------------------------FRHKYLQ------------NPAALFKIIKHCLALEMKYVQ
ACZ25562      TS-QEFFI--IRMKLEESVQK---------------------------------------LDSIVKT------------NPLEMVQVIKNCLAMEQKIIE
P42226        GE--------GSTILQHIST----------------------------------------LESIYQR------------DPLKLVATFRQILQGEKKAVM
NP_001192430  GE--------GNTILQHIST----------------------------------------LETIYQR------------DPLKLVATCRHILQGEKKAVM
P52633        GK--------GNSILPHIST----------------------------------------LESIYQR------------DPLKLVATIRQILQGEKKAVI
NP_001192618  EY----FL--LQHNLRKFYRD---------------------------------------IQA-LPT------------GSTQLAEMIFNLLLEEKRILT
P52630        ES----LL--LQHNLRKFCRD---------------------------------------IQP-FSQ------------DPTQLAEMIFNLLLEEKRILI
Q9WVL2        KS----LL--LQHNLRKFSRD---------------------------------------IQP-FPN------------GPSQLAEMIFNLLLEEQRILI
NP_001077161  KN----LL--LIHNLKRIRKV---------------------------------------LQGKFHG------------NPMHVAVVISNCLREERRILA
NP_003142     KN----LL--LIHNLKRIRKV---------------------------------------LQGKFHG------------NPMHVAVVISNCLREERRILA
AAH98499      KN----LL--LIHNLKRIRKV---------------------------------------LQGKFHG------------NPMHVAVVISNCLREERRILA
P40763        SN----VL--YQHNLRRIKQF---------------------------------------LQSRYLE------------KPMEIARIVARCLWEESRLLQ
P42227        SN----VL--YQHNLRRIKQF---------------------------------------LQSRYLE------------KPMEIARIVARCLWEESRLLQ
NP_001012689  SN----VL--YQHNLRRIKQF---------------------------------------LQSRYLE------------KPMEIARIVARCLWEESRLLQ
Q7ZXK3        SN----VL--YQHNLRRIKQF---------------------------------------LQSTYLE------------KPMEIARIVARCLWEEGRLLQ
Q9PVX8        SN----VL--YQHNLRRIKQF---------------------------------------LQSRYLE------------KPMEIARIVARCLWEEGRLLQ
P42224        NN----FL--LQHNIRKSKRN---------------------------------------LQDNFQE------------DPIQMSMIIYSCLKEERKILE
AAI51379      NN----FL--LQHNIRKSKRN---------------------------------------LQDNFQE------------DPIQMSMIICNCLKEERKILD
AAA19454      NN----FL--LQHNIRKSKRN---------------------------------------LQDNFQE------------DPVQMSMIIYNCLKEERKILE
XP_004355663  PAYIYNGN---GGTLEDQQQ---------------------------------QQQQLQQQHQIQQQQ------------LLQQQILQQQQFQQQQQQQQ
EFA83377      QQPTYYTN---GTNIDDAHKL--------------------------------QQQQMMLQAQAQAQA------------QQQAQVQAQQQAQAQAQAQA
XP_003295153  LQPIQTS-----------------------------------------------PTQIEINQQQQQQQ------------P-------------------
CAC33514      LQPIQQVN---NPQIDQAQIQQQQAQQIQQQQAQ-IQAQQAQIQQQQLEQQHLQQQQFQFQQQQQQQQ------------QQQQQQQQQQQQQQQQQQQQ
XP_004336217  VAPGGGVA---SPL-QH------------------------------------QQQQLHQQQQQQQQG------------VPMHAMLGVPSPGGQQALSP
XP_003291486  ----------------------------------------------------------------------------------------------------
XP_640661     RSNNLTPR---TNQLQQ------------------------------------LQQQQQQQQQPQQQQ------------QQQTYGTQSPIHMSQTPSSP
XP_004366323  QPTYVAPN---S-------------------------------------------------------------------------GPHSPVNMQ--PSSP
EFA82265      RGGSIPSQ---S----Q------------------------------------YLQHS---------------------------NGGSPLHIPQSPSSP
```

```
XP_004360534    LGSFVELTASRKPMLKRSNSEDSFMHSIPYHLN--GGVPGHPATSLNSNNNNVIPTTADGSSSLPMSG-----------DYPFAQFGSLGHIQEEQSKL
XP_646834       LGSFVDY---AKPSLKRSNSEEVFNSSVYKNKNNINNINNNSNSNNNNGNNSLLNDIQNWQQQQQQQQLLHQRKKRKDYDYDYNSTQNGKGIPSNSSNN
XP_003283502    NLPSVFNSNPTITT---NNP--------------------------------MTANLHNPYLNAPQS-----------YTSKMNANTYDDDE-ELNTP
XP_643781       N----FNTTTTTTTTSNNNN--------------------------------NNNNNNNNSNNNNNN-----------NSMNINANTYEDGEDEPKTP
EFA77913        QQ---QQQYNTSSSSPMNT---------------------------------TNDDDEIVATPPT------------PTIIDHDSSMQQQQHQLNNP
XP_004362589    NG---LNTSVTMSSSQQSSP--------------------------------MKEDDYEDQPATPPT-----------PPIHTPPIHHSTPPISYGNS
EFA75761        LGNIMDVP--SSPEFSHLHQQ-------------------------------------QQQQQHHR-----------STTLSSSSSSSSMVVDSANV
XP_004347383    SE--DKFL--LQMRLQTIAADATFDE---------NPTVLVRILKTCMERELIVWAKLHGLESSGLEASG--------MQLLDGMSAISALAALPQSAPE
XP_004339235    ----------------------------------------------------------------------------------------------------
XP_004352957    ----------QQQQQPPQ-----------LQLHYSTSPSTSSSASSTSSSPTAQMP--------------------------TLQLHQQYQQPQQQP--
Q24151          LKFRVVELCALIQRISAVELYT------------------------------------HLRSGLQK-------------ELQLVTEKSVAATAGQSMPL
XP_001866605    ND--------LLTELDRIALT-----------------------------------------------------------LPPGDILQVQLSTAVAILR
BAE06716        NKVVESMIYKFCQETPMLKFRIMFADVIES-------------------------KCWHLIDSNDCQPGG---------INMRRASTIVSCLQEAVAGRL
XP_004338884    NHHHQDASLYASQGGGGHQPQ------------------------------------PSLSQSQLMS------------ALMGSASANELLGEGVVDYP
Q9NAD6          ----------------------------------------------------------------------------------------------------

                XG-XXXFL--LXXXLXHXXTQ-------------------------------------LQXXYXX-----------XPMELVRXIXHILXEEQRLVX

                      510       520       530       540       550       560       570       580       590       600
XP_005059708    EANN--------------------------------------SPSPSGSL---------------VDAMSQKHLQ------INQTFEELRLITQD-SENELKK
ACM68934        EANN--------------------------------------SPSPAGTL---------------VDAMSQKHLQ------INQTFEELRLITQD-SENELKK
XP_006026413    EATN--------------------------------------SPSPAGSL---------------VDTMSQKHLQ------INQIFEELRLFTQD-TENELKK
XP_003222527    EATD--------------------------------------SPSPASSL---------------ADALSQKHLQ------INQTFEELRLVTQD-TENQLKK
NP_001084677    EASN--------------------------------------SSSPVGGL---------------VDTMSQKHLQ------INQTFEELRVITQD-TENGLKK
AAI70126        EASN--------------------------------------SSSPVGGL---------------VDTMSQKHLQ------INQTFEELRVITQE-TENGLKK
NP_003143       EANN--------------------------------------CSSPAGIL---------------VDAMSQKHLQ------INQTFEELRLVTQD-TENELKK
DAA18426        EANN--------------------------------------GSSSAGIL---------------VDAMSQKHLQ------INQTFEELRLVTQD-TENELKK
AAC50491        EANN--------------------------------------GSSPAGSL---------------ADAMSQKHLQ------INQTFEELRLVTQD-TENELKK
NP_035619       EANN--------------------------------------GSSPAGSL---------------ADAMSQKHLQ------INQTFEELRLITQD-TENELKK
DAA18427        EANN--------------------------------------GTSPAGSL---------------ADAMSQKHLQ------INQTFEELRLVTQD-TENELKK
NP_001157534    EANN--------------------------------------CSSPAGVL---------------VDAMSQKHLQ------INQRFEELRLITQD-TENELKK
NP_001003984    EASN--------------------------------------TSSPGSGP---------------VDGTPQRYQQ------INQTFEELRVMTQD-TENDLRK
XP_003723422    NS----------------------------------------MQNRTVE----------------SPQAPPCNKD------ITENITNIRMMTQK-TEEDLSN
XP_002733524    STENE-------------------------------------GHQSEGMQ---------------ENSQTDLYRQ------LDCTFQRLYQMTQE-TEADLQN
ACA79939        QNEN------------------------------------MLGGGVSHA----------------TNMVIEPCAE------IEQELRILHERTRE-TANELRH
EFN82765        QVEG------------------------------------SLGTPLPNIP---------------SNRLIMP-VD------IIQQLTALRQKALD-CADDIRK
ACZ25562        QAEQTTQQQ-------------------------------QLNQDLPASD---------------QQGTNEK---------IMNAIHELLQKTKV-TENDSKN
P42226          EQFR------------------------------------------------------HLPMP---------FHWKQEELKFKT------GLRR
NP_001192430    EQFH------------------------------------------------------HLPMS---------FHWKQEELKFNT------VLRR
P52633          EEFR------------------------------------------------------HLPGP---------FHRKQEELKFTT------ALGR
NP_001192618    WAQRAQLE------------------------------------QREPAP---------------EAPGESQQHE------LDSRILKLQAMKEK-LVKSISQ
P52630          QAQRAQLE------------------------------------QGEPVL---------------ETPVESQQHE------IESRILDLRAMMEK-LVKSISQ
Q9WVL2          QAQRAQEV------------------------------------QPPPAP---------------EAVVESQQLE------IENRIQGLHVDIEF-LVRSIRQ
NP_001077161    AANMPVQG---------------------------------PLEKSLQ-----------------SSSVSERQRN------VEHKVAAIKNSVQM-TEQDTKY
NP_003142       AANMPVQG---------------------------------PLEKSLQ-----------------SSSVSERQRN------VEHKVAAIKNSVQM-TEQDTKY
AAH98499        AANMPIQG---------------------------------PLEKSLQ-----------------SSSVSERQRN------VEHKVSAIKNSVQM-TEQDTKY
P40763          TAATAAQQ----------------------------------GGQANHPT---------------AAVVTEKQQM------LEQHLQDVRKRVQD-LEQKMKV
P42227          TAATAAQQ----------------------------------GGQANHPT---------------AAVVTEKQQM------LEQHLQDVRKRVQD-LEQKMKV
NP_001012689    TAATAAQQ----------------------------------GGQANHPT---------------AAVVTEKQQM------LEQHLQDVRKRVQD-LEQKMKV
Q7ZXK3          TAVAAAQQ----------------------------------GGPASHPN---------------AAVITEKQQM------LEQHLQDVRKKVQD-LEQKMKV
```

```
Q9PVX8         TAAAAAQQ-------------------------------GGPASHPN---------------AAVVTEKQQM------LEQHLQDVRKKVQD-LEQKMKV
P42224         NAQRFNQ---------------------------------AQSGNIQ---------------STVMLDKQKE------LDSKVRNVKDKVMC-IEHEIKS
AAI51379       HAQRISQ---------------------------------AQSGNIQ---------------STVMLDKQKE------LDSKVRNVKDKVMS-IEHEIKT
AAA19454       NAQRFNQ---------------------------------AQEGNIQ---------------NTVMLDKQKE------LDSKVRNVKDQVMC-IEQEIKT
XP_004355663   QQQ----------------------------IQQHVQSTMIQVQQQ----------------QQQVQQQQ--------QQQVQQQLSSPNNN-NQESIII
EFA83377       QQQ----------------------------VMTSPQQILMNPQDL----------------QQQQQQQNQI-----YQQQMGQMLQSPPQQ-LQHIQIQ
XP_003295153   --------------------------------------IQIHQPQT-------------------IQNIPQL-------QPIQTMQQIEQLP-IIHQTIS
CAC33514       QQQQHHQQQQQQQQHQQQQQHQQQQQHQQHQQQQHQQHQQIHQNQLNHQNQLNN--QNQLNNQNQLNNQNQLNHQNQLNQPTQPQMQQIELP-ILHQLPV
XP_004336217   PST----------------------------AAAFSSPHQHHHQQLQ---------------QQQQQQQQQL------LLQQQQQLQQQQQH-FASQTAH
XP_003291486   ----------------------------------------------------------------------------------------------------
XP_640661      LSSPLP------------------------------SPTPFSRQQSYNNNNSNNTSSSQNYNNNNININNNN-------NNNNTNNNNNNNN-GNNSNGN
XP_004366323   ISSPLP------------------------------SPQSFRQSQG-----------QLG----QMPPQQQQ-------QPIMYQQPQQQMQ-PQQQHMM
EFA82265       LMSPS-------------------------------SPFNGRQSSG----------------------G----------IPQQIPPQSPQQI-PQQHYPQ
XP_004360534   KKRRSEFGNKDDPKKKSLSSLPLLTINNTNNNLHNSYHQQHQLQQQKSFNLLNNIKIE----QDQQQQGNSL--KSSATILRKQQQQKLHIP-DLETDVD
XP_646834      NSSNNNSNNNNNNNSNNNNNIIGSISPPHSSQLQQVSSPQQQQQQQKPNGLKLSISSGSI--KDLINSPNKE--QSSKSQYPSSLSQSSSIP-DMDTDVD
XP_003283502   PTPP---------------------------AVEWDSP--PQ---VFR-----------HPTTSFGSPIE----------YS-KNDNEGEQP-TKYQRLT
XP_643781      PTPSNE-------------------------PINWDMPNYRQNTASFHADININNTTNSHPTT-LSSMMKNEI-----PTYSGQVSNEYEQP-TKFQRIS
EFA77913       PPLVYQ-------------------------PSFG-RSASALN--DPLSNLIQNDLGDAHHPTPNGEPNEYE-------PPTKLARTATLQL-QGMPATS
XP_004362589   FLQAGD-------------------------MNLMSSSVVGLMGQQTMSNIFQFG-GNNNNNNNNNNNNNNN-------GQQNTGRSGEFQI-NEYESLT
EFA75761       RLQQ------------------------------------YEASIES--------------LINQVQSAHDR--------EQKQRSYTKSIE-EGYANLE
XP_004347383   AHERDDISFLMMGPSSTSRS-----S--VPVALNSSSSLLGSLPHVEGGARALAVEVSDASGAAAHQLAECVRIAAHKAQATLHHLVGDTQH-VDNELKR
XP_004339235   -----------------------------------------------------------------MEETDLD-------EMNPEFGNEFWKE-LTDDVLD
XP_004352957   --------Q--FQPQHPQQQHYSQLPPFQPQQTQQQQPQFQG-YQPQQQPQPQQSVAIGGG-KQISIPSFAPPTPTPVFPPQTPPGLAPSLTPMGSPAPM
Q24151         NPYNMNNTP---------------------------MVTGYMVDPSD---------------LLAVSNSCNPP-----VVQGIGPIHNVQNTGIASPALG
XP_001866605   QLNP---------------------------------------------------------------------------LQLYMTLIEFGQP-PESSPPT
BAE06716       NDSYMQIR---------------------------VPIDGGRGGNLLSVKVKSELLNWEQELRGRYTQRPMELVQ--VMQKMLKREQYILEMAEKMPLYT
XP_004338884   PLSAS--------------------------------THALLTSFIK---------------QEGASSSGGAT----IASVAGSGVAALPLPPPPHGTIA
Q9NAD6         ----------------------------------------------------------------MMGSSSQE------LQTALTDVSKTCHH-LWEENKD

               XAXX-----------------------------------XXXXXXXX---------------XXXMSQKQXX------IXQXXEELRXXTQD-TENXLKX

                        610       620       630       640       650       660       670       680       690       700
XP_005059708   LQQTQ-------------------EYFIIQYQENMRLQAQFSQLS---QLGPQERM-----------SRETTLQQKKASLEAWLHREAQTLQQYRVDLAE
ACM68934       LQQTQ-------------------EYFIIQYQENMRLQAQFSQLS---QLGPQERL-----------SRETTLQQKKASLEAWLHREAQTLQQYRVELAE
XP_006026413   LQQTQ-------------------EYFIIQYQENMRLQAQFSQLS---QLGPQERM-----------SRETTLQQKKASLEAWLHREAQTLQQYRVELAE
XP_003222527   LQQTQ-------------------EYFIIQYQESLRLQAQLTQLA---QLNPQERM-----------TREPTLQQKKASLEAWLHREAQTLQQYRVELAE
NP_001084677   LQQTQ-------------------EYFIIQYQESLRIQAQFTQLS---QLSPQERI-----------PREATLQQRQSSLEAWLQREAQTLQQYRLDLSE
AAI70126       LQQTQ-------------------EYFIIQYQESLRIQAQFTQLS---QLTPQERM-----------PREATLQQRQSSLEAWLQREAQTLQQYRLDLSE
NP_003143      LQQTQ-------------------EYFIIQYQESLRIQAQFAQLA---QLSPQERL-----------SRETALQQKQVSLEAWLQREAQTLQQYRVELAE
DAA18426       LQQTQ-------------------EYFIIQYQESLRIQAQFAQLA---QLNPQERL-----------SRETALQQKQVSLEAWLQREAQTLQQYRVELAE
AAC50491       LQQTQ-------------------EYFIIQYQESLRIQAQFGPLA---QLSPQERL-----------SRETALQQKQVSLEAWLQREAQTLQQYRVELPE
NP_035619      LQQTQ-------------------EYFIIQYQESLRIQAQFAQLG---QLNPQERM-----------SRETALQQKQVSLETWLQREAQTLQQYRVELAE
DAA18427       LQQTQ-------------------EYFIIQYQESLRIQAQFAQLA---QLNPQERL-----------SRETALQQKQVSLEAWLQREAQTLQQYRVELAE
NP_001157534   LQQTQ-------------------EYFIIQYQESLRIQAQFAQLG---QLNPQERM-----------SRETALQQKQVSLETWLQREAQTLQQYRVELAE
NP_001003984   LQHSQ-------------------EYFIIQYQESLRIQAQIVSLA---SLPPTERA-----------QREASLQSRKSTLETWLTREANTLQKYRQELAE
XP_003723422   LQQKQ-------------------EMFVFQYQENLRFQGRLNQLANSLAESDPNRR-----------AEEVSLRKKKAEVEQVLQTEAQELLRMRMDLAE
XP_002733524   MKQKQ-------------------ELFVFQYQTSLKLQNQLKQLQ-QYPQNDPNRQ-----------AREKQLMAERKEVDTNLQLEAQQLLQNRIDLAE
ACA79939       LEQEQ-------------------ESFALQYHDCAKINAHLSHIQ--SQERTQQNR-----------EMEQSLRRRKELGEQQLAQKVSGLLQLRMALAD
EFN82765       MEHEQ-------------------ESFALSYHECTKLNAQMQHLV--TQPQSQQNF-----------DVDKKIRRQREQQEQILNHKLAGLMQTRLLLAD
ACZ25562       LQHKQ-------------------ESFIIRFSDCQRFTGQLQQLS--QMPNSQEKI-----------NKEAQLRQQKEQIDFQLAQMAQEILHLRVALSK
```

```
P42226         LQHRVG-------------------EIHLLR--EALQKGAEAGQVS-----------------------------------LHSLIETPANGTGPS-EALAM
NP_001192430   LQHRAG-------------------ETRLLR--EALQPGAEAGQVS-----------------------------------LHSLIETPTNGTGPS-EALVT
P52633         LQHRVR-------------------ETRLLR--ESLQQGAKTGQVS-----------------------------------LQNLIDPPVNGPGPS-EDLAT
NP_001192618   LKDQQ-------------------DVFCFRYNIE---KSVR--TP--------------------------SLDPRQTRQMDILQETLNELDRQRKEVLD
P52630         LKDQQ-------------------DVFCFRYKIQ---AKGK--TP--------------------------SLDPHQTKEQKILQETLNELDKRRKEVLD
Q9WVL2         LKDEQ-------------------DVFSFRYTVF---SLKK--TS--------------------------SSDPHQSQQAHVVQATANKVDRMRKEVLD
NP_001077161   LEDLQ-------------------DEFDYRYKTI---QTMD-QGD--------------------------KNSALMNQEVLTLQEMLNSLDFKRKEALS
NP_003142      LEDLQ-------------------DEFDYRYKTI---QTMD-QSD--------------------------KNSAMVNQEVLTLQEMLNSLDFKRKEALS
AAH98499       LEDLQ-------------------DEFDYRYKTI---QTMD-QGD--------------------------KNSILVNQEVLTLQEMLNSLDFKRKEALS
P40763         VENLQ-------------------DDFDFNYKTLKSQGDMQDLNG--------------------------NNQSVTRQKMQQLEQMLTALDQMRRSIVS
P42227         VENLQ-------------------DDFDFNYKTLKSQGDMQDLNG--------------------------NNQSVTRQKMQQLEQMLTALDQMRRSIVS
NP_001012689   VENLQ-------------------DDFDFNYKTLKSQGDMQDLNG--------------------------NNQSVTRQKMQQLEQMLTALDQMRRSIVS
Q7ZXK3         VENLQ-------------------DDFDFNYKTLKSQSDLSELNG--------------------------NNQSVTRQKMQQLEQMLTALDQLRRTIIS
Q9PVX8         VENLQ-------------------DDFDFNYKTLKSQSDLSELNG--------------------------NNQSVTRQKMQQLEQMLTALDQLRRTIIS
P42224         LEDLQ-------------------DEYDFKCKTL--QNREHETNG--------------------------VAKSDQKQEQLLLKKMYLMLDNKRKEVVH
AAI51379       LEDLQ-------------------DEYDFKCKTL--QNREHETNG--------------------------VAKNDQKQEQLLLQKMYLMLDNKRKEVVL
AAA19454       LEELQ-------------------DEYDFKCKTS--QNREGEANG--------------------------VAKSDQKQEQLLLHKMFLMLDNKRKEIIH
XP_004355663   TTSGI-------NG---------QQTLSIQQPQQQMAPQP---HQQQTLSIQPP--------------------QQNIPLPQHLLV-APFGNQA----PQ
EFA83377       SPP---------Q----------QQTIMHTSPPQILQSPP---QSSLQLPTPVPV------------IPQQLQNQQPIPLPPHLLV-VPFGNQP----LQ
XP_003295153   QPSLV-------DQ---------LQPIQTNNNNNISLPSN---EELQQMN--------------NQVQNQLHCNNNSLPLPDHLLINTPYGN-V----LQ
CAC33514       EPQHL-------HP---------IPTHINNNGNNNNNNNN---NGSNSSNSSNGIGSPDDIIEPNILSSIQHCNNNNLPLPDHLLINTPYGN-V----LQ
XP_004336217   MQQLR-------QA---------GGLLLVQSPATVVAAAP---VAAAAAAAP--------------------------GGPAQLQVPSTLEG--------
XP_003291486   ------------------------------------------------------------------------------MPQQSQPQQQAPQTQIS--------
XP_640661      NGNNN-------NN---------NNNNNNNNTNNNNNNNQ---QQQQQQQQQ--------------------QQQQQQQQQQQQGNPNLS--------
XP_004366323   QQQPQ-------YS---------MQPQQQQQQPQQQFQQQ---QQPQIVHQPI---------------------HQPPPQPQQQQPPSTAIS--------
EFA82265       QPQQQ-------QY---------QQQQYQQPQQQMHHQQP---QQMQPHQQQM---------------------HQQPQQQQQQQAPSTAIS--------
XP_004360534   TSDEIGDFS---QN---------NTPMKSSGGIIRNSGS---IPSENDDDP------------------RNGSSSPIEAPMS-D-ANLFKSIA-GTDDT
XP_646834      STDEF-DFGSNSNNNNNNNNNNNNNNNSNNSNNKKRNNSNNNNLGGDDDDSPDS--------------NDRNGSSSPIDMEPSYDGANLFKTVTPGTITT
XP_003283502   TENSV-------------------ELSALNHKLSNDPHYSISFLREQTTKLKS------------------QASTDDLSRNKSFTIN--HYETG---VDQ
XP_643781      TYN---------------------EES-LNDRLRGDNTGSINFLQAETSKLRSR-----------------QSSQDDLERNRSFQMT--HYDTG---CVP
EFA77913       TANYS-------DP---------YEEQISKV---ANLNVHLSFLRSQTEKLEDDV------------EKMRQSSSQNLSQSSFLGGLNKYAGTN---TEK
XP_004362589   KMQRTN--T---QT---------YEAQINKLPVETNLDLQISLLRSHTERIEGDV------------ERLKHSSNSNLSSHSFLQNQ-EYTGTN---TEK
EFA75761       SENLK------------------LKKELFEMNKKLNEILQMKFSPQPTISAPT------------------SSSSSNLPITSKFTSSLAVSGSLPPPPSH
XP_004347383   LQQMQ-------------------EYFVIQYQEFCQLQAVLQRSP----RSDAARG------------QIELVKRRKQEVGTTLSTEANNIIALMQSLNR
XP_004339235   TLYSQ------------------LEQPAFDTSEYYLDDDDDGMLG---STNPGG---------------FVLPEAAHGAMAPAVQVKDEDAVARTVDPAH
XP_004352957   SPTG---------------GLRLEMKPKLEYTESASMTSGKVVHTSFLLQRQPVLDSLWDLQR---------TQNTQLELVRAKQKQLFLAPEK-----
Q24151         MVTPK------------------VELYEVQHQIMQSLNEFGNCAN-ALKLLAQN-----------------YSYMLNSTSSPNAEAAYRSLIDEKAAIVL
XP_001866605   IDQQA-------------------NALQQMVRQCEAQIGSIAQMT---------------------------------TPLQRQPAVTQLTR-------
BAE06716       NEQPQNGSMSSSPGSAMQKTEPMPDMPRVQYIMRDLNQAELCSREITERIERMVGEYQQLHLCTGGNLQVLDTYDPRLIASAEEQRTWKTIIELRDSLVQ
XP_004338884   PSPAS----------------AFSSPTAAASSGKKSGGGHKATRSGSTSPAPQSSS------------STSSGGGGQAAAQHSAAAAAAAAAVAGMNDGS
Q9NAD6         LQG---------------------RFVNELGELQRLQMVIAQLEQQQRLEN-----------------VFTVKQQMTELQKRAATLYEHLTQKRNDIVI

               LQQXQ-------------------EXFIXQYQEXXRXQAQXXQLX---XXXPQX-----------------XLQQXQXQLEXXLQXXAXXLXQXRXELAX


                           710         720         730         740         750         760         770         780         790         800
XP_005059708   KHQKTLQLLRKQQTT--------ILDDELIQWKRRQQLAGNGGPP----------------EGTLDVLQTWCEKLAEIIWQNRQQIRRAEHLCQQLP-IP
ACM68934       KHQKTLQLLRKQQTT--------ILDDELIQWKRRQQLAGNGGPP----------------EGTLDVLQTWCEKLAEIIWQNRQQIRRAEHLCQQLP-IP
XP_006026413   KHQKTLQLLRKQQTT--------ILDDELIQWKRRQQLAGNGGPP----------------EGTLDMLQSWCEKLAEIIWQNRQQIRRAEHVCQHLP-IP
XP_003222527   KHQETLALLRKQQTI--------ILDDELIQWKRRQQLAGNGGPP----------------EGPLDGLQAWCEKLAEIICQNRQQVRRAEHLCQQLP-IP
NP_001084677   KHQKTLQLLRKQQTI--------ILDDELIQWKRRQQLAGNGGPP----------------EGSLDLLQTWCEKLAEIIWQNRQQIRRAEHLCQQLP-IP
```

```
AAI70126        KHQKTLQLLRKQQTI--------ILDDELIQWKRRQQLAGNGGPP---------------EGSLDLLQTWCEKLAEIIWQNRQQIRRAEHLCQQLP-IP
NP_003143       KHQKTLQLLRKQQTI--------ILDDELIQWKRRQQLAGNGGPP---------------EGSLDVLQSWCEKLAEIIWQNRQQIRRAEHLCQQLP-IP
DAA18426        KHQKTLQLLRKQQTI--------ILDDELIQWKRRQQLAGNGGPP---------------EGSLDVLQSWCEKLAEIIWQNRQQIRRAEHLCQQLP-IP
AAC50491        KHQKTLQLLRKQQTI--------ILDDELIQWKRRQQLAGNGGPP---------------EGSLDVLQSWCEKLAEIIWQNRQQIRRAEHLCQQLP-IP
NP_035619       KHQKTLQLLRKQQTI--------ILDDELIQWKRRQQLAGNGGPP---------------EGSLDVLQSWCEKLAEIIWQNRQQIRRAEHLCQQLP-IP
DAA18427        KHQKTLQLLRKQQTI--------ILDDELIQWKRRQQLAGNGGPP---------------EGSLDVLQSWCEKLAEIIWQNRQQIRRAEHLCQQLP-IP
NP_001157534    KHQKTLQLLRKQQTI--------ILDDELIQWKRRQQLAGNGGPP---------------EGSLDVLQSWCEKLAEIIWQNRQQIRRAEHLCQQLP-IP
NP_001003984    KHQRTLALLRKQQTV--------IVDDELIQWKRRQQLAGNGGPS---------------EGGLDILQAWCEKLADMIWQNRQQIRRVEHLTQQLP-IP
XP_003723422    KHRKTLYSLKILQSR--------VLEDELIRWKRQQQLAGIGGPP---------------EGTLDQQQCWCEALAELIWHNRQQVKKVELLRQQLP-IP
XP_002733524    KHQQTFKILEDLLNQ--------TLEDQLVQWKRRQQLASIGGPP---------------EGSLEQIQQWCENLADLIWQNRQQIKNIDLLRQQLP-MK
ACA79939        KHKGTIDRLNSLQQR--------ILDEELINWKRDQQMHGNGKPF--N------------PNKLDQIQEWCEALAEIIWLNRHQIKGCERHQTKIP-IT
EFN82765        KLKEIISKLGNLQSR--------ILDDELIRWKRDQQLSGNGIQF---------------VNNLDSIQEWCEGLAEIIWLTRQQIKEAELMKQKLM-FN
ACZ25562        KYEVTIVHLQELQKE--------ILDNELISWKRRQQLAGNGLVM--D------------STSIDTLQNWCENLADLIWQNRQQIKKSTQLQQQLP-IK
P42226          LLQETTGELEAAKAL--------VLK-RIQIWKRQQQLAGNGAPF---------------EESLAPLQERCESLVDIYSQLQQEVGAAG---GELE-PK
NP_001192430    LLQETVGELEAAQAL--------VLK-RIQIWKRQQQLAGNGAPF---------------EESLAPLQERCESLVDIYSQLQQEVGAAG---GELD-PK
P52633          MLQGTVGDLEATQAL--------VLK-RIQIWKRQQQLAGNGTPF---------------EESLAGLQERCESLVEIYSQLQQEIGAAS---GELE-PK
NP_001192618    ASKALLGQLTTLIE---------LLLPKLEEWKVQQQKACIGAP--P-------------VGGLEQLEKWFTDGAKLLFHLRQLLKELKELSHMVS-YD
P52630          ASKALLGRLTTLIE---------LLLPKLEEWKAQQQKACIRAP--I-------------DHGLEQLETWFTAGAKLLFHLRQLLKELKGLSCLVS-YQ
Q9WVL2          ISKGLVGRLTTLVD---------LLLPKLDEWKVQQQKSCIGAPPPV-------------KSAAEQLEQWLTAGAKFLFHLRQLLKQLKEMSCLR--YQ
NP_001077161    KMTQIVNETDLLMNS--------MLVEELQDWKRRQQIACIGGP--L-------------HSGLDQLQNCFTLLAESLFQLRRQLEKLEEQSSKMT-YE
NP_003142       KMTQIIHETDLLMNT--------MLIEELQDWKRRQQIACIGGP--L-------------HNGLDQLQNCFTLLAESLFQLRRQLEKLEEQSTKMT-YE
AAH98499        KMTQIVNETDLLMNS--------MLLEELQDWKKRQQIACIGGP--L-------------HNGLDQLQNCFTLLAESLFQLRQQLEKLQEQSTKMT-YE
P40763          ELAGLLSAMEYVQKT--------LTDEELADWKRRQQIACIGGP--P-------------NICLDRLENWITSLAESQLQTRQQIKKLEELQQKVS-YK
P42227          ELAGLLSAMEYVQKT--------LTDEELADWKRRQQIACIGGP--P-------------NICLDRLENWITSLAESQLQTRQQIKKLEELQQKVS-YK
NP_001012689    ELAGLLSAMEYVQKT--------LTDEELADWKRRQQIACIGGP--P-------------NICLDRLENWITSLAESQLQTRQQIKKLEELQQKVS-YK
Q7ZXK3          DLASLLSAMEYVQKN--------LTDEELADWKRRQQIACIGGP--P-------------NICLDRLENWITSLAESQLQTRQQIRKLEELQQKVS-YK
Q9PVX8          DLASLLSAMEYVQKT--------LTDEELADWKRRQQIACIGGP--P-------------NICLDRLENWITSLAESQLQIRQQIKKLEELQQKVS-YK
P42224          KIIELLNVTELTQNA--------LINDELVEWKRRQQSACIGGP--P-------------NACLDQLQNWFTIVAESLQQVRQQLKKLEELEQKYT-YE
AAI51379        KIIELLNATELTQKA--------LINDELVEWKRRQQSACIGGP--P-------------NACLDQLQNWFTIVAESLQQVRQQLKKLEELEQKYT-YE
AAA19454        KIRELLNSIELTQNT--------LINDELVEWKRRQQSACIGGP--P-------------NACLDQLQTWFTIVAETLQQIRQQLKKLEELEQKFT-YE
XP_004355663    PHQQIINECLKLHL---------AQKDQLEKMKVVQKQVLAH-PQ--T--------ET------FQMLDNEQNTLKKQIDAELTSLQQIDQTFVLSP-TE
EFA83377        PHQQIINECLKLHL---------AQKDQLEKMKIVQKQVLAH-PQ--K--------ET------FQMLDNEQNTLKKQIDAELTSLQQIDQTFVLSP-TE
XP_003295153    PHQQIINECLKLHL---------AQKEQLEKMKIVQKQVLAH-PQ--K--------ET------FQMLDNEQNTLKKQIDAEITSLQQIDQTFVLSP-PE
CAC33514        PHQQIINECLKLHL---------AQKEQLDKMKIVQKQVLAH-PQ--K--------ET------FQMLDNEQNTLKKQIDAEITSLQQIDQTFVLSP-PE
XP_004336217    -CQQVLEEIYSYKQ---------QQKDQLEKIRQFQKQLMMR-PQ--K--------EG------YDVLLQQHAQLKNQIAEELRTLQALFQQVILPP-AE
XP_003291486    SPQPILDTIYKLLS---------EQEQTLVQMIHEQSILLNRLPPNLD--------ENS--LVLLRQISQKQITLSSQMNTEMSALDATKKGMILEP-TD
XP_640661       SPQPILDTIYKLLS---------EQEQTLVQMIHEQSLLLNRLPPTLD--------ENS--LAPLKSLSQKQITLSGQMNTEMSALDATKKGMILEP-TD
XP_004366323    SPQPILDTIYKLLS---------EQEQTLVQMIHEQSILLNRLPQTLD--------ETT--VQHLSNLSQKQVTLSNQMNTEMSALDATKKGMILDP-TD
EFA82265        SPQPILDTIYKLLS---------EQETNLVKMIHDQSILLNRITQPLD--------EQT--IHSLQALSQQQVTLSSQMNTEMSALDATKKGMILDP-TD
XP_004360534    PHEATLNEIHRLQL---------QQHEVLEKMALAQKQLLIEHHQILTGHIAPTPLNTARIEETYATLQVEQKKLHGQIDSEVTALNQMYSQTILEP-NQ
XP_646834       PQEELLNEIHSLQM---------QQRETIEKMYIAQKQFLSDRSNGFN--------NNN--EEILRSLQSDQTKLGSTLESELQALNQLYSQTILEP-NQ
XP_003283502    ISMEYYNNLLRYN----------TRPENFERAKEKQIQVINGNNKQYS-----------------DELQTLYTTMKKDIDEENNELKNLLSKKILEP-LD
XP_643781       ISTDYYNSLLTRYN---------NRPEFFERAKENQIQVINNNNVQLA-----------------IDLQNIYTSMKKDIDEENNELKNLLTIRILEP-YD
EFA77913        ISQEFYNNLINRYH---------ERNERFLKAKEQQVTVITTHDIVLA-----------------NELHGVYTTMKKEIDEEKAELQNLLSKTILQP-PD
XP_004362589    ISQEFYNSLLNRYH---------ERNERFLKAKENQIKVIHNNDINLA-----------------RELQVVYTDMKKHIEEEKMELKNLLSKTILQP-PD
EFA75761        ADKKKLELQQKKKPR--------GGNGAIEGDEELVAEALGSFVELTS-------TSSS--RKPMLKRSNSEDSFRHNPNQGMSMMSPHAMIMASRG-GD
XP_004347383    QLQANMGDIAELQLT--------VLPDALQAWQRAQRLESDDADQ------------------LDSVQATCEMIAEALWRSREQTRALLLLQTQLP-FD
XP_004339235    FQQQGQQHQHHQQAA--------AEPRQQARPAAAHVPLPAISQD---------------QAKARMVIDQVQQLFATQDQHIEQIRQIQQQLVLQPQKE
XP_004352957    ------------NLFEEVYRLQVDLRNRVDAELRAVEQLYQNTVLTL-GDLNKWLSLR------------------------------------------
Q24151          TMRRSFMYYESLHEM--------VIH-ELKNWRHQQAQAGNGAPFN--------------EGSLDDIQRCFEMLESFIAHMLAAVKELMRVRLVTE--E
XP_001866605    DLWIVVERATDLASQ--------IIGTYLRQWRIGQDRHGLDRAG---------------LSATLDTIQRACEVLASVVWAVRDQIVALPVLGVPVE---
```

BAE06716	TRQVLIHEYHKDQLFNQLQSLLRRVKCEIAEWKRAEAYLSNGKQVAD--------------SSDIDQLEDCCKPLAKLFLQSLDQILKLQHIEVDNLLKE
XP_004338884	AAAALLSQLASMEES---------VDNQLKAMKFQQCDVIDTQLHN---------------RAEMDKLLKAQGDLRKQIEQSVQRLEQLNDTHILTA-TD
Q9NAD6	KLNDGTNFATMLQTQ--------LIGEKLFSWKNAQKLAQIGMPFDNR------------EQLLDEIQIEFEFLADQNWQLNMFSCWMLDLLRRAPQLN
	KHQXTLXXLXKLQXX--------ILDDE**L**IQW**K**RR**Q**QLAGNGG**P**P---------------EGX**L**DX**L**QNWCEX**L**AEIIWQNRQQIKXLEXLXQLLP-XX
	810 820 830 840 850 860 870 880 890 900
XP_005059708	GP--VEEM--
ACM68934	GP--VEEM--
XP_006026413	GP--VEEM--
XP_003222527	GR--IEEM--
NP_001084677	GP--VEEL--
AAI70126	GP--VEEL--
NP_003143	GP--VEEM--
DAA18426	GP--VEEM--
AAC50491	GP--VEEM--
NP_035619	GP--VEEM--
DAA18427	GP--VEEM--
NP_001157534	GP--VEEM--
NP_001003984	GP--TEEL--
XP_003723422	VPH-GQDL--
XP_002733524	CP--GSDL--
ACA79939	PP-GGVDM--
EFN82765	TT-AMQDP--
ACZ25562	FPDGEEDI--
P42226	TR--
NP_001192430	TR--
P52633	TR--
NP_001192618	GDPLNKG---
P52630	DDPLTKG---
Q9WVL2	GDMFAKG---
NP_001077161	GDPIPLQ---
NP_003142	GDPIPMQ---
AAH98499	GDPIPAQ---
P40763	GDPIVQH---
P42227	GDPIVQH---
NP_001012689	GDPIVQH---
Q7ZXK3	GDPIVQH---
Q9PVX8	GDPIVQH---
P42224	HDPITKN---
AAI51379	HDPITKN---
AAA19454	PDPITKN---
XP_004355663	IRN-VVFL--
EFA83377	IRN-VVFL--
XP_003295153	IRN-VIFL--
CAC33514	IRN-VIFL--
XP_004336217	IHK-LIYL--
XP_003291486	LAK-LFAL--
XP_640661	LAK-LFAL--
XP_004366323	LAK-LFAL--

```
EFA82265       LAK-LFAL--------------------------------------------------------------------------------------------
XP_004360534   LCK-LDIL--------------------------------------------------------------------------------------------
XP_646834      LCK-LDIL--------------------------------------------------------------------------------------------
XP_003283502   LRK-IKET--------------------------------------------------------------------------------------------
XP_643781      LRK-IREC--------------------------------------------------------------------------------------------
EFA77913       LHQ-IRKT--------------------------------------------------------------------------------------------
XP_004362589   LHQ-IRVT--------------------------------------------------------------------------------------------
EFA75761       PTSSSMDW--------------------------------------------------------------------------------------------
XP_004347383   RKSYHQPVQQSGSHTPFVAQLNSHSPHHARNSSSHHHMDTATPASSTSPVNSSPQATLSPRPGKSRSSSLSASASPASGLSSMSISSGSNAGGMSSNSSA
XP_004339235   GVRQLQEQQWKLN---------------------------------------------------------------------------------------
XP_004352957   -------------------------------------------------------------------------HQFAIQSSQLDLYQRELLQLTE-----
Q24151         PE--------------------------------------------------------------------------------------------------
XP_001866605   ----------------------------------------------------------------------------------------------------
BAE06716       QE--NMKM--------------------------------------------------------------------------------------------
XP_004338884   AYRLVCLS--------------------------------------------------------------------------------------------
Q9NAD6         DGLAQATIG-------------------------------------------------------------------------------------------

               GXX-VXXX--------------------------------------------------------------------------------------------

                        910       920       930       940       950       960       970       980       990      1000
XP_005059708   ----------------------LSELNGTITDIISALVTSTFIIE-----KQP--------PQVLKTQTKFAATVRLLVGGK--LNVHMNPPQVKATIIS
ACM68934       ----------------------LSELNGTITDIISALVTSTFIIE-----KQP--------PQVLKTQTKFAATVRLLVGGK--LNVHMNPPQVKATIIS
XP_006026413   ----------------------LGDLNSTITDIISALVTSTFIIE-----KQP--------PQVLKTQTKFAATVRLLVGGK--LNVHMNPPQVKATIIS
XP_003222527   ----------------------LVGLNATITDIISALVTSTFIIE-----KQP--------PQVLKTQTKFAATVRLLVGGK--LNVHMNPPQVKATIIS
NP_001084677   ----------------------LSELNATITDIISALVTSTFIIE-----KQP--------PQVLKTQTKFAATVRLLVGGK--LNVHMNPPQVKATIIS
AAI70126       ----------------------LSELNATITDIISALVTSTFIIE-----KQP--------PQVLKTQTKFAATVRLLVGGK--LNVHMNPPQVKATIIS
NP_003143      ----------------------LAEVNATITDIISALVTSTFIIE-----KQP--------PQVLKTQTKFAATVRLLVGGK--LNVHMNPPQVKATIIS
DAA18426       ----------------------LAEVNATITDIISALVTSTFIIE-----KQP--------PQVLKTQTKFAATVRLLVGGK--LNVHMNPPQVKATIIS
AAC50491       ----------------------LAEVNATITDIISALVTSTFIIE-----KQP--------PQVLKTQTKFAATVRLLVGGK--LNVHMNPPQVKATIIS
NP_035619      ----------------------LAEVNATITDIISALVTSTFIIE-----KQP--------PQVLKTQTKFAATVRLLVGGK--LNVHMNPPQVKATIIS
DAA18427       ----------------------LAEVNATITDIISALVTSTFIIE-----KQP--------PQVLKTQTKFAATVRLLVGGK--LNVHMNPPQVKATIIS
NP_001157534   ----------------------LAEVNATITDIISALVTSTFIIE-----KQP--------PQVLKTQTKFAATVRLLVGGK--LNVHMNPPQVKATIIS
NP_001003984   ----------------------LTELNAIVTDIISTLVTSTFIIE-----KQP--------PQVLKTQTKFSATVRLLVGGK--LNVHMNPPQVKATIIS
XP_003723422   ----------------------LPELNRCFMAQLSTLVTSTFIIE-----KQP--------PQVLKKETRFSASVRLLVGGK--LNVHMNPPQVKATIIS
XP_002733524   ----------------------LPQLHNTITSLLSTLVTSTFIID-----KQP--------PQVLKKDTKFSAGVRLLVGGK--LSVHMSPPTVKASIIS
ACA79939       ----------------------LPTLNSHITRLLSSLVTSTFIIE-----KQP--------PQVMKTNTRFTATVRLLVGGK--LNVNMTPPQVRVSIIS
EFN82765       ----------------------FPALNSQITQLLSSLVTSTFIIE-----KQP--------PQVMKTNTRFTSTVRLLVGGK--LNVHMTPPQVRVYIVS
ACZ25562       ----------------------LPRLNDTITGLLSSLVTSTFIVE-----HQP--------PQVLKKDARFGAVVRLLVGGK--LNVHMNPPQVKATIIS
P42226         ----------------------A-SLTGRLDEVLRTLVTSCFLVE-----KQP--------PQVLKTQTKFQAGVRFLLGLRF-LGAPAKPPLVRADMVT
NP_001192430   ----------------------A-ALISRLDEVLRTLVTSSFLVE-----KQP--------PQVLKTQTKFQAGVRFLLGLRF-LGAPAKPPLVRADMVT
P52633         ----------------------A-SLISRLDEVLRTLVTSSFLVE-----KQP--------PQVLKTQTKFQAGVRFLLGLQF-LGTSAKPPMVRADMVT
NP_001192618   ----------------------VDLREAQVTELLQRLLHRAFVVE-----NQPCMPQTLHRPLILKTGSKFTVRTRLLVRLQ------EGNESLTAEVSV
P52630         ----------------------VDLRNAQVTELLQRLLHRAFVVE-----TQPCMPQTPHRPLILKTGSKFTVRTRLLVRLQ------EGNESLTVEVSI
Q9WVL2         ----------------------VDLRNAQVMELLQRLLQRSFVVE-----TQPCMPQTLHRPLILKTGNKFTVRTRLLVRLQ------EGSESLKAEVSV
NP_001077161   ----------------------RAHLLERVTFLIYNLFKNSFVVE-----RQPCMPTHPQRPMVLKTLIQFTVKLRLLIKLP------ELNYQVKVKASI
NP_003142      ----------------------RTHMLERVTFLIYNLFKNSFVVE-----RQPCMPTHPQRPLVLKTLIQFTVKLRLLIKLP------ELNYQVKVKASI
AAH98499       ----------------------RAHLLERATFLIYNLFKNSFVVE-----RQPCMPTHPQRPMVLKTLIQFTVKLRLLIKLP------ELNYQVKVKASI
P40763         ----------------------RPMLEERIVELFRNLMKSAFVVE-----RQPCMPMHPDRPLVIKTGVQFTTKVRLLVKFP------ELNYQLKIKVCI
P42227         ----------------------RPMLEERIVELFRNLMKSAFVVE-----RQPCMPMHPDRPLVIKTGVQFTTKVRLLVKFP------ELNYQLKIKVCI
NP_001012689   ----------------------RPMLEERIVELFRNLMKSAFVVE-----RQPCMPMHPDRPLVIKTGVQFTTKVRLLVKFP------ELNYQLKIKVCI
```

```
Q7ZXK3         ----------------------RPMLEERIVELFRNLMKSAFVVE-----RQPCMPMHPDRPLVIKTGVQFTNKVRLLVKFP------ELNYQLKIKVCI
Q9PVX8         ----------------------RPMLEERIVELFRNLMKSAFVVE-----RQPCMPMHPDRPLVIKTGVQFTNKVRLLVKFP------ELNYQLKIKVCI
P42224         ----------------------KQVLWDRTFSLFQQLIQSSFVVE-----RQPCMPTHPQRPLVLKTGVQFTVKLRLLVKLQ------ELNYNLKVKVLF
AAI51379       ----------------------KQALWDRTFSLFQQLIQSSFVVE-----RQPCMPTHPQRPLVLKTGVQFTVKLRLLVKLQ------ELNYNLKVKVLF
AAA19454       ----------------------KQVLSDRTFLLFQQLIQSSFVVE-----RQPCMPTHPQRPLVLKTGVQFTVKSRLLVKLQ------ESNLLTKVKCHF
XP_004355663   ----------------------IHELTIQSIQLELYHEELQLLVR----PQNP-------PPTIAALVVIEQPFPMVITKCK-----PLEDDPVVVQLLS
EFA83377       ----------------------LHELTIQSIQLELYHEELQLLVR----PQSP-------TPTIAALVVIEQPFPMVITKCK-----PLEDDPVVVQLLS
XP_003295153   ----------------------LHELTIQSIQLELYHEELQLLVR----PQNP-------PPTIAALVVIEQPFPMVITKCK-----PLEDDPVVVQLLS
CAC33514       ----------------------LHELTIQSIQLELYHEELQLLVR----PQNP-------PPTIAALVVIEQPFPMVITKCK-----PLEDDPVVVQLLC
XP_004336217   ----------------------LQDLKLQQIQLELFQQELQRLTLA---PHHPHL-----ARPVCALVIVEQPLPMVVTKGK-----QLEDDPVVVQLLT
XP_003291486   ----------------------KQDLQIQFKQLSLLHNEIQAILN----PQHSS------PKPNVALVLKSQPFPIVISKGK-----QLGENQLVVLILT
XP_640661      ----------------------KQDLQIQFKQLSLLHNEIQSILN----PQHSA------PKPNVALVLKSQPFPVVISKGK-----QLGENQLVVLVLT
XP_004366323   ----------------------KQDLQIQFKQLHLLHNEIQAVLN----PGSAS------SKPNVALVLKTQPFPVVITKGK-----QLGENQLVVQILT
EFA82265       ----------------------RQDLQIQFKQLTLLHHEIQSVLN----PTNQP------TKPNIALVMKTQPFPVVITKGK-----QLSENQLVVQILT
XP_004360534   ----------------------NQELEIQMKQLQIYQLELSRINSPGGGPEQTSASMS--SSHTTALVITKQPFPMVISKFK-----QLQEDHLTVQLLT
XP_646834      ----------------------LQDVSIQLKQLHLYQMELN----YGYGSNEP---------FPATLVIIKQPFPMVISKFK-----QLQEDHLCVQLLT
XP_003283502   ----------------------IEGLKSHLRVVEVLHNELKYIIN----RKKP--------ECCAALIITQQPCSQVIFRGG-----KGMADVFKVQMIF
XP_643781      ----------------------LEGLKSHLRIVDVLYNELKYVLN----RKKP--------ECCAALIITQQPYSQVIFRGG-----KGMGEIFKVQMIF
EFA77913       ----------------------IEGLQSHYRIVDVLYNELQYVLN----KKKP--------ESCCAIILLQQPPSQVIFRGG-----KGIADPYNIELVT
XP_004362589   ----------------------IEGLKSHHRIVDVLYTELQYILN----KKKP--------ESCCAIILLSQPPSQVLFIK------KVIPDAYKVELVT
EFA75761       ----------------------AMGNSSDLDDLKFKKRRSDQFMN------SPP------TEQPPAGTKTKKP-PQFNPSSP-----PNLQRHHSFSINS
XP_004347383   AAMDTEGATNTAASISSNGMVSLEDLHIRATQMLASLIGATFVVE-----TQP--------PQALKTQSKFSAQVRLLVGRT--LNLHMSPPEVLTTIIS
XP_004339235   -------------QQIELELKELQGLHRSVILDPPALHQLRMLLQR-LKIQQQ--------KIELFRQELQLPTPQVLFKGK------TLEDNYVLALLS
XP_004352957   HKAGG-----AQCFATLAMTERPASSVIFKGK------HFGQPYVVHLLTGANVD--L----TCVSKV---KASIVCESH-------------------
Q24151         ----------------------LTHLLEQVQNAQKNLVCSAFIVD-----KQP--------PQVMKTNTRFAASVRWLIGSQ--LGIHNNPPTVECIIMS
XP_001866605   ----------------------LAGLFDRTRDLLALLATKCFVVE-----CQP--------PRVIKTNTKYSVGVRHLLGGQ--LHSRMVGMKLQTCIVS
BAE06716       ----------------------LDYLRQHYENMAKDLVENAFIVE-----KQP--------PQVLMKDKKFMCQLRHLVADG--LSIHQFRFDTQVQLVN
XP_004338884   -----------------------DSFALHARRLRLYQEELFQAQS-----GQP-------VKPVASLVITKQPFPCTVKQSK------SVDDPVDVVLIT
Q9NAD6         ---------------------KLTAITEQLNKLLFMLVSQSFIVS-----VQP--------EPVLKTQHKFVTEVRLLIGDKLGIRQHLVNTNVSVKIIA

               ----------------------LXELNXQITXLXSXLVTSTFIVE-----KQP--------PQVLKTQTKFQAXVRLLVGGK-----HMXPPQVKVXIIS

                         1010      1020      1030      1040      1050      1060      1070      1080      1090      1100
XP_005059708   EQQAKALL----KNESTR--NESSGEILNNCCVM----------------------------EYHQATGTLSAHFRNMSLKRIK--------RSDRRGAE
ACM68934       EQQAKALL----KNESTR--NESSGEILNNCCVM----------------------------EYHQATGTLSAHFRNMSLKRIK--------RSDRRGAE
XP_006026413   EQQAKALL----KNESTR--NESSGEILNNCCVM----------------------------EYHQATGTLSAHFRNMSLKRIK--------RSDRRGAE
XP_003222527   EQQAKALL----KNESTR--NESSGDILNNCCVM----------------------------EYHQATGTLSAHFRNMSLKRIK--------RSDRRGAE
NP_001084677   ELQAKALL----KNENTR--NESSGEILNNCCVM----------------------------EYHQATGTLSAHFRNMSLKRIK--------RSDRRGAE
AAI70126       ELQAKALL----KNENTR--NESSGEILNNCCVM----------------------------EYHQATGTLSAHFRNMSLKRIK--------RSDRRGAE
NP_003143      EQQAKSLL----KNENTR--NECSGEILNNCCVM----------------------------EYHQATGTLSAHFRNMSLKRIK--------RADRRGAE
DAA18426       EQQAKSLL----KNENTR--NECSGEILNNCCVM----------------------------EYHQATGTLSAHFRNMSLKRIK--------RADRRGAE
AAC50491       EQQAKSLL----KNENTR--NDYSGEILNNCCVM----------------------------EYHQATGTLSAHFRNMSLKRIK--------RSDRRGAE
NP_035619      EQQAKSLL----KNENTR--NDYSGEILNNCCVM----------------------------EYHQATGTLSAHFRNMSLKRIK--------RSDRRGAE
DAA18427       EQQAKSLL----KNENTR--NDYSGEILNNCCVM----------------------------EYHQATGTLSAHFRNMSLKRIK--------RSDRRGAE
NP_001157534   EQQAKSLL----KNENTR--NECSGEILNNCCVM----------------------------EYHQATGTLSAHFRNMSLKRIK--------RADRRGAE
NP_001003984   EQQAKAIL----KNENTRK-SESSGDILNNNCVM----------------------------EYHQTTGNLSAHFRTMSLKRIK--------RSDRRGAE
XP_003723422   ESQAKAVL----ASETSSW-NETSGDILNNCGVM----------------------------EYHRETGVLNVTFRNMSLKRIR--------RADRRGSE
XP_002733524   ENQAKALL----HNENANV-NDTSGDILNNCGVM----------------------------EYLKDSGVLSVNFRNMSLKKIK--------REEGRGQE
ACA79939       EAQANALL----KNDQMNK-GEQSGEILNNTGTM----------------------------EYNQTSRQLSVSFRNMQLRKIK--------RAEKKGTE
EFN82765       ETQANALL----KSDKM-K-NDNSGEILNNTGTM----------------------------EYHQTTRQLSVSFRNMQLKKIK--------RAEKKGTE
```

```
ACZ25562       ENQAKDLL----RNDVKAK-NDTSGDILNNTGIM----------------------------EFQPGNGQLSISFRNMQLKKIK--------RADKKGSE
P42226         EKQARELS----VPQGPGAGAESTGEIINNTVPL----------------------------ENSIPGNCCSALFKNLLLKKIK--------RCERKGTE
NP_001192430   EKQARELS----MPQGPGAGAESTGEIINNTVPL----------------------------ENSIPGNCCSALFKNLLLKKIK--------RCERKGTE
P52633         EKQARELS----LAQGPGTGVESTGEIMNNTVPL----------------------------ENSIPSNCCSALFKNLLLKKIK--------RCERKGTE
NP_001192618   DRDSP-------KSQGFR---KFNILTSNQKTLT----------------------------PEKGQTQGLIWDFGYLTLVEQRSG---GSGKGNNKGLL
P52630         DRNPP-------QLQGFR---KFNILTSNQKTLT----------------------------PEKGQSQGLIWDFGYLTLVEQRSG---GSGKGSNKGPL
Q9WVL2         DRNS--------DLPGFR---KFNILTSNQKTLT----------------------------PEKGQRQGLIWDFGFLTLVEQRAV---GAGKGNNKGPL
NP_001077161   DKN----A----STLSNR---RFVLCGTHVKAMS----------------------------IEESSNGSLSVEFRHLQPKEMKS----SAGNKGNEGCH
NP_003142      DKN----V----STLSNR---RFVLCGTNVKAMS----------------------------IEESSNGSLSVEFRHLQPKEMKS----SAGGKGNEGCH
AAH98499       DKN----V----STLSNR---RFVLCGTHVKAMS----------------------------SEESSNGSLSVEFRHLQPKEMKC----STGSKGNEGCH
P40763         DKDSGDVA----ALRGSR---KFNILGTNTKVMN----------------------------MEESNNGSLSAEFKHLTLREQRCG---NGGRANCDASL
P42227         DKDSGDVA----ALRGSR---KFNILGTNTKVMN----------------------------MEESNNGSLSAEFKHLTLREQRCG---NGGRANCDASL
NP_001012689   DKDSGDVA----ALRGSR---KFNILGTNTKVMN----------------------------MEESNNGSLSAEFKHLTLREQRCG---NGGRANCDASL
Q7ZXK3         DKDSGEGA----ALRGSR---KFNILGTNTKVMN----------------------------MEESNNGSLSAEFKHLTLREQRCG---NGGRANCDASL
Q9PVX8         DKDSGDVA----ALRGSR---KFNILGTNTKVMN----------------------------MEESNNGSLSAEFKHLTLREQRCG---NGGRANCDASL
P42224         DKDVNERN----TVKGFR---KFNILGTHTKVMN----------------------------MEESTNGSLAAEFRHLQLKEQK-----NAGTRTNEGPL
AAI51379       DKDVNERN----TVKGFR---KFNILGTHTKVMN----------------------------MEESTNGSLAAEFRHLQLKEQK-----NAGARTNEGPL
AAA19454       DKDVNEKN----TVKGFR---KFNILGTHTKVMN----------------------------MEESTNGSLAAELRHLQLKEQK-----NAGNRTNEGPL
XP_004355663   GTRTE--L----QLIGKV---RATMIVENQQN-S------------------------------KTSGSPKTIETEVVSMDEVQR----LAKYHLKFLNG
EFA83377       GTRTE--L----QLIGKV---KATMIVENQQS-S------------------------------KSSSSPKTIETEVVSMDEVQR----LAKYHLKFLNG
XP_003295153   GTRTE--L----QMIGKV---RATMIVENQQG-S------------------------------KTSSSPKTIETEVVSMDETNR----LAKYHLKFLNG
CAC33514       GTRTE--L----QMIGKV---RATMIVENQQG-S------------------------------KTSSSPKTIETEVVSMDETNR----LAKYHLKFLNG
XP_004336217   GANVE--I----HSFSKV---KVAMICDNQQVKT------------------------------NSSSSSKAIENDTQTMDGVRR----IAKFYLKFLTG
XP_003291486   GARSN--F----HINGPV---KATMICDSHPT--------------------------------NKNNPTTPLEMDSQPIYPATL----TAHFPLKFLAG
XP_640661      GARSN--F----HINGPV---KATMICDSHPT--------------------------------NKNNPTTPLEMDSQPIYPATL----TAHFPLKFLAG
XP_004366323   GARSN--F----HINGPV---KATLLCDSHPT--------------------------------NKNNPQQVLEMDTQPIYPASL----TAHFPLKFLAG
EFA82265       GARSN--F----HINGPV---KATLMCDNHPT--------------------------------NKNNPQQQLEMDSQPIYPATL----TAHFPLKFLAG
XP_004360534   GSNSD--I----VWFSPI---RAELTFHAKALTGPGGSGGSGSGSGASGSGTGKNSGANSQNVVQNNHLKKHIEKDTQVLDMSKG----TAKFPIKFLTG
XP_646834      GANVE--I----VSYSPI---RAELVFHSKNLTK-----------GSSNLGT------------QN-SLKKNIEKDTQVLDPIKG----VAKFPIKFLTG
XP_003283502   GVLQP--D----NISQIS---AVINKSET-------------------------------TTSKKEKTTTVPTLENAESHLNTQTW----EAEFKNIKINV
XP_643781      GVLQP--D----NISPVY---ATINKSESNPN----------------------------STTKKEKPTTTPTLENAEASLNTQTW----EAEFKNIKVNV
EFA77913       GVMMP--D----SISTVT---AQIDKSES-------------------------------ATKKEKSAATPSLENNECSLDKL------KATFTNLKVNI
XP_004362589   GVLSP--E----HVSPVT---AKLDKAES--------------------------------NSKKEKSALP--IENYEIPFEKN------KATFTNLKINA
EFA75761       PVKLESSL----HSSGGIMELSSSPSSSSNQLTSS----------GG--ILK-------KPKKQNNNNNNNNIQTTTTTSTSTPQQ---QSHFPSQMQNI
XP_004347383   EAQAKQVA----QQPAVT---VTDGKRASQPTSIPEDFNAG------------DILNCRKAMECHQPSGALRVIFKNLSLKKIK--------RGSNKDET
XP_004339235   GSNVN--I----QNISKV---KAILVAEE-----------------------------------KNWKNKKPIENDVQAMDSMKR----VLTFHNIKLNV
XP_004352957   ------------LWKGKVAKKSMNEDTKVIELHQH----VVQFQPKFLVG----TRKNAVNLRFSVAVRSDD-----AVHTIESDLSNPFIVITNE-CQW
Q24151         EIQSQRFVTRNTQMDNSSLSGQSSGEIQNASSTM----------------------------EYQQNNHVFSASFRNMQLKKIK--------RAEKKGTE
XP_001866605   ESQARNIQ----QSNIVT--MENSGVLTFDDGAL----------------------------ELDKDSKHLKATFRNLQVKKIQ--------RQERRGAD
BAE06716       TEAACAIVD---GGPSTP--IKPSGKILNNCHKS----------------------------DFVEQTKQLHVHMRNMSLSMRQ--------RGDRGRAD
XP_004338884   GAKSEI-----QAMGQV---KAELINEDYNPIS-------------------------------KKKNSAPAIQNAAETMDESL-----MVTFKRLIFPH
Q9NAD6         EDEAKQLSVDYDAHKEIRN-NKTVGTISNDFEKL-----------------------------TMNERGHLAAKFNNSKLTRIAHRKPPPKGASDLKCAA

               EXQAKXLL----XNXGXR---KXSGEILNNXXXM----------------------------EYXQXTGXLSAEFRNMSLKEIK-----XAXRXDRKGAE

                       1110      1120      1130      1140      1150      1160      1170      1180      1190      1200
XP_005059708   S---VTEEKFTILFESQFSVGGN-E--LVFQVKTLS-LPVVVIVHG-SQDNNATATVLWDNAFA--EPGRVPFAV-PDKVQWPQLCEALNMKFKAEVQS-
ACM68934       S---VTEEKFTILFESQFSVGGN-E--LVFQVKTLS-LPVVVIVHG-SQDNNATATVLWDNAFA--EPGRVPFAV-PEKVQWPQLCEALNMKFKAEVQS-
XP_006026413   S---VTEEKFTILFESQFSVGGN-E--LVFQVKTLS-LPVVVIVHG-SQDNNATATVLWDNAFA--EPGRVPFAV-PDKVLWPQLCEALNMKFKAEVQS-
XP_003222527   S---VTEEKFTILFESQFSVGGN-E--LVFQVKTLS-LPVVVIVHG-SQDNNATATVLWDNAFA--EPGRVPFAV-PDKVMWSQLCEALNMKFKSEVQS-
```

```
NP_001084677   S---VTEEKFTILFESQFSVGGN-E--LVFQVKTLS-LPVVVIVHG-SQDNNATATVLWDNAFA--EPGRVPFIV-PDKVLWPQLCDALNMKFKAEVQS-
AAI70126       S---VTEEKFTILFESQFSVGGN-E--LVFQVKTLS-LPVVVIVHG-SQDNNATATVLWDNAFA--EPGRVPFVV-PDKVVWPQLCDALNMKFKAEVQS-
NP_003143      S---VTEEKFTVLFESQFSVGSN-E--LVFQVKTLS-LPVVVIVHG-SQDHNATATVLWDNAFA--EPGRVPFAV-PDKVLWPQLCEALNMKFKAEVQS-
DAA18426       S---VTEEKFTVLFESQFSVGSN-E--LVFQVKTLS-LPVVVIVHG-SQDHNATATVLWDNAFA--EPGRVPFAV-PDKVLWPQLCEALNMKFKAEVQS-
AAC50491       S---VTEEKFTILFESQFSVGGN-E--LVFQVKTLS-LPVVVIVHG-SQDNNATATVLWDNAFA--EPGRVPFAV-PDKVLWPQLCEALNMKFKAEVQS-
NP_035619      S---VTEEKFTILFDSQFSVGGN-E--LVFQVKTLS-LPVVVIVHG-SQDNNATATVLWDNAFA--EPGRVPFAV-PDKVLWPQLCEALNMKFKAEVQS-
DAA18427       S---VTEEKFTILFESQFSVGGN-E--LVFQVKTLS-LPVVVIVHG-SQDNNATATVLWDNAFA--EPGRVPFAV-PDKVLWPQLCEALNMKFKAEVQS-
NP_001157534   S---VTEEKFTVLFESQFSVGSN-E--LVFQVKTLS-LPVVVIVHG-SQDHNATATVLWDNAFA--EPGRVPFAV-PDKVLWPQLCEALNMKFKAEVQS-
NP_001003984   S---VTEEKFTILFESQFSVGVN-E--LVFHVKTLS-LPVVVIVHG-SQDNNATATVLWDNAFS--EPGRVPFVV-PDKVVWPQLCEALNMKYKSEVQS-
XP_003723422   F---VTEEKFTILFQSQFSVASG-E--LVFQVRTMS-LPVVVVSHG-NQECNALATILWDNAFG--ESGRVPFVV-PDGVPFADMGRALNSKFMLAN---
XP_002733524   I---VTEKKFTILFQSEFSVAGQ-E--LVFQVRTLS-LPVVVIVHG-NQESNASATILWDNSFA--EPGRIPFIV-QEKVHWTRLSQALNRKFSQAC---
ACA79939       S---VMDEKFSLLFQSQFSVGGG-E--LVFQVWTLS-LPVVVIVHG-NQEPHAWATVSWDNAFA--EQGRIPFTV-PEKVPWPQIADMLDTKFKAAT---
EFN82765       S---VMDEKFSLLFQSQFSVGGG-E--LVFQVWTLS-LPVVVIVHG-NQEPHAWATVTWDNAFA--EPGRVPFAV-PDKVPWPQVADALNVKFLSAT---
ACZ25562       A---VTEEKFCILFQSEFSVGGN-E--LVFQVWTLS-LPVVVTVHG-NQECNATATVLWDNAFA--EPGRIPFNV-PDQVDWSHLADQLNMKFMSHT---
P42226         S---VTEEKCAVLFSASFTLGPG-K--LPIQLQALS-LPLVVIVHG-NQDNNAKATILWDNAFS--EMDRVPFVV-AERVPWEKMCETLNLKFMAEVGT-
NP_001192430   S---VTEEKCAVLFSTSLTLGPN-K--LPIQLQALS-LPLVVIVHG-NQDNNAKATILWDNAFS--EMDRVPFVV-AERVPWEKMCETLNLKFMAEVGT-
P52633         S---VTEEKCAVLFSTSFTLGPN-K--LLIQLQALS-LPLVVIVHG-NQDNNAKATILWDNAFS--EMDRVPFVV-AERVPWEKMCETLNLKFMAEVGT-
NP_001192618   S---VTEELHIISFTVKYTYQG-----LKQELTTDS-LPVVIISNM-NQLSIAWASVLWFNLLSSNPQDQQFFSN-PPKAPWNLLGPALSWQFSSYV---
P52630         G---VTEELHIISFTVKYTYQG-----LKQELKTDT-LPVVIISNM-NQLSIAWASVLWFNLLSPNLQNQQFFSN-PPKAPWSLLGPALSWQFSSYV---
Q9WVL2         A---VTEELHVISFVVEYTYQG-----LKMKLQTDT-LPVVIISNM-NQLSFAWASILWFNMLSPNPKNQQFFCQ-APKAPWSLLGPVLSWQFSSYV---
NP_001077161   M---VTEELHSITFETQICLYG-----LTIDLETCS-LPVVMISNV-SQLPNAWASIIWYNVSTNDSQNLVFFNN-PPSATLSQLLEVMSWQFSSYV---
NP_003142      M---VTEELHSITFETQICLYG-----LTIDLETSS-LPVVMISNV-SQLPNAWASIIWYNVSTNDSQNLVFFNN-PPPATLSQLLEVMSWQFSSYV---
AAH98499       M---VTEELHSITFETQICLYG-----LTINLETSS-LPVVMISNV-SQLPNAWASIIWYNVSTNDSQNLVFFNN-PPSVTLGQLLEVMSWQFSSYV---
P40763         I---VTEELHLITFETEVYHQG-----LKIDLETHS-LPVVVISNI-CQMPNAWASILWYNMLTNNPKNVNFFTK-PPIGTWDQVAEVLSWQFSSTT---
P42227         I---VTEELHLITFETEVYHQG-----LKIDLETHS-LPVVVISNI-CQMPNAWASILWYNMLTNNPKNVNFFTK-PPIGTWDQVAEVLSWQFSSTT---
NP_001012689   I---VTEELHLITFETEVYHQG-----LKIDLETHS-LPVVVISNI-CQMPNAWASILWYNMLTNNPKNVNFFTK-PPIGTWDQVAEVLSWQFSSTT---
Q7ZXK3         I---VTEELHLITFETEVYHQG-----LKIDLETHS-LPVVVISNI-CQMPNAWASILWYNMLTNNPKNVNFFTK-PPIGTWDQVAEVLSWQFSSTT---
Q9PVX8         I---VTEELHLITFETEVYHQG-----LKIDLETHS-LPVVVISNI-CQMPNAWASILWYNMLTNNPKNVNFFTK-PPIGTWDQVAEVLSWQFSSTT---
P42224         I---VTEELHSLSFETQLCQPG-----LVIDLETTS-LPVVVISNV-SQLPSGWASILWYNMLVAEPRNLSFFLT-PPCARWAQLSEVLSWQFSSVT---
AAI51379       I---VTEELHSLSFETQLCQPG-----LVIDLETTS-LPVVVISNV-SQLPSGWASILWYNMLVAEPRNLSFFLN-PPCARWSQLSEVLSWQFSSVT---
AAA19454       I---VTEELHSLSFETQLCQPG-----LVIDLETTS-LPVVVISNV-SQLPSGWASILWYNMLVTEPRNLSFFLN-PPCAWWSQLSEVLSWQFSSVT---
XP_004355663   ----TRKNPVTLKFGMQVQVVGG----TPVNIESPPTSPFIVITNE-CQYEESDGTLLKKDSFG--NNAEIPWAS-YANKLQRHFLRATRQDSIKPT---
EFA83377       ----TRKNPVTLKFGMQVQVVGG----TPVNIESPPSSPFIVITNE-CQYEESDGTLLKKDSFG--NNAEIAWAS-YANKLQRHFLRATRQDSIKPT---
XP_003295153   ----TRKNPVTLKFGMQVQVVGG----TPVNIESPPSSPFIVITNE-CQYEESDGTLLKKDSFG--NNNEIPWAS-YANKLQRHFLRATRQDFMKPT---
CAC33514       ----TRKNPVTLKFGMQVQVVGG----TAVNIESPPTSPFIVITNE-CQYEESDGTLLKKDSFG--NNNEIPWAS-YANKLQRHFLRATRQDFMKPT---
XP_004336217   ----TRKNPVRLRFGIQLQINQN-GAAQTVTVESNSSRPFIVITNE-CQWEESEGILLKK---G--RLWRAGRGD-FANVLQRHFLRATRQDLIRPT---
XP_003291486   ----TRKCSVNLKFGVNIRDLDN----VTTTVESDASNPFVVITNE-CQWEGSAGVLLKKDAFD--GQLEITWAQ-FINTLQRHFLIATKQDPVRPK---
XP_640661      ----TRKCSVNLKFGVNIRDLDN----VTTTVESDASNPFVVITNE-CQWEGSAGVLLKKDAFD--GQLEITWAQ-FINTLQRHFLIATKQDPVRPK---
XP_004366323   ----TRKSSVNLKFGVNIRDMDS----VTTAVESDFSNPFVVITNE-CQWEGSAGVLLKKDAFD--GQLEISWPQ-FINTLQRHFLIATKQDPVRPK---
EFA82265       ----TRKSSVNLKFGVNVRDMDS----VTTAVESDSSNPFVVITNE-CQWEGSAGVLLKKDAFD--GQLEISWAQ-FINTLQRHFLIATKQDQVRPK---
XP_004360534   ----TRKGCVKLHFQLQARTTDG----HTFNIPSGSSQPFIVITND-CQWEGSEGTLLKKESFN--EKFEITWPN-FVNILQKHFLKATKQSPIQPT---
XP_646834      ----TRKSCVKLHFVLQIKTSDG----HIINVPSSTSQPFIVITND-CQWEGSEGTLLKKETFN--EKFEISWPH-FVNILQKHFLKATKQSPIQPT---
XP_003283502   S---TRMTPSSLRFIANYKEKTS-GKTVEKQVESVPSNPVIVITNE-SQWAEAAGKLLIADAFN--SKDEIPWEL-FANILHSHILTATHQSS-EIK---
XP_643781      S---TRMTPSSLKFVATYKEKTS-GKTVEKDVESVPGSPIIVITNE-SQWAEAAGKLLIADAFA--HRDEITWEM-FANVLHSHILTATHQTS-EIK---
EFA77913       S---TRMNPSHLKFTATVKDKGN---KASKSIESVQTNPIVVITNE-SQWSEAAGKLLVGEIFE--DRSEVPWEL-FANILHSHVFTTTGQIPNEPK---
XP_004362589   S---TRMTPTPLKFSATIRDKSN---RQGKPIESVPTNPILVITNE-SQWAEAAGKLLIGEAFE--DKDEISWEL-FANMLLGTLFVATSQTPYDPK---
EFA75761       GQLPEMETDVDSTDEFDFKGSTG-MP-RSGDGDSPDSNENSRNSSSPIEAPMSDANLFKSIANDTPHEALLNEIH-HLHQLQRESLEKMHLTQKQFLT--
XP_004347383   ----VTDEKFALLFQSTFSVGGG-E--LLVAVRTLS-LPCVVIVHN-NQQHSAEATILWDNSFA--TMDRAPFEV-PEVVSWPDMLTALQHHFSLAT---
XP_004339235   S---TRMSMVYLKFAVQVTLQNG----GTHTIESAGSSPIIVITNE-SQWCDAAGKLLLFEAFG--GQTEIPWQH-LANVTHTHFLKATRQDPGRPQ---
XP_004352957   AEAEGTLIKKEAFG--GHLEIPWAR-FANVFHMHFIRATRQDVNKPA----------RAFHAGDFEYLHDKFFG----------NKQ-----MVSYKDFD
Q24151         S---VMDEKFALFFYTTTTVND-----FQIRVWTLS-LPVVVIVHG-NQEPQSWATITWDNAFA--EIVRDPFMI-TDRVTWAQLSVALNIKFGSCT---
```

```
XP_001866605  S---VTDEKFAFLFDLAFAVGD-----LRFSVWTIS-QPVVVIVHG-NQETAAKATIVWDNAFA--DPSRIPFEI-SERMGWNVLAEMLNRKFRSMLL--
BAE06716      C---VAEEKFCMVFRAFISLRFPSKAEYDVVAQTFS-LPIVVISHG-KQEADAQATVFWDNAFS--ESVRRPFEV-PDSVPWNWILDGLNCLWKKECEKE
XP_004338884  G---SRVKSVNMRFAQEVTMNGS----SM-RLLSEPTKPFIVMTNH-GQRSTTEGKLLKKHTFA--NRSEIPWPA-FANAMQIHYIRATKQDPKKPV---
Q9NAD6        SMQAATDQKYALLFFITPFQMGNLSKEEQFDVWTLS-LPIMVTVHG-SQDCDAQVAILWHRAFA--SISRNPNTTDVTAVTWDNLAIMLRNKFSLFTG--

              S---VTEEKFTILFESQFSVGGN----LVFQVETLS-LPVVVIVNG-SQDXNAWATILWDNAFA--EPGRVPFAV-PDKVLWXQLLEALNQKFKSEV---

                     1210      1220      1230      1240      1250      1260      1270      1280      1290      1300
XP_005059708  ------SRGLTKENLVFLAQKLFNSTS--T-HLEDYSS---TTVSWSQFNR-ENLPGRNYTFWQWFDGVMEVL---KKHLKPHWNDGAILGFVNKQQAH-
ACM68934      ------SRGLTKENLVFLAQKLFNSTS--S-HLEDYSS---TTVSWAQFNR-ENLPGRNYTFWQWFDGVMEVL---KKHLKPHWNDGAILGFVNKQQAH-
XP_006026413  ------NRGLTKENLVFLAQKLFNSTS--N-HLEDYNS---MTVSWSQFNR-ENLPGRNYTFWQWFDGVMEVL---KKHLKPHWNDGAILGFVNKQQAH-
XP_003222527  ------SRGLTKDNLLFLAQKLFNSNS--S-HIEDYNN---MPVSWAQFNR-ENLPGRNYTFWQWFDGVMEVL---KKHLKPHWNDGAILGFVNKQQAH-
NP_001084677  ------NRGLSDENLLFLAQKLFNSNL--S-HKEDYSI---MTVSWAQFNR-ENLPGWNYTFWQWFDGVMEVL---KKHLKPHWNDGAILGFVNKQQAH-
AAI70126      ------NRGLNDENLVFLAQKLFNSNL--S-HKEEYSN---MTVSWAQFNR-ENLPGWNYTFWQWFDGVMEVL---KKHLKPHWNDGAILGFVNKQQAH-
NP_003143     ------NRGLTKENLVFLAQKLFNNSS--S-HLEDYSG---LSVSWSQFNR-ENLPGWNYTFWQWFDGVMEVL---KKHHKPHWNDGAILGFVNKQQAH-
DAA18426      ------NRGLTKENLVFLAQKLFNSSS--S-HLEDYNG---MSVSWSQFNR-ENLPGWNYTFWQWFDGVMEVL---KKHHKPHWNDGAILGFVNKQLAH-
AAC50491      ------NRGLTKENLVFLAQKLFNNSS--S-HLEDYSG---LSVSWSQFNR-ENLPGRNYTFWQWFDGVMEVL---KKHLKPHWNDGAILGFVNKQQAH-
NP_035619     ------NRGLTKENLVFLAQKLFNISS--N-HLEDYNS---MSVSWSQFNR-ENLPGRNYTFWQWFDGVMEVL---KKHLKPHWNDGAILGFVNKQQAH-
DAA18427      ------NRGLTKENLVFLAQKLFNSSS--S-HLEDYNG---MSVSWSQFNR-ENLPGRNYTFWQWFDGVMEVL---KKHLKPHWNDGAILGFVNKQQAH-
NP_001157534  ------NRGLTKENLVFLAQKLFNISS--N-HLEDYNS---MSVSWSQFNR-ENLPGWNYTFWQWFDGVMEVL---KKHHKPHWNDGAILGFVNKQQAH-
NP_001003984  ------DRGLSEDNLVFLAQKLFSSSS--N-NPEDYSS---MTITWSQFNR-ESLPGRNFTFWQWFDGVIELM---KKHLKSHWNDGAILGFLNKQQAQ-
XP_003723422  ------GRSLSDANLLYLAQKAFSHHN--VGQTEDFSN---VYITWSIFNR-DPLPNRTFTFWRWFHGVLELT---RKHLRGPWNDGSIMGFVSRSMAH-
XP_002733524  ------GRQLTPQNLKYLAVKAFDGHS--T-PDMDFDN---MMISWANFNR-ENLSGKQFTFWKWFHGIIEIT---KKHLREPWQEGYVVGFVSKGHAQ-
ACA79939      ------GRGLTEDNLKFLAGKAFR--N--P-QVQDFTN---MMLSWSQFCK-EPLSERNFTFWEWFFAVMKVT---REHLRQQWNDGSIMGFVGRRQAE-
EFN82765      ------GRSLMDENLRFLAEKAFRGSN--S-NGQDYTN---MLLSWAQFCK-EPLPERNFTFWEWFYAVMKLT---REHLRGPWSDGVILGFVRKKQAE-
ACZ25562      ------GKGLSATNLSYIASKLFG--------NKDSS-----MVSWTQFNK-DTLSGRSFTFWEWFYAIQKLT---KEHLKDLWNDEAIIGFVSKNQAQ-
P42226        ------NRGLLPEHFLFLAQKIFN-DN--SLSMEAFQH---RSVSWSQFNK-EILLGRGFTFWQWFDGVLDLT---KRCLRSYWSDRLIIGFISKQYVT-
NP_001192430  ------NRGLLPEHFLFLAQKIFN-DN--SLSIEAFQH---RSVSWSQFNK-EILLGRGFTFWQWFDGVLDLT---KRCLRSYWSDRLIIGFISKQYVT-
P52633        ------SRGLLPEHFLFLAQKIFN-DN--SLSVEAFQH---RCVSWSQFNK-EILLGRGFTFWQWFDGVLDLT---KRCLRSYWSDRLIIGFISKQYVT-
NP_001192618  ------NRGLDSDQLGMLRDKLFG-------HNSRTEG---ASLSWVDFIKRESPPGK-LPFWTWLDKILDLI---HDHLKDLWKDGRIMGFVSRNQVR-
P52630        ------GRGLNSDQLSMLRNKLFG-------QNCRTED---PLLSWADFTKRESPPGK-LPFWTWLDKILELV---HDHLKDLWNDGRIMGFVSRSQER-
Q9WVL2        ------ARGLDSEQLGMLRTKLFG-------KSCKMED---ALLSWVDFCKRESPPGK-IPFWTWLDKILELV---HDHLKDLWKDGRIMGFVSRNQER-
NP_001077161  ------GRGLNSDQLNMLAEKLTV-------QSSYNDG----HLTWAKFCK-EHLPGKSFTFWTWLEAILDLI---KKHILPLWIDGYIMGFVSKEKER-
NP_003142     ------GRGLNSDQLHMLAEKLTV-------QSSYSDG----HLTWAKFCK-EHLPGKSFTFWTWLEAILDLI---KKHILPLWIDGYVMGFVSKEKER-
AAH98499      ------GRGLNSEQLNMLAEKLTV-------QSNYNDG----HLTWAKFCK-EHLPGKTFTFWTWLEAILDLI---KKHILPLWIDGYIMGFVSKEKER-
P40763        ------KRGLSIEQLTTLAEKLLG-------PGVNYSG---CQITWAKFCK-ENMAGKGFSFWVWLDNIIDLV---KKYILALWNEGYIMGFISKERER-
P42227        ------KRGLSIEQLTTLAEKLLG-------PGVNYSG---CQITWAKFCK-ENMAGKGFSFWVWLDNIIDLV---KKYILALWNEGYIMGFISKERER-
NP_001012689  ------KRGLSIEQLTTLAEKLLG-------PGVNYSG---CQITWAKFCK-ENMAGKGFSFWVWLDNIIDLV---KKYILALWNEGYIMGFISKERER-
Q7ZXK3        ------KRGLSIEQLTTLAEKLLG-------PGVNYSG---CQITWAKFCK-ENMAGKGFSFWVWLDNLIDLV---KKYMLALWNEGYIIGFISKERER-
Q9PVX8        ------KRGLSIEQLTTLAEKLLG-------PGVNYSG---CQITWAKFCK-ENMAGKGFSFWVWLDNIIDLV---KKYILALWNEGYIMGFISKERER-
P42224        ------KRGLNVDQLNMLGEKLLG-------PNASPDG----LIPWTRFCK-ENINDKNFPFWLWIESILELI---KKHLLPLWNDGCIMGFISKERER-
AAI51379
------KRGLNVDQLNMLGEKLLG-------PNAGPDG----LIPWTRFCK-ENINDKNFPFWLWIESILELI---KKHLLALWNDGCIVGFISKERER-
AAA19454      ------KRGLNADQLSMLGEKLLG-------PNAGPDG----LIPWTRFCK-ENINDKNFSFWPWIDTILELI---KNDLLCLWNDGCIMGFISKERER-
XP_004355663  -------RYLSRHELMYLHQQFFG----------GRS-----MVSQSMFDA----------FWNWFGKGLQKLR-YQRHVCSMWQSGLIYGFISRQSVE-
EFA83377      -------RYLSRHELIYLHQQFFG----------GRA-----TITQAMFDT----------FWNWFGKGLQKLR-YQRHVCSMWQSGLIYGFISRQSVE-
XP_003295153  -------RYLSRHELMYIHHQFFG----------SKP-----MIPQSSFDS----------FWVWFGKGLQKLR-YQRHVCSMWQSGLIYGFISRQSVE-
CAC33514      -------RYLSRHELMYIHHQFFG----------SKP-----MIPQSSFDS----------FWIWFGKGLQKLR-YQRHVCSMWQSGLIYGFISRQSVE-
XP_004336217  -------RQLSDLDFEYLNQTFFE----------GVQ-----VIGQKSYDA----------FWEWFGKAVQKLR-YQRHICPLWQTGLIYGFLTREGVK-
XP_003291486  -------RPLSSFDLKYIQTHFFG----------NRS-----IIHQQDFDK----------FWVWFGKSMQTLR-YQRHISTLWQEGIIYGYMGRQEVN-
```

```
XP_640661       -------RPLSSYDLKYIQTHFFG----------NRS-----IIHQQDFDK----------FWVWFGKSMQTLR-YQRHISTLWQEGIIYGYMGRQEVN-
XP_004366323    -------RPLSQFDFKYIQTHFFG----------NRS-----IIHQQDFDR----------FWVWFGKSMQTLR-YQRHISTLWQEGIIYGYMGRQEVQ-
EFA82265        -------RPLSQFDFKYIQQHFFA----------NRS-----IIHQQDFDR----------FWNWFGKSMQTLR-YQRHISTLWQEGIIYGYMGRQEVN-
XP_004360534    -------RPLSNYDFLYLNATFFG----------SRL-----IVHHKEFDH----------FWSWFGKSLQTLR-YKRHISTLWQNGLIFMFLKRETVN-
XP_646834       -------RPLSMYDFTYLSNTFFG----------GKP-----FVSHKDFDS----------FWSWFGKSIQTLR-YKRHISTLWQNGFIFMFLKRDVVT-
XP_003283502    -------RKLHSWEFEYIQKFYFD----------GKT-----SISKSECKS----------FWDKFGPILQAIH-FKRHIEPLWSSGLIYGLITKSECN-
XP_643781       -------RKLHSWEFEYIQKNYFD----------GKV-----TVSKSECKT----------FWDRFGPILQTIH-FKRHIEPLWYSGLIYGLITKSECN-
EFA77913        -------RKLHTWEWDYIQQNHFG----------SKA-----SLTKTECKE----------FWAKFGPILQTIH-FKRHISSLWFEGLICGFISKNECN-
XP_004362589    -------RKLHTWEFDYIQQTHFA----------MKP-----RVTRQEVKE----------FWVKFGPILQTIH-FKRHISSLWFEGLIYGLISKNECN-
EFA75761        ------TDGANNHDDIYAALQTEQKKL--AGQIESELQTLNQMYSQTILEP-----N----QLCKLDILLQDLS-IQYKQLQLYQNELNYGPGGPELPV-
XP_004347383    ------QQPLSQRDLEYLTWKASD----------LIIN---ESITWKRLTK-EALPDRTFTFWDWFFGAEDVI---KRHMLETWQDGLIMGFCNKQQAH-
XP_004339235    -------RRLNHGEFQYIHYKFFG---------GQLN------VTQQQAGR----------FWSWFGQVVQTLR-FKRHIANMWFVGLIYGFITKNACT-
XP_004352957    Q----------FWSWFGKGLHILR-YQRHVGNLWKSGLVYGFIAKDAVH-RALSNER-PGSFLVRFSEN-HAGSFAVAYKHIEALT--------------
Q24151          ------GRSLTIDNLDFLYEKLQR---------EERSE----YITWNQFCK-EPMPDRSFTFWEWFFAIMKLT---KDHMLGMWKAGCIMGFINKTKAQT
XP_001866605    ------DRPLSAENLHFLGVKATR-------RKLPFPVPDAELVTRAQFCR-DLIPARPFTFWEWFYAAIKVT---RDSLKDIWNDGHMVGFVDKARAEQ
BAE06716        PGSASEGIGLTHEAKKYLTVKLLG--------TDAVDP--HTHVSWRQFNR-ENLPGKGFTFWTWFFKVMELVS--SPYVKKFWNLRYITGFIGKAECQ-
XP_004338884    -------RPLSNKDIEYLHLTKFNGKMRYVPILSLHRHLHLHLVLYSTRKWTSINQEDYDQFWAWFGTILYKIRNHQKHILPMWIKGLIYGFLSREDSDR
Q9NAD6          -----ARRPLSDSDLAYLSEKMLMP------NVADQKP-----ITFHRFAKQAMRDDLPFSFWEWFFSIMQLI---KQKLLKFWDEGWCIGFISKNDASQ

                ------XRGLSXENLXYLAQKLFG-------XXEDYSX---XXVSWSQFNK-ENLPGRNFTFWXWFDGILELL---KKHLKPLWNDGLIXGFVSKQQAX-
```

```
                        1310      1320      1330      1340      1350      1360      1370      1380      1390      1400
XP_005059708    DLLINKP-DGTFLLRFSDS-EIGGITIAWKFDSS-------------------E-------------------------------RMFWNLMPFTTRDFS
ACM68934        DLLISKP-DGTFLLRFSDS-EIGGITIAWKFDSA-------------------E-------------------------------RMFWNLMPFTTRDFS
XP_006026413    DLLINKP-DGTFLLRFSDS-EIGGITIAWKFDSH-------------------EFA---------------------------LWRMFWNLMPFTTRDFS
XP_003222527    DLLINKP-DGTFLLRFSDS-EIGGITIAWKFDNT-------------------D-------------------------------RMFWNLMPFTTRDFS
NP_001084677    DMLVNKR-DGTFLLRFSDS-EIGGITIAWKFELP-------------------D-------------------------------RALWNLMPFTARDFS
AAI70126        DMLVNKR-DGTFLLRFSDS-EIGGITIAWKFELP-------------------D-------------------------------RALWNLMPFTARDFS
NP_003143       DLLINKP-DGTFLLRFSDS-EIGGITIAWKFDSP-------------------E-------------------------------RNLWNLKPFTTRDFS
DAA18426        DLLINKP-DGTFLMRFSDS-EIGGITIAWKFDSP-------------------D-------------------------------RNLWNLKPFTTRDFS
AAC50491        DLLINKP-DGTFLLRFSDS-EIGGITIAWKFDSQ-------------------E-------------------------------RMFWNLMPFTTRDFS
NP_035619       DLLINKP-DGTFLLRFSDS-EIGGITIAWKFDSQ-------------------E-------------------------------RMFWNLMPFTTRDFS
DAA18427        DLLINKP-DGTFLLRFSDS-EIGGITIAWKFDSQ-------------------E-------------------------------RMFWNLMPFTTRDFS
NP_001157534    DLLINKP-DGTFLLRFSDS-EIGGITIAWKFDSP-------------------D-------------------------------RNLWNLKPFTTRDFS
NP_001003984    DMLMSKP-NGTFLLRFSDS-EIGGITIAWVAENP-------------------NKA---------------------------GERMVWNLMPFTTKDFS
XP_003723422    DLLLSQQ-VGCFLLRFSDS-EIGGITIAWLAEDA-------------------N-T---------------------------GERQVYNLQPFTADDFN
XP_002733524    ELLLSRA-NGTFMLRYSDG-SIGGVTIAWVAQDP-------------------N-T---------------------------GERQVWNLQPFTSEDFN
ACA79939        EMLKNSK-SGTFLLRFSDS-ELGGVTIAWMYEDT-------------------TKA---------------------------CQRDVFMLQPFTSKAFA
EFN82765        EMLSNCT-PGTFLMRFSDS-ELGGITIAWV-AD---------------------------------------------------QNDVFMLQPFTSKDFA
ACZ25562        DWLSNRP-MGTFLLRFSDS-EIGGITIAWTGER----------------------------------------------------QEVWNLAPFTSKDFH
P42226          SLLLNEP-DGTFLLRFSDS-EIGGITIAHVIRGQ-------------------D-----------------------------GSPQIENIQPFSAKDLS
NP_001192430    SLLLNEP-DGTFLLRFSDS-EIGGITIAHVIRGQ-------------------D-----------------------------GSPQIENIQPFSAKDLS
P52633          SLLLNEP-DGTFLLRFSDS-EIGGITIAHVIRGQ-------------------D-----------------------------GSSQIENIQPFSAKDLS
NP_001192618    RLLKKTV-SGTFLLRFSET-LEGGITCSWVEHQD-------------------DD-----------------------------EVLINSVQPFTKEVLQ
P52630          RLLKKTM-SGTFLLRFSES-SEGGITCSWVEHQD-------------------DD-----------------------------KVLIYSVQPYTKEVLQ
Q9WVL2          RLLKKML-SGTFLLRFSET-SEGGITCSWVEHQD-------------------DH-----------------------------KVEIYSVQPYTKEVLQ
NP_001077161    LLLKDKM-PGTFLLRFSES-HLGGITFTWVDHSE-------------------NG-----------------------------EVRFHSVEPYNKGRLS
NP_003142       LLLKDKM-PGTFLLRFSES-HLGGITFTWVDHSE-------------------SG-----------------------------EVRFHSVEPYNKGRLS
AAH98499        LLLKDKM-PGTFLLRFSES-HLGGITFTWVDQSE-------------------NG-----------------------------EVRFHSVEPYNKGRLS
P40763          AILSTKP-PGTFLLRFSESSKEGGVTFTWVEKDI-------------------SG-----------------------------KTQIQSVEPYTKQQLN
```

```
P42227          AILSTKP-PGTFLLRFSESSKEGGVTFTWVEKDI-------------------SG-----------------------------KTQIQSVEPYTKQQLN
NP_001012689    AILSTKP-PGTFLLRFSESSKEGGVTFTWVEKDI-------------------SG-----------------------------KTQIQSVEPYTKQQLN
Q7ZXK3          ALLSPKP-PGTFLLRFSESSKEGGITFTWVEKDI-------------------SG-----------------------------KTQIQSVEPYTKQQLN
Q9PVX8          AILSTKP-PGTFLLRFSESSKEGGITFTWVEKDI-------------------SG-----------------------------KTQIQSVEPYTKQQLN
P42224          ALLKDQQ-PGTFLLRFSESSREGAITFTWVERSQ-------------------NGG----------------------------EPDFHAVEPYTKKELS
AAI51379        ALLKDQQ-PGTFLLRFSESCREGAITFTWVERSQ-------------------NGG----------------------------EPYFHAVEPYTKKELS
AAA19454        ALLKDQQ-PGTFLLRFSESSREGAITFTWVERSQ-------------------NGG----------------------------EPDFHAVEPYTKKELS
XP_004355663    EALRNEE-MGTFLIRFSER-HAGHFAIGYKVDD-------------------PDPE---------------------------KRIRHYLVKADDT-AGA
EFA83377        EALKNEE-MGTFLIRFSER-HAGHFAIGYKVDD-------------------PDPE---------------------------KRIRHYLVKADDT-AGA
XP_003295153    EALRNED-QGTFLIRFSER-HAGHFAVGYKVDD-------------------PDPE---------------------------KRIRHYLVKADDT-AGA
CAC33514        EALRNEE-QGTFLIRFSER-HAGHFAVGYKVDD-------------------PDPE---------------------------KRIRHYLVKADDT-AGA
XP_004336217    AALINEE-VGTFLIRLSER-HPGLFAVGYKTDD-------------------ADVQ---------------------------KSVRHYLVQPEDT-AGA
XP_003291486    DALQGQD-PGTFIIRFSER-NPGQFGIAYIG---------------------IELP---------------------------PRIKHYLVQPNDT-AAA
XP_640661       DALQNQD-PGTFIIRFSER-NPGQFGIAYIG---------------------VEMP---------------------------ARIKHYLVQPNDT-AAA
XP_004366323    DALTNQD-PGTFIIRFSER-NPGQFGIAYIG---------------------SEVP---------------------------HRIKHYLVQPNDT-AAA
EFA82265        DALTNQD-PGTFIIRFSER-NPGQFGIAYIG---------------------MEVP---------------------------HRIKHYLVQPNDT-AAA
XP_004360534    NILKGQE-PGTFVILFSES-FAGQLEISYVSLDKQQESPLSLSSSLSNSSSAIPPPSLASATTSTTDINGGSGIDQSSQSSATKIKHYLVQPNDT-SGS
XP_646834       QILKNQD-VGTFVILFSEA-FPGQLEISYVGTD-QKDS-------LSKSSNDLQSP----TTTTTTT--------------TSTRVKHYLVQANDT-SGS
XP_003283502    SFLTNLP-EGSFLIRFSDS-VPGSFAVAYVTND--------------------DSE----------------------------PVKHYLVKPED--IGA
XP_643781       SYLTTLP-EGSFLIRFSDS-VPGAFAVAYVTND--------------------ESD----------------------------RVKHYLVKPDD--IGA
EFA77913        SSLYPAE-EGSFLIRFSDS-LPGSFAVAYVTND--------------------ENE----------------------------RVKHYLVRPEE--IGP
XP_004362589    SILYNSP-EGSFLIRYSDS-LPGSFAVAYVTND--------------------EQE----------------------------RVKHFLVKPEE--IGA
EFA75761        ALVITKQ-PFPMVISKFKQLQEDHLTVQLLVGSN--VDIISYSPIRAELIFHSKALTKGSATLIGGIGSVNSGTGGSGGNSALKKHIEKDTQSIDPVKGN
XP_004347383    DLLINCV-PGTFLLRFSDS-EIGGLTVAWITEDE--------------------RG----------------------------MRQVFNLHPWFAKDFA
XP_004339235    EILKNEE-IGTFVIRFSEN-HPGLFAVAYVDDDP------------------------------------------------YERVKHYLVKPED--ISS
XP_004352957    --FPLP----------------------------EVKHYLVKPDD-IAGPKKTLPDFLQE----------------------------------------
Q24151          DLLRSVYGIGTFLLRFSDS-ELGGVTIAYVNEN----------------------------------------------------GLVTMLAPWTARDFQ
XP_001866605    DLRQHPP--GTFLLRFSDS-QQGGITIAYVTNEP--------------------------------------------------SRRIQHINPFIG--KD
BAE06716        NYLLTCP-LGAFMFRFSDS-QLGALSISCYTYCEK--------------------TK---------------------------RKEVGHLEPDTIKRLQ
XP_004338884    LLLTQGALPGSFLIRFSDR-CAGQFVVVYVTSSPNKK---------------ADGG---------------------AGGGDDREVKHYLINPED--IEK
Q9NAD6          SMMMCQH--SSFLLRFSDS-QTGAVSIGFVCEEA-------------------D-----------------------------GQKIPFHLAPFTIKDLD

                DLLXNKP-PGTFLLRFSDS-EIGGITIAWVXXDX-------------------DX-----------------------------XRXHYNVQPFTTXDFS

                         1410      1420      1430      1440      1450      1460      1470      1480      1490      1500
XP_005059708    IRSLADRLG----------DLSYLIYVFPD------RPKDEVFS--KYYTPV--------LSKAVDGYVKPQIKQVV-----------------PEFVNA
ACM68934        IRSLADRLG----------DLSYLIYVFPD------RPKDEVFS--KYYTPV--------LSKAVDGYVKPQIKQVV-----------------PEFVSA
XP_006026413    IRSLADRLG----------DLSYLIYVFPD------RPKDEVFS--KYYTPVPC---ESTPAKAVDGYVKPQIKQVV-----------------PEFATA
XP_003222527    IRSLADRLG----------DLPYLLFVYPD------RPKEEVFS--KYYMPV--------LAKAVDGYVKPQIKQVV-----------------PEFATT
NP_001084677    VRSLADRLG----------DLNYLIYVYPD------RPKDEVFS--KYYTPVLC---N---PSKTDGYVKPQIKQVV-----------------PEFVAS
AAI70126        VRSLADRLG----------DLNYLIYVYPD------RPKDEVFS--KYYTPVLC---N---PSKTDGYVKPQIKQVV-----------------PEFVAN
NP_003143       IRSLADRLG----------DLSYLIYVFPD------RPKDEVFS--KYYTPV--------LAKAVDGYVKPQIKQVV-----------------PEFVNA
DAA18426        IRSLADRLG----------DLNYLIYVFPD------RPKDEVFS--KYYTPV--------LAKAVDGYVKPQIKQVV-----------------PEFVSA
AAC50491        IRSLADRLG----------DLNYLIYVFPD------RPKDEVYS--KYYTPVPC---ESATAKAVDGYVKPQIKQVV-----------------PEFVNA
NP_035619       IRSLADRLG----------DLNYLIYVFPD------RPKDEVYS--KYYTPVPC---EPATAKAADGYVKPQIKQVV-----------------PEFANA
DAA18427        IRSLADRLG----------DLSYLIYVFPD------RPKDEVYS--KYYTPVPC---EPATAKAVDGYVKPQIKQVV-----------------PEFVSA
NP_001157534    IRSLADRLG----------DLNYLIYVFPD------RPKDEVFA--KYYTPVLG---SS-MHPQMPEPAPPTWTRLL-----------------PQSCAL
NP_001003984    IRSLADRIS----------DLNHLLFLYPS------QPKEEVFS--RYCTPPN-------SKAVGDGYVKPEIKQVV-----------------KVEFSS
XP_003723422    IRSLADRIH----------DLSHLTHLYPD------KPKDTAFG--QYYTTDP-----EVPPQGDGGYVPTSLVS-------------------------
XP_002733524    IRSLADRIL----------DLPQLVNLYPD------IPKDLAFA--KYTKKQP-----EVDTKTQDGYVPSGLVSTV-----------------QLPAAS
```

ACA79939	IRPLADVIA----------DLNYLLYLYPN------VPKDQAFG--KYYTPL-----GEQQPTTNNGYVKPQLKTHV-----------------PGWS--
EFN82765	IRCLADRVN----------DLQYLLFLYPE------YTKDQAFS--KYYTPF-----NDNQATSTNGYVKPLLVTHV-----------------PGWSAP
ACZ25562	IRGLADRIK----------DLNSLVYLYPN------KPKDSVFS--KYYT-------STVEPVINDGYIRANLKTTL-----------------PDHLTT
P42226	IRSLGDRIR----------DLAQLKNLYPK------KPKDEAFR--SHYKPE------Q-MGKDGRGYVPATIKMTVERDQPLPTPELQMPTMVPSYDLG
NP_001192430	IRSLGDRIR----------DLAQLKNLYPK------KPKDEAFR--SHYKPE------Q-MGKDGRGYVPATIKMTVERDQPLPTPEPQMPTMVPSYDLG
P52633	IRSLGDRIR----------DLAQLKNLYPK------KPKDEAFR--SHYKPE------Q-MGKDGRGYVSTTIKMTVERDQPLPTPEPQMPAMVPPYDLG
NP_001192618	SLPLTKIISQYQMLTEENIPENPLRFLYPR------IPRDEAFG--CYSQEKIN---PEERKK----YLKHKLIFVSNRQVDELQ---------QLPELK
P52630	SLPLTEIIRHYQLLTEENIPENPLRFLYPR------IPRDEAFG--CYYQEKVN---LQERRK----YLKHRLIVVSNRQVDELQ---------QPLELK
Q9WVL2	SLPLTEIIRHYQVLAEENIPENPLRFLYPR------IPRDEAFG--CYYQEKVN---LEEQEE----YLKHKLIVISNRQVDELQ---------QPLELK
NP_001077161	ALPFADILRDYKVIMAENIPENPLKYLYPD------IPKDKAFGKHYSSQPCEVSRPTEKGDK---GYVPSVFIPISTISSSR-----------SDSTEP
NP_003142	ALPFADILRDYKVIMAENIPENPLKYLYPD------IPKDKAFGKHYSSQPCEVSRPTERGDK---GYVPSVFIPISTIRS--------------DSTEP
AAH98499	ALPFADILRDYKVIMAENFPENPLKYLYPD------IPKDKAFGKHYSSQPCEVSRPTERGDK---GYVPSVFIPISTIRS--------------DSTEP
P40763	NMSFAEIIMGYKIMDATNILVSPLVYLYPD------IPKEEAFG--KYCRPESQE-HPEADPGSAAPYLKTKFICVTPTTCS------------NTIDLP
P42227	NMSFAEIIMGYKIMDATNILVSPLVYLYPD------IPKEEAFG--KYCRPESQE-HPEADPGSAAPYLKTKFICVTPTTCS------------NTIDLP
NP_001012689	NMSFAEIIMGYKIMDATNILVSPLVYLYPD------IPKEDAFG--KYCRPESQE-HPEADPGSAAPYLKTKFICVTPTTCS------------NTIDLP
Q7ZXK3	SMSFAEIIMGYKIMDATNILVSPLVYLYPD------IPKEEAFG--KYCRPESQEHQEPTDPG-TAPYLRTMFICVTPTTC--------------TLDLP
Q9PVX8	NMSFAEIIMGYKIMDATNILVSPLVYLYPD------IPKEEAFG--KYCRPESQEHQEPTDPGSTAPYLKTKFICVTPTTCS------------STLDLP
P42224	AVTFPDIIRNYKVMAAENIPENPLKYLYPN------IDKDHAFGK-YYSRPKEAPEPMELDGPKGTGYIKTELISVSEVHPSRLQT--------TDNLLP
AAI51379	AVTFPDIIRNYKVMAAENIPENPLKFLYPN------IDKDHAFGK-YYSRPKE--EPMELDGPKGTGYIKTELISVSEVHPSRLQT--------TDNLLP
AAA19454	AVTFPDIIRNYKVMAAENIPENPLKYLYPN------IDKDHAFGK-YYSRPKEAPEPMELDDPKRTGYIKTELISVSEVHPSRLQT--------TDNLLP
XP_004355663	KKTLPDFLS----------ECPQFTKILQL------TIDPITGE------P----------------RLRNFPKDVV-----------------------
EFA83377	KKTLPDFLA----------ECPQFTKILQL------TIDPTTGE------P----------------RLRNFPKDVV-----------------------
XP_003295153	KKTLPDFLS----------ECPQFTKILQL------TIDVQTGE------P----------------RLRNFPKDVV-----------------------
CAC33514	KKTLPDFLS----------ECPQFTKILQL------TIDVSTGE------P----------------RLRNFPKDVV-----------------------
XP_004336217	KKTLPDFLF----------TCPAFQFLLVV------TSDVENGT------P----------------KLRKFAKDVA-----------------------
XP_003291486	KKTFPDFLS----------EHSQFVNLLQW------TKDAN-GN------P----------------RFLKLHKDTA-----------------------
XP_640661	KKTFPDFLS----------EHSQFVNLLQW------TKDTN-GA------P----------------RFLKLHKDTA-----------------------
XP_004366323	KKTFPDFLA----------EHPQFINILQW------SKGSD-GL------P----------------RFLKSHKDTA-----------------------
EFA82265	KKTFPDFLA----------EHPQFINILQW------TKGPD-GL------P----------------RFLKSHKDTA-----------------------
XP_004360534	KRTLPDFLN----------ECPQFTHILQL------NVPSLTGTS-GTAVP----------------QFKKEPKNQI-----------------------
XP_646834	KRTLPDFLS----------ECNQFTHILQL------NIAMIPQT---ETIP----------------VFKREPKNVV-----------------------
XP_003283502	NKTLPDFLR----------ERHQFKTLYQV------DPSKR--------------------------SLHPKNKDTE-----------------------
XP_643781	NKTLPDFLR----------ERHQFKTLYQV------DPSKR--------------------------SLHPKSKDAE-----------------------
EFA77913	NKTLPDFLR----------ERYQFKTLYKL------DPPNK--------------------------LMKPVPKDEA-----------------------
XP_004362589	NKTLPDYLR----------ERYQFQVLYTL------DPPLK--------------------------TLKPVPKDTA-----------------------
EFA75761	AKFPIKFLTG------TRKGCVKLHFVLQI------KTTDGHLNVSTLSHTES-----------TIPPFRRELKNTV-----------------------
XP_004347383	IRALADRIH----------DLPQLQFLFPD------TPKDAVFG--RHYSVET-----AHVTSNNPDYVRSSIAAVIPG-------------SVPSFTMP
XP_004339235	NKSLPDFLR----------EKPQFLYVNQL------DPATGELH----KLPKDK--VLEGYYSKRQLHGKPSNGYVLL----------------------
XP_004352957	--
Q24151	VLNLADRIR----------DLDVLCWLHPSDRNASPVKRDVAFG--EFYSKRQE--PEPLVLDPVTGYVKSTLHVHVCR----------------N----
XP_001866605	AVNAINAIR----------DLPQLKFVYPG------VPKEEAFG--RYFRPKVLPVAGYVPAEPAAPNLATQADGLANNQSVNSSIAKLGGLPRSEYYLT
BAE06716	TRSIPDMVK----------DLDYLLYLGAD------IPKDRAFG--PYYTPEFR------VESAQKHDYWPKSLQVVT-------------------KNN
XP_004338884	KSTLPDFLR----------DCENLWFLLQVVR----DHDTGVVS------------------------LRPKNKDEL-----------------------
Q9NAD6	QLSLASRIASCP-------QLKDIRYMYPA------IDKEEMLR--FFESEERHR---VGGGDSPTGYIQSEIVMVAKTNGNFRRMSNAPSMFGADSPSP
	IRSLA**D**RLR----------DLNQ**L**XYLYPD------IPKDEAFG--KYYT**P**XX-----X-XXKXXXGYVKPXIKXVV-----------------PXXXXX
	1510 1520 1530 1540 1550 1560 1570 1580 1590 1600
XP_005059708	SGDSASG--GATYMDQAPSPA---------VCSQPHYNMYTQN---------PDPVLD
ACM68934	SGDAVPG--GGTYMDQAPSPA---------VCSHPHYNMYTQN---------PETVLD

```
XP_006026413    SGDSTPG--------------------------------------------SATYMDQAPSPA---------VGSQTHYNMYTQN---------PEAVLD
XP_003222527    SGDSAGG--------------------------------------------GTTYMEQASSPA---------VCPQSHYNMYAQN---------SDSVLD
NP_001084677    --DVMP---------------------------------------------PSTYMDQAPSPA---------VCSQSHYNLYQQN---------PDSVLD
AAI70126        --DVMP---------------------------------------------PSTYMDQAPSPA---------VCSQSHYNLYQQN---------PDSVLD
NP_003143       SADAGGS--------------------------------------------SATYMDQAPSPA---------VCPQAPYNMYPQN---------PDHVLD
DAA18426        SADSAGS--------------------------------------------NATYMDQAPSPA---------VCPQPHYNMYPQN---------PDPVLD
AAC50491        SADAGGG--------------------------------------------SATYMDQAPSPA---------VCPQAHYNMYPQN---------PDSVLD
NP_035619       STDAGSG---------------------------------------------ATYMDQAPSPV---------VCPQAHYNMYPPN---------PDSVLD
DAA18427        SADSAGG--------------------------------------------SATYMDQAPSPA---------VCPQPHYNMYPQN---------PDPVLD
NP_001157534    NLTTTCT--------------------------------------------HPTLTLSLTKMASLTWMR-AWMLPGTWKNFYAG----------PWTVST
NP_001003984    PNPEPSP--------------------------------------------GNSFMDHAASPT---------VNQHHNFTIYPAM---------NDTMID
XP_003723422    HIPHMPG--------------------------------------------GPPQHHSQEMGNR-E------PMSPPSQQLMTSS---------SLRVSS
XP_002733524    MMPMSPL--------------------------------------------APRMYEEPASPAQ-S------IINPGSVQSTAST---------DTTMAD
ACA79939        ---------------------------------------------------GDPMDSYPNTPQT------MYGVMGGPPSVS-SN---------PSDCVS
EFN82765        GLGNQTPSHSSVVGVG-------------------------------GNGQGGPGGSYPATPST------VFQPHSPDPSVTRDT---------PSTASS
ACZ25562        ---------------------------------------------------SGEMERQPFSPET------VYDMNPNSVQNMDEL---------PMTPDM
P42226          MAPDSSMSMQLGPDMVPQVYPPHSHSIPPYQGLSPEESVNVLSAFQEPHLQMPPSLGQMSLPFDQPHPQGLLPCQPQEHAVSSPD---------PLLCSD
NP_001192430    MAPDSSMNLQLGPDMVPQVYPPRSHSIPSYPALPREESVNMLPAFQEPHLPMPPNLSQMSLPFDQPHPQGLLPCPPQDHAVSSPE---------PLLCSD
P52633          MAPDASM--QLSSDMG---YPP--QSIHSFQSL--EESMSVLPSFQEPHLQMPPNMSQITMPFDQPHPQGLLQCQSQEHAVSSPE---------PMLCSD
NP_001192618    LEPE-LES-------------------------------------------LELGLGFTSGPEHGPG----LDLEPLLEAGLDLS--------MEPMLEP
P52630          PEPE-LES-------------------------------------------LELELGLVPEPELS------LDLEPLLKAGLDLGPE--LESVLESTLEP
Q9WVL2          QDSESLEVNAELLLAH----------------------------------DQELPLMMQTGLVLGTE----LKVDPILSTAPQVLLEPAPQVLLEPAPQV
NP_001077161    HSPS---DL------------------------------------------LPMS---PSVYAVLR-----ENLSPTTIETAMKS---------PYSAE-
NP_003142       HSPS---DL------------------------------------------LPMS---PSVYAVLR-----ENLSPTTIETAMKS---------PYSAE-
AAH98499        QSPS---DL------------------------------------------LPMS---PSAYAVLR-----ENLSPTTIETAMNS---------PYSAE-
P40763          MSPRTLDSL------------------------------------------MQFGNNGEGAEPSAG-----GQFESLTFDMELTS---------ECATSP
P42227          MSPRTLDSL------------------------------------------MQFGNNGEGAEPSAG-----GQFESLTFDMDLTS---------ECATSP
NP_001012689    MSPRTLDSL------------------------------------------MQFGNNGEAAEPSAG-----GQFESLTFDMELTS---------ECATSP
Q7ZXK3          MSPGTFDSV------------------------------------------MQFP--GEGSESGNG-----NQFETLTFDVDLPS---------ECAASP
Q9PVX8          MSPRTLDSL------------------------------------------MQFP--GEGADSSAG-----NQFETLTFDMELTS---------ECASSP
P42224          MSP------------------------------------------------EEFD---EVSRIVGS-----VEFDSMMNTV-------------------
AAI51379        MSP------------------------------------------------EEFD---EVSRMVGS-----VEFD-MMNAV-------------------
AAA19454        MSP------------------------------------------------EEFD---EMSRIVG------PEFDSMMSTV-------------------
XP_004355663    ---------------------------------------------------LEPYYSKREALP--------------ATNGYDS-------------LPV
EFA83377        ---------------------------------------------------LEPYYSKREALP--------------ATNGYDS-------------LPM
XP_003295153    ---------------------------------------------------LEPYYSKRETLP--------------ATNGYDS-------------LPT
CAC33514        ---------------------------------------------------LEPYYSKRETLP--------------ATNGYDS-------------LPT
XP_004336217    ---------------------------------------------------LQPYYSKKNPVA--------------KAKGYDDEIP----------IPN
XP_003291486    ---------------------------------------------------LGSFAPKKSQPP--------------PIGGYEP-------------LSS
XP_640661       ---------------------------------------------------LGSFAPKRTAPV--------------PVGGYEP-------------LNS
XP_004366323    ---------------------------------------------------LNSFAPRRAQPT--------------PVGGYEP-------------LSS
EFA82265        ---------------------------------------------------LGSFAPKKAQPA--------------PIGGYEP-------------LGN
XP_004360534    ---------------------------------------------------LEPYYSKRQNPQSY------------LGNGYDP-------------LN-
XP_646834       ---------------------------------------------------LEPYYSKRQNSQNI------------LGSGYDP-------------LF-
XP_003283502    ---------------------------------------------------LEPFYSKRIKINANA------------NPGYVSG------------L--
XP_643781       ---------------------------------------------------LEPYYSKRLK-QQQT------------NPGYVS-------------L--
EFA77913        ---------------------------------------------------FKDFYSKRITKVG--------------NPGYVSE------------DSS
XP_004362589    ---------------------------------------------------FASYYSKRIQEREKAK----------TNPGYVSG------------L--
EFA75761        ---------------------------------------------------LEPYYSKRQNSQN------------FLGSGYDPLT--------------
XP_004347383    IVNDQFLMKAD---------------------------------------DSSSSMMHPTSSNLLDLVGMGVGMGVGDADAGQMSS-----LDWSTFLES
XP_004339235    ----------------------------------------------------------------------------------------------------
```

```
XP_004352957    ----------------------------------------------------------------------------------------------------
Q24151          GENGSTS--------------------------------------------GTPHHAQESMQLGNGD---FGMADFDTITNFENF---------------
XP_001866605    QNGKRLTDVTPSPLPDVPIRLQERLPAGKGGFG-------------SMLRAIGAQIEKTTNREACRDLSGRRLRDINEEKRLKAYLDKQKEADTEDEAAK
BAE06716        GTTST----------------------------------------------FIRSMKQPNSPDMGPHVQLKSSMSPPTFGSPGTPLS-------PLDLLS
XP_004338884    ---------------------------------------------------LASFYSREKSQT--------------LLLGYDNRQP-------------
Q9NAD6          LSVQSKLDWSPGEVHQN------------------------------HMMEMSDELGQILTVSDMSGDVETLLGPAFKNNITNYNPHDGNHQHNLHFVDM

                XXPX-X---------------------------------------------LXPYXSQXXSPAX--------XXXXXXXNGYXXX---------PXXLXX


                         1610      1620      1630      1640      1650      1660      1670      1680      1690      1700
XP_005059708    PEGDFD--LE---------------------------------------------------------------------------DT--MDVARHVEELL
ACM68934        PEGDFD--LD---------------------------------------------------------------------------DT--MDVAQHVEELL
XP_006026413    PEGDFD--LD---------------------------------------------------------------------------DT--MDVARHVEELL
XP_003222527    PDGDFD--LD---------------------------------------------------------------------------DS--MDVARHVEELL
NP_001084677    PDGDLD--LD---------------------------------------------------------------------------D---MDVARHVEELL
AAI70126        PDGDLD--LD---------------------------------------------------------------------------D---MDVARHVEELL
NP_003143       QDGEFD--LD---------------------------------------------------------------------------ET--MDVARHVEELL
DAA18426        QDGEFD--LD---------------------------------------------------------------------------ET--MDVARHVEELL
AAC50491        TDGDFD--LE---------------------------------------------------------------------------DT--MDVARRVEELL
NP_035619       TDGDFD--LE---------------------------------------------------------------------------DT--MDVARRVEELL
DAA18427        NDGDFD--LD---------------------------------------------------------------------------DT--IDVARRVEELL
NP_001157534    PASPHL--LV---------------------------------------------------------------------------SS--PPLEAPCPERW
NP_001003984    AEGEFD--LD---------------------------------------------------------------------------ET--MDMARQVEEFL
XP_003723422    SDGMVG----------------------------------------------------------------------------------TTSMDQNLDPLE
XP_002733524    MNSAAN--AE---------------------------------------------------------------------------IDLTELTVEDINALA
ACA79939        TVP-----------------------------------------------------------------------------P-----YNE----NEYDQFL
EFN82765        YAPGLG--QSGVG------------------------------------------------------------------RPSSDVDYVELMSQNELTSID
ACZ25562        SRG-----------------------------------------------------------------------------------LAEMD-LNHLTEFL
P42226          VTMVEDSCLSQPV------------------------------------------------------------------TAFPQGTWIGEDIFPPLLPPT
NP_001192430    VTMAEDSCLNQPV------------------------------------------------------------------GGFPQGTWVREDMFPPLLPPT
P52633          VTMVEDSCLTQPV------------------------------------------------------------------GGFPQGTWVSEDMYPPLMPPT
NP_001192618    TLEPT----LGEVSQGVLEP------HPGPQLQLEPIPELELQPMP--------------------------------------EPDLPYDLRHLNTEDM
P52630          VIEPT----LCMVSQTVPEP------DQGP----------VSQPVP--------------------------------------EPDLPCDLRHLNTEPM
Q9WVL2          LLEPAPQVLLEPAPQVLLEPAPQVLLEPAPQVLLEPAPQVQLEPAPQVLLELAPQVLLEPAPQVLLELAPQVQLEPAHLLQQPSESDLPEDLQQISVEDL
NP_001077161    ----------------------------------------------------------------------------------------------------
NP_003142       ----------------------------------------------------------------------------------------------------
AAH98499        ----------------------------------------------------------------------------------------------------
P40763          M---------------------------------------------------------------------------------------------------
P42227          M---------------------------------------------------------------------------------------------------
NP_001012689    M---------------------------------------------------------------------------------------------------
Q7ZXK3          M---------------------------------------------------------------------------------------------------
Q9PVX8          M---------------------------------------------------------------------------------------------------
P42224          ----------------------------------------------------------------------------------------------------
AAI51379        ----------------------------------------------------------------------------------------------------
AAA19454        ----------------------------------------------------------------------------------------------------
XP_004355663    LN--------------------------------------------------------------------------------------------------
EFA83377        LN--------------------------------------------------------------------------------------------------
XP_003295153    IIN-------------------------------------------------------------------------------------------------
CAC33514        IL--------------------------------------------------------------------------------------------------
XP_004336217    MLSP------------------------------------------------------------------------------------------------
```

```
XP_003291486  ----------------------------------------------------------------------------------------------------
XP_640661     ----------------------------------------------------------------------------------------------------
XP_004366323  ----------------------------------------------------------------------------------------------------
EFA82265      ----------------------------------------------------------------------------------------------------
XP_004360534  ----------------------------------------------------------------------------------------------------
XP_646834     ----------------------------------------------------------------------------------------------------
XP_003283502  ----------------------------------------------------------------------------------------------------
XP_643781     ----------------------------------------------------------------------------------------------------
EFA77913      NKHYSHFKLLYSTSTTCLIIRILKIRN-------------------------------------------------------------------------
XP_004362589  ----------------------------------------------------------------------------------------------------
EFA75761      ----------------------------------------------------------------------------------------------------
XP_004347383  LAEPIPQLQHDGI---------------------------------------------------------------------------------------
XP_004339235  ----------------------------------------------------------------------------------------------------
XP_004352957  ----------------------------------------------------------------------------------------------------
Q24151        ----------------------------------------------------------------------------------------------------
XP_001866605  LQKKVDKLLAKPKHEFHDARYNQARTDLTQNVDEAVQEGLRRAAEVAAAGEGGSKRKAKDDGS-------DAK------GKKKKAKGALWLGADDLGSSS
BAE06716      PNPSLHGE--------------------------------------------------------------------------------------------
XP_004338884  ----------------------------------------------------------------------------------------------------
Q9NAD6        SQQGMMQQHHNQFYPS------------------------------------------------------------------------------------

              XXX-------------------------------------------------------------------------------------------------


                      1710      1720      1730      1740      1750
XP_005059708  RRPVD-SQWIPHAQS-----------------------------------
ACM68934      RRPMD-SQWIPHAQS-----------------------------------
XP_006026413  RRPMD-HQWIPHAQS-----------------------------------
XP_003222527  RRPMD-SQWISHAQS-----------------------------------
NP_001084677  RRPMDNSQWIPHAQS-----------------------------------
AAI70126      RRPMDNSQWIPHAQS-----------------------------------
NP_003143     RRPMD-SLDSRLSPPAG------------LFTSARGSLS-----------
DAA18426      RRPMD-SLEPS---------------------------------------
AAC50491      GRPMD-SQWIPHAQS-----------------------------------
NP_035619     GRPMD-SQWIPHAQS-----------------------------------
DAA18427      GRPMD-SQWIPHAQS-----------------------------------
NP_001157534  TPCFSWKTTFSVRSPRQ------------L--------------------
NP_001003984  RQPME-TTWSGQQS------------------------------------
XP_003723422  ELPILEDYDDPQAGIME------------DLLRWSASTS-----------
XP_002733524  NISFTDDFNIEGAEEFD------------TIMAEDLDSFLSRVL------
ACA79939      EN---LSEADFPDMNF-------------DFLQTNFMKPQ----------
EFN82765      EN---LNLDHLNNFSFS------------EFMQS-YNKPQ----------
ACZ25562      PDDIGIPEIDVQQLLVN------------SYIDSRVVMD-----------
P42226        EQDLTKLLLEGQGESGGGSLGAQPLLQPSHYGQSGISMSHMDLRANPSW-
NP_001192430  EQDLTKLLLEGQGESGGGSLGPQPLLQPSPYGQSGISMSHLDLRANPSW-
P52633        EQDLTKLLLENQGEAGG-SLGSQPLLQPSPYGQSGISLSHLDLRTNPSW-
NP_001192618  EIFRNSMRIEEIMPNGDPVLPCQNTMDEADIFNPSHFYADGPL--SPSDY
P52630        EIFRNCVKIEEIMPNGDPLLAGQNTVDEVYVSRPSHFYTDGPL--MPSDF
Q9WVL2        KKLSN-PSTEYITTNENPMLAGESSGDETSIPYHSHFDADGLLGWTLDTF
NP_001077161  --------------------------------------------------
NP_003142     --------------------------------------------------
AAH98499      --------------------------------------------------
```

```
P40763         --------------------------------------------------
P42227         --------------------------------------------------
NP_001012689   --------------------------------------------------
Q7ZXK3         --------------------------------------------------
Q9PVX8         --------------------------------------------------
P42224         --------------------------------------------------
AAI51379       --------------------------------------------------
AAA19454       --------------------------------------------------
XP_004355663   --------------------------------------------------
EFA83377       --------------------------------------------------
XP_003295153   --------------------------------------------------
CAC33514       --------------------------------------------------
XP_004336217   --------------------------------------------------
XP_003291486   --------------------------------------------------
XP_640661      --------------------------------------------------
XP_004366323   --------------------------------------------------
EFA82265       --------------------------------------------------
XP_004360534   --------------------------------------------------
XP_646834      --------------------------------------------------
XP_003283502   --------------------------------------------------
XP_643781      --------------------------------------------------
EFA77913       --------------------------------------------------
XP_004362589   --------------------------------------------------
EFA75761       --------------------------------------------------
XP_004347383   --------------------------------------------------
XP_004339235   --------------------------------------------------
XP_004352957   --------------------------------------------------
Q24151         --------------------------------------------------
XP_001866605   DDGDSDDGSDSAGSSSGVSSAKQTVGPTSEEDESGSADKEVEVASG----
BAE06716       --------------------------------------------------
XP_004338884   --------------------------------------------------
Q9NAD6         --------------------------------------------------

               --------------------------------------------------
```

B

Consensus sequence

MAXWXQXQQLXXXXLXQXQXLYGXXFPIEVRHYLAQWIESQXWXXXXDXDNPXXXXXAXQLLXQLXQQLQXKAXEQXGXXXFLLXXXL
XHXXTQLQXXYXXXPMELVRXIXHILXEEQRLVXXAXXXXXXXXXXXXXXMSQKQXXIXQXXEELRXXTQDTENXLKXLQQXQEXFIXQ
YQEXXRXQAQXXQLXXXXPQXXLQQXQXQLEXXLQXXAXXLXQXRXELAXKHQXTLXXLXKLQXXILDDE**L**IQW**K**RR**Q**QLAGNGG**P**PE
GX**L**DX**L**QNWCEX**L**AEIIWQNRQQIKXLEXLXQLLPXXGXXVXXXLXE**L**NXQITXLXSX**L**VTSTFIVEK**QPP**QVLKTQTKFQAXVRL**L**V
GGKHMXPPQVKVXIISEXQAKXLLXNXGXRKXSGEILNNXXXMEYXQXTGXLSAEFRNMS**L**KEIKXAXRXDRKGAESVTEEKFTIL**F**E
SQFSVGGNLVFQVETL**S**L**P**V**VVI**VNGS**Q**DXN**A**WATI**L**WDNA**F**AEPGRVPFAVPDKVLWXQLLEALNQKFKSEVX**R**G**L**SXEN**L**XY**L**AQ**K**
L**F**GXXEDYSXXXVSWSQ**F**NKENLPGRNFT**FW**X**WF**DGILELLKK**H**LKPL**W**ND**G**L**I**X**GF**VSKQQAXDL**L**XNKPP**GTFL**L**RFS**D**S**EI**G**GIT
IAWVXXDXDXXRXHYNVQ**P**FTTXDFSIRSLA**D**RLRDLNQ**L**XYLYPDIPKDEAFGKYYT**P**XXXXXKXXXGYVKPXIKXVVPXXXXXXXP
XXLXPYXSQXXSPAXXXXXXXXXNGYXXXPXXLXXXXX
